# Expanded assessment of xenobiotic associations with antinuclear antibodies in the United States, 1988–2012

**DOI:** 10.1016/j.envint.2022.107376

**Published:** 2022-06-27

**Authors:** Gregg E. Dinse, Caroll A. Co, Christine G. Parks, Clarice R. Weinberg, Guanhua Xie, Edward K.L. Chan, Linda S. Birnbaum, Frederick W. Miller

**Affiliations:** a Public Health & Scientific Research, Social & Scientific Systems, Durham, NC, USA; b Epidemiology Branch, National Institute of Environmental Health Sciences, National Institutes of Health, Research Triangle Park, NC, USA; c Biostatistics and Computational Biology Branch, National Institute of Environmental Health Sciences, National Institutes of Health, Research Triangle Park, NC, USA; d Department of Oral Biology, University of Florida, Gainesville, FL, USA; e Mechanistic Toxicology Branch, National Institute of Environmental Health Sciences, National Institutes of Health, Research Triangle Park, NC, USA; f Environmental Autoimmunity Group, National Institute of Environmental Health Sciences, National Institutes of Health, Research Triangle Park, NC, USA

**Keywords:** Autoimmune diseases, Autoimmunity, Dioxin-like mixtures, Environmental chemicals, National Health and Nutrition Examination, Survey, Risk factors

## Abstract

**Background:**

The prevalence of autoimmunity in the U.S. has increased recently for undetermined reasons. Little is known about associations between autoimmunity and environmental causes.

**Objectives:**

In a large representative sample of the U.S. population, we expanded our prior exploratory study of how exposures to selected xenobiotics and dioxin-like (DL) mixtures relate to antinuclear antibodies (ANA), the most common biomarker of autoimmunity.

**Methods:**

We analyzed cross-sectional data on 12,058 participants aged ≥ 12 years from three time periods of the National Health and Nutrition Examination Survey between 1988 and 2012, of whom 14% were ANA-positive. We used lognormal regression models and censored-data methods to estimate ANA associations with xenobiotic concentrations overall and in sex, age, and race/ethnicity subgroups. Our analyses adjusted for potential confounders and appropriately handled concentrations below detection limits.

**Results:**

Observed ANA associations were positive for most DL compounds and nonDL polychlorinated biphenyls (PCBs), negative for most phthalates, and mixed for other xenobiotic classes. After correcting for multiple comparisons, some associations remained statistically significant. In subgroup analyses, the most significant finding was a positive ANA association with N-acetyl-S-(2-hydroxy-3-butenyl)-L-cysteine (MHB2) in males, followed by positive associations with 2,2′,3,5′-tetrachlorobiphenyl (PCB 44), 2,2′,4,5′-tetrachlorobiphenyl (PCB 49), and 2,2′,3,4′,5′,6-hexachlorobiphenyl (PCB 149) in 12–19 year-olds, and with 3,4,4′,5-tetrachlorobiphenyl (PCB 81), 2,2′,3,3′,4,4′,5,5′,6-nonachlorobiphenyl (PCB 206), and N-acetyl-S-(phenyl)-L-cysteine (PMA) in Mexican Americans. Negative associations were found with mono-benzyl phthalate (MBzP) in 20–49 year-olds and mono-n-butyl phthalate (MnBP) in 12–19 year-olds. In overall analyses, combining stratum-specific results across race/ethnicity strata revealed a positive ANA association with PCB 81 and a negative ANA association with N-acetyl-S-(2-hydroxyethyl)-L-cysteine (HEMA).

**Discussion:**

This study identified potential associations between ANA and various xenobiotics. Further investigation to confirm these observations and elucidate effects of certain xenobiotics on immune regulation could have important mechanistic, preventive, and treatment implications for a variety of immune-mediated disorders.

## 1. Introduction

Autoimmune diseases (AID), defined by pathologic inflammation and autoantibodies or self-directed T-lymphocyte responses, are a heterogeneous group of disorders present in 8% or more of the U.S. population, and which appear to be increasing in prevalence (Bach 2002; Jacobson et al. 1997; Lerner et al. 2015; National Institutes of Health (NIH) 2005). Although these usually incurable diseases have a large and growing public health impact (NIH 2005), the risk factors and mechanisms leading to them remain poorly understood (Ellis et al. 2014). Genetic risk factors are becoming better defined, but the likely larger contributions from the environment – as suggested by low monozygotic twin concordance rates (Silman et al. 1993; Ulff-Moller et al. 2018) – are unclear (Miller 2011; Parks et al. 2014). A growing literature suggests that environmental factors, including drugs, tobacco smoke, silica, and other xenobiotics, are associated with AID and immune effects (Miller 2011). While some xenobiotic exposures have been linked to AID, potential associations between environmental chemical exposures and autoimmunity are understudied and often focus only on a single compound (Bigazzi 1997; Pollard et al. 2018).

The most common clinical biomarkers of AID are antinuclear antibodies (ANA) directed against many cellular components (Tan 1989). Autoantibodies usually precede AID and have higher population frequencies than the AID linked to them, and thus offer opportunities for epidemiologic studies of potential causal exposures (Dillon et al. 2020). We previously evaluated ANA associations with selected xenobiotics and mixtures by using National Health and Nutrition Examination Survey (NHANES) data from 1999 to 2004 (Dinse et al. 2016). In what may be the largest and most comprehensive assessment of possible ANA associations with xenobiotic exposures ever undertaken, we expanded our earlier study by analyzing data on nearly three times as many people (12,058) and over twice as many xenobiotics (192), as more NHANES cycles now have ANA data, and some xenobiotic data are only available in the additional cycles. Our earlier study stratified by sex and parity, with three subgroup analyses of males, nulliparous females, and parous females. However, as parity information was missing for many women, we decided in our current study to include all women and stratify by sex, age, and race/ethnicity, with eight subgroup analyses of males, females, ages 12–19 years, ages 20–49 years, ages ≥ 50 years, non-Hispanic Whites, non-Hispanic Blacks, and Mexican Americans.

Also, though we employ essentially the same analytical approach as before, the ANA data now come from a more appropriate assay, and we adjust for additional covariates. Prior studies conducted by other researchers usually modeled an indicator of ANA positivity as a covariate-adjusted function of xenobiotic concentration, which can be problematic when some concentrations are below the limit of detection (LOD). Un-detectable concentrations were typically excluded or replaced by constant values, such as LOD/2, which can lead to bias and inefficiency. In comparison, our approach reverses the roles of the ANA indicator and the xenobiotic concentration (i.e., we modeled concentration as a function of ANA status), while treating concentrations below the LOD as censored. This method improves the analysis by allowing application of censored-data techniques, by making full use of the observed data, and by avoiding bias and underestimation of variance (Dinse et al. 2014).

## 2. Methods

### 2.1. Study participants

We initially assessed ANA in 13,519 persons sampled from five NHANES cycles: 1988–1991 (4,727 persons), 1999–2000 (1,578 persons), 2001–2002 (1,192 persons), 2003–2004 (1,757 persons), and 2011–2012 (4,265 persons). Participants, all of whom provided biospecimens and completed questionnaires, were ≥ 12 years old and weighted to be nationally representative of the noninstitutionalized U.S. population. Available data included demographics, health covariates, measured factors such as height and weight, and constructed variables such as body mass index (BMI) and poverty income ratio (PIR). The NHANES protocol and this study were approved by the human subjects Institutional Review Board of the U.S. Centers for Disease Control and Prevention (CDC), and all participants gave written informed consent. For additional information about the NHANES, see their website (CDC 2017).

### 2.2. ANA assessment

Serum samples were evaluated by indirect immunofluorescence at a 1:80 dilution using the NOVA Lite HEp-2 ANA slide with DAPI kit (INOVA Diagnostics, San Diego, CA, USA) and a highly specific fluorescein isothiocyanate conjugated secondary antibody (goat anti-human IgG). Images captured via the NOVA View automated fluorescence microscope system (INOVA Diagnostics) were stored digitally. Immunofluorescence staining intensities were assigned integer grades from 0 to 4, relative to standard references, with nonzero grades indicating ANA positivity (Dinse et al. 2020). All samples were assayed in a single laboratory, using identical methods. At least two experienced evaluators made independent readings, blinded to participant characteristics and time period, and they agreed on over 95% of the grades; differences were resolved by consensus or adjudicated by a third blinded rater. Repeat testing of 200 random samples showed over 98% concordance.

### 2.3. Xenobiotic data

We initially considered all 253 xenobiotics measured in biospecimens from any NHANES cycle with ANA data (Supp. Table 1). Several analytes (e.g., manganese and selenium) are essential nutrients, but we refer to them as xenobiotics for consistency of nomenclature. Concentrations of many xenobiotics were below the assay’s LOD for some participants in one or more cycles (Browne and Whitcomb 2010). Though we used statistical methods developed to handle large proportions of these “nondetects” (Dinse et al. 2014; Helsel 2012), we excluded any xenobiotic for which greater than 90% of the participants had a concentration below the LOD or fewer than five ANA-positive participants had a detectable concentration. We made these exclusions to avoid unstable statistical estimates (Dinse et al. 2016). Ultimately, 192 xenobiotics had adequate data for analysis.

Our study included a diverse set of persistent and non-persistent xenobiotics or their metabolites, which were measured in serum, urine, or whole blood (see Supp. Table 1 footnotes). All biospecimens were analyzed for xenobiotics by the Division of Laboratory Sciences, National Center for Environmental Health, Atlanta, Georgia (CDC 2005; CDC 2015). See tables in CDC (2015) and Crinnion (2010) for quantitative summaries of exposure levels and Appendix D in CDC (2015) for LOD values. For additional details, see the CDC (2021a) website.

### 2.4. Xenobiotic classes

The 192 xenobiotics we analyzed were categorized into 21 classes. These included seven polychlorinated dibenzo-p-dioxins (PCDDs), five polychlorinated dibenzofurans (PCDFs), three non-ortho dioxin-like polychlorinated biphenyls (NODL PCBs), six mono-ortho dioxin-like polychlorinated biphenyls (MODL PCBs), 29 non-dioxin-like polychlorinated biphenyls (nonDL PCBs), 14 volatile organic compounds (VOCs), 22 volatile organic compound metabolites (VOC Metabolites), 29 metals and metalloids (Metals), 14 phthalates and phthalate alternative metabolites (Phthalates), 10 polycyclic aromatic hydrocarbon metabolites (PAH Metabolites), 10 organochlorine pesticides and metabolites (Pesticides), two herbicides and metabolites (Herbicides), two fungicides and metabolites (Fungicides), four pyrethroid insecticide metabolites (Pyrethroid Insecticides), six organophosphorus insecticides classified as dialkyl phosphate metabolites (OP/DPM Insecticides), four organophosphorus insecticides classified as specific pesticides and metabolites (OP/Other Insecticides), two insect repellents and metabolites (Insect Repellents), nine perfluoroalkyl and polyfluoroalkyl substances (PFAS), three perchlorate and other anions (Anions), nine personal care and consumer product chemicals and metabolites (Consumer Products), and two tobacco alkaloids and metabolites (Tobacco Biomarkers).

### 2.5. Dioxin-like compounds

We paid special attention to compounds with dioxin-like (DL) activity, which have well-documented immunotoxic effects (Lawrence and Vorderstrasse 2004) and impacts on autoimmunity in animal studies (Boule et al. 2015; Gogal and Holladay 2008; Kummari et al. 2021), as well as associations with autoimmune thyroid disease in human studies (Spaulding 2011). In addition to analyzing individual DL compounds, we predicted results for mixtures of them using toxic equivalency factors (TEFs) assigned by the World Health Organization (Van den Berg et al. 2006); TEFs are relative potency factors, based on expert judgment, for transforming concentrations to a common potency scale relative to 2,3,7,8-tetrachlorodibenzo-p-dioxin (TCDD). Making standard additivity assumptions, we multiplied each DL compound’s concentration by its TEF and then summed those products to create a toxic equivalent (TEQ) concentration for each DL mixture. The DL compounds and their TEFs are listed in Supp. Table 1a.

### 2.6. Covariate adjustments

As in our prior study (Dinse et al. 2016), we adjusted for participant sex, age, race/ethnicity, BMI, PIR, and NHANES cycle. Our current study also adjusted for smoking, birthplace, and elderly status. We treated age as continuous and used categorical variables for sex (male, female), race/ethnicity (non-Hispanic White, non-Hispanic Black, Mexican American, Other), BMI (underweight/healthy, overweight, obese), PIR (below, at or above poverty level), NHANES cycle (1988–1991, 1999–2000, 2001–2002, 2003–2004, 2011–2012), smoking exposure (none, secondhand, active), birthplace (U.S., another country), and elderly status (12–79, ≥80 years old). Smoking categories were based on lab serum cotinine levels of < 0.05 ng/ml (none), 0.05–15 ng/ml (secondhand), and > 15 ng/ml (active); thus, our analyses of cotinine and NNAL (the other Tobacco Biomarker) did not adjust for smoking. NHANES truncated ages above a certain limit, and that limit differed across cycles, so for consistency we truncated all ages at 80 years (the lowest limit) and added the elderly indicator to allow for the mass at age 80 years.

### 2.7. Statistical analysis

Large proportions of nondetects complicate the usual modeling of ANA positivity as a covariate-adjusted function of xenobiotic concentration. As in our earlier analysis (Dinse et al. 2016), we addressed this challenge by reversing roles and treating ANA status as a predictor, xenobiotic concentration as the response, and nondetects as censored concentrations (Dinse et al. 2014). We modeled concentration with a lognormal distribution, a standard choice (Ott 1994), which assumes the natural logarithm of concentration is normally distributed. We modeled the mean of log-concentration as a linear function of ANA status and adjustment covariates; thus, covariate effects on the mean concentration are multiplicative. This is a type of accelerated failure time model often used in survival analysis. We assessed the ANA association with each xenobiotic via the sign (directionality), magnitude, and statistical significance of the estimated ANA regression coefficient. We reported P-values as indications of statistical significance. We did not focus on formal hypotheses, as our analysis was primarily exploratory, but we used two-sided P-values to identify ANA/xenobiotic associations (in either direction) that might merit further consideration.

We used the LIFEREG procedure in SAS (version 9.4, SAS Institute, Cary, NC, USA) to perform lognormal regression analyses, where the response was either an individual xenobiotic concentration or a mixture TEQ concentration. Xenobiotics measured in urine were modeled on a creatinine basis to account for dilution, while concentrations of lipophilic xenobiotics were modeled on a per lipid basis. Our analyses used categorical covariates for all factors except age, which we modeled by a restricted cubic spline (Harrell 2001). We analyzed 12,058 of the 13,519 participants (89%) after excluding 1,461 with missing adjustment covariates (1,171 for PIR, 231 for smoking, 96 for BMI, and 13 for birthplace). Our initial analysis focused on all participants, but we also examined subgroups based on sex, age (12–19, 20–49, ≥50 years), or race/ethnicity (non-Hispanic White, non-Hispanic Black, Mexican American), as well as the six combinations of sex and age group. Analyses that stratified by sex or race/ethnicity did not include a covariate for sex or race/ethnicity, respectively, but age-stratified analyses included a linear age term (which used a single degree of freedom) rather than a cubic spline (which used three degrees of freedom) and the analysis of ≥ 50 year-olds also included the elderly indicator.

### 2.8. Assessing ANA/xenobiotic associations

The ANA regression coefficient gauges the association between ANA positivity and xenobiotic concentration, and its exponentiated value is the ratio of mean concentrations for ANA-positive versus ANA-negative persons (Dinse et al. 2016). A mean concentration ratio (MCR) > 1.0 indicates a positive ANA/xenobiotic association (i.e., persons with higher concentrations of the xenobiotic had a higher proportion who tested positive for ANA), while an MCR < 1.0 indicates a negative association (i.e., persons with higher concentrations had a lower proportion with ANA). The MCR can be viewed as the covariate-adjusted fractional difference between the geometric mean concentrations for people with and without ANA. For example, an MCR of 1.5 indicates that people with ANA have a 50% higher concentration of the xenobiotic, on average, than people without ANA. Thus, MCR estimates enable us to meaningfully compare the strengths of ANA associations across xenobiotics. The logarithmic distance of the MCR from 1.0 reflects the magnitude of an association. Statistical significance is gauged by the P-value from a two-sided test of no ANA/xenobiotic association. Identifying both the most statistically significant associations and those of greatest magnitude can be informative. Focusing only on P-values to assess an association’s statistical significance (regardless of magnitude) or focusing only on MCR estimates to assess an association’s magnitude (regardless of statistical significance) might miss patterns of interest.

We assessed each xenobiotic’s association with ANA in the general population and in subgroups. We estimated the overall ANA/xenobiotic association by fitting the full model to data on all participants (which adjusts for covariate main effects) and also by combining results across sex-by-age strata or race/ethnicity strata (which allows covariate adjustments to vary by strata). This second method derived a weighted average of stratum-specific MCR estimates, using inverse variance estimates as weights; thus, larger samples had greater influence. We also performed separate analyses within sex, age, and race/ethnicity subgroups, which may identify xenobiotics that only correlate with ANA in certain demographic categories. We note that combining results across strata to produce overall assessments of ANA-relevant xenobiotics can help avoid criticisms of bias due to focusing on selective findings from individual subgroups.

### 2.9. Censored concentrations

An individual xenobiotic concentration below the LOD is left censored, but a mixture TEQ concentration is interval censored if any component concentration is below its LOD (Dinse et al. 2016). If a xenobiotic has not been measured, its concentration is uninformatively censored between zero and infinity. This problem can cascade for a mixture if a person has no information on several component xenobiotics. Instead of excluding such people from mixture analyses entirely, we used a wide but finite censoring interval extending from zero to the largest concentration (across all participants) for each unmeasured xenobiotic.

### 2.10. Survey sampling

The NHANES data were obtained from multistage stratified cluster samples (CDC 2022). The LIFEREG procedure does not incorporate information on sampling strata and clusters, so it does not account for sampling-dependent correlation structure when estimating variances, even though it properly estimates the regression coefficients. Therefore, we based our confidence intervals on a jackknife method that provides standard errors appropriate for complex survey data (see the supplement to Dinse et al. 2016). If a stratum contained only one sampling cluster, the variance was inestimable, so we substituted the mean variance based on all multi-cluster strata. We ignored the survey sampling weights to improve efficiency because our analysis conditioned on variables that influenced the sampling (Korn and Graubard 1999).

### 2.11. Multiple comparisons

In addition to reporting uncorrected P-values (P) to help identify ANA/xenobiotic associations for further study, we also corrected for multiple comparisons, separately in each of nine demographic groups (all participants, males, females, 12–19 year-olds, 20–49 year-olds, ≥50 year-olds, non-Hispanic Whites, non-Hispanic Blacks, and Mexican Americans). Specifically, we applied the false discovery rate (FDR) correction of Benjamini and Hochberg (1995), using the MULTTEST procedure in SAS, to obtain FDR-corrected P-values (P_FDR_) and we employed a critical value of 0.1 for declaring statistical significance. Thus, in each group, we used the FDR correction to control for assessing many ANA/xenobiotic associations.

## 3. Results

### 3.1. Participant characteristics

Overall, 13.7% (1,857/13,519) of the NHANES participants were ANA positive, consistent with previous unadjusted ANA prevalence estimates of 14.4% (Dinse et al. 2016), 13.8% (Satoh et al. 2012), 12.9% (Mariz et al. 2011), and 13.3% (Tan et al. 1997). The distributions of these participants by sex, age, race/ethnicity, BMI, PIR, and smoking exposure are given elsewhere (see Table 1 and Supp. Table 1 of Dinse et al. 2020).

### 3.2. Overview of all ANA/xenobiotic associations

**Fig. 1.**
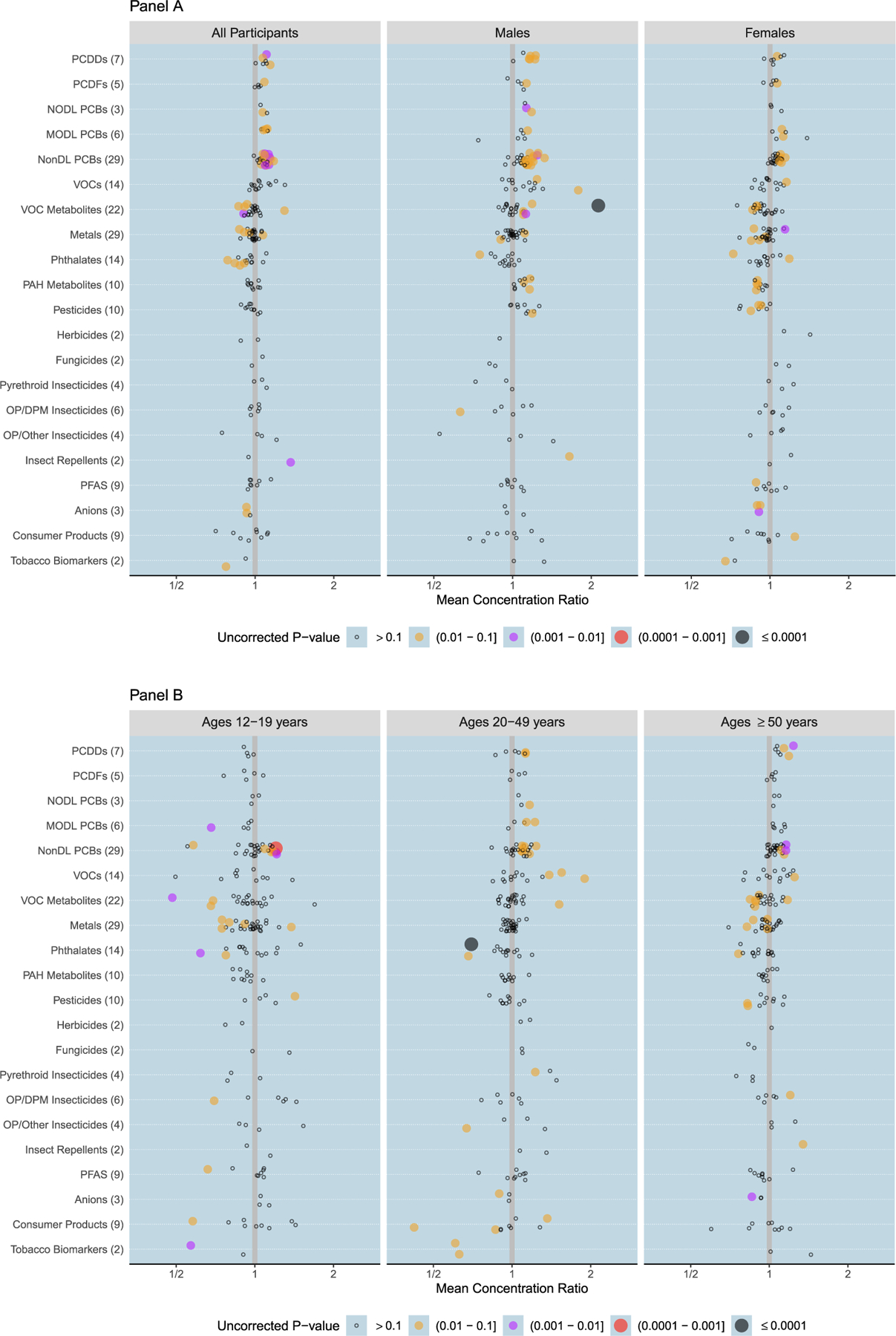

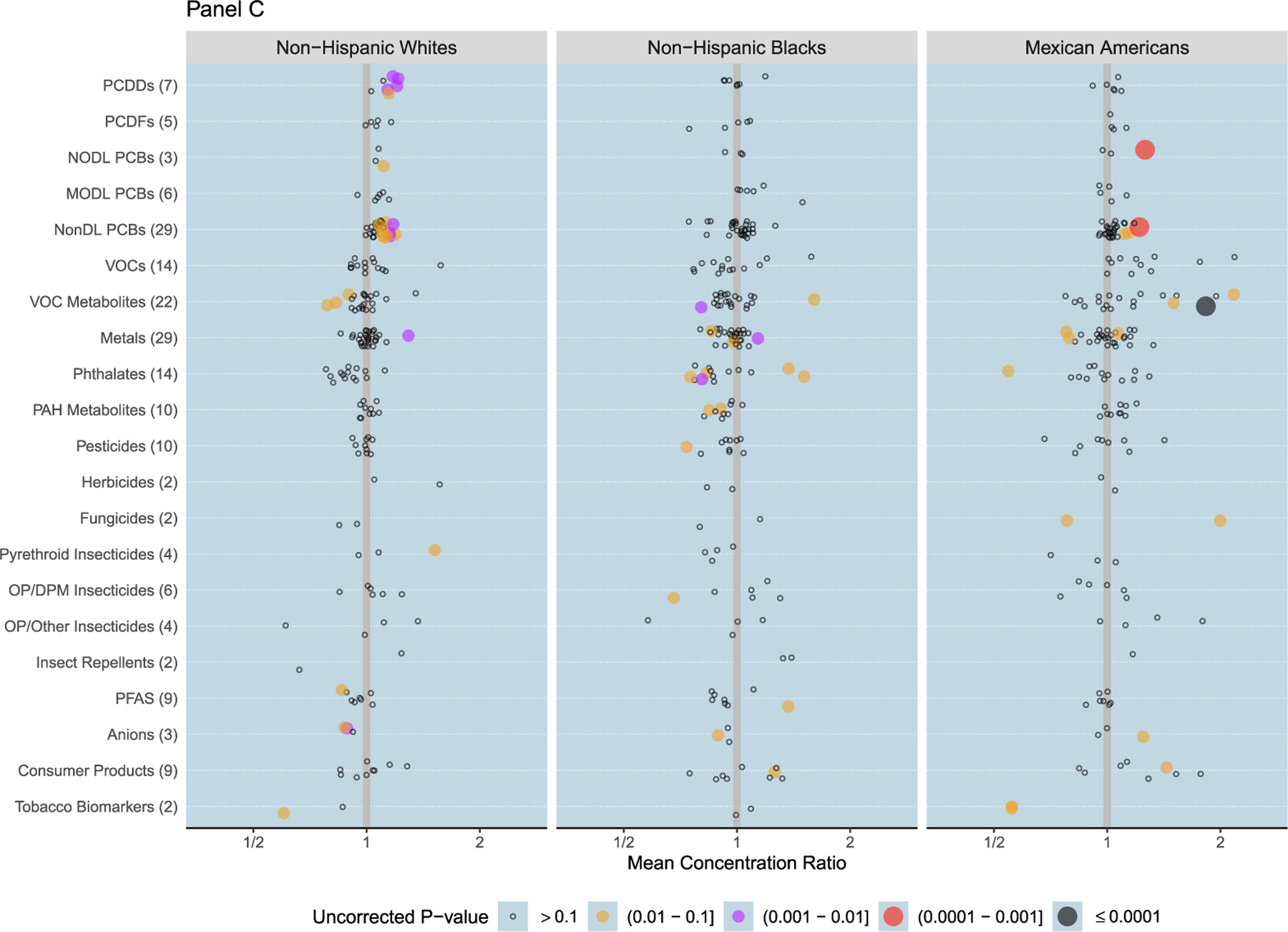
Direction, magnitude, and statistical significance of associations between ANA and 192 xenobiotics. Data are from the years 1988–1991, 1999–2004, and 2011–2012 of the National Health and Nutrition Examination Survey. Xenobiotics are arranged in 21 classes, as described in the Methods section, with the number per class given in parentheses. Results are shown for all participants, males, and females in Panel A; for ages 12–19, 20–49, and ≥ 50 years in Panel B; and for non-Hispanic Whites, non-Hispanic Blacks, and Mexican Americans in Panel C. The plots depict estimates of the mean concentration ratio (MCR) for ANA-positive versus ANA-negative persons. Each symbol identifies a different xenobiotic and its location along the horizontal axis equals the estimated MCR, which gauges the magnitude of the ANA/xenobiotic association. The MCRs were estimated under a lognormal model for xenobiotic concentration that adjusted for ANA, sex, age, elderly status, race/ethnicity, BMI, PIR, smoking, birthplace, and cycle, but not for the sampling weights. MCR values < 1.0 reflect negative associations and those > 1.0 reflect positive associations. Symbol size and color indicate an association’s statistical significance: a large black dot denotes P ≤ 0.0001, a large red dot denotes 0.0001 < P ≤ 0.001, a medium purple dot denotes 0.001 < P ≤ 0.01, a medium orange dot denotes 0.01 < P ≤ 0.1, and a small black circle denotes P > 0.1. The P-values are not corrected for multiple comparisons and are from two-sided tests of no ANA/xenobiotic association.

Summaries of all ANA/xenobiotic associations, overall and in subgroups, are depicted in Fig. 1. The bubble plots show the direction, magnitude, and uncorrected statistical significance of the ANA association with each xenobiotic (arranged by class). The three most statistically significant findings, denoted by large black dots, were positive ANA associations with N-acetyl-S-(2-hydroxy-3-butenyl)-L-cysteine (MHB2) in males (P = 2.6×10^−7^) and with N-acetyl-S-(phenyl)-L-cysteine (PMA) in Mexican Americans (P = 4.5×10^−5^), and a negative ANA association with mono-benzyl phthalate (MBzP) in 20–49 year-olds (P = 4.6×10^−5^). All three remained significant after correcting for multiple comparisons, with P_FDR_ values of 0.000048, 0.0080, and 0.0086, respectively. Table 1 lists overall associations for which P < 0.005 (a conservative limit based on one-tenth the usual 0.05 cutoff) and provides the xenobiotic name, class name, and three sets of MCR estimates, 95% CIs, and uncorrected P-values. The three sets of results were derived from analyses that: (a) did not stratify; (b) combined across sex-by-age strata; and (c) combined across race/ethnicity strata. Table 2 lists subgroup associations for which P < 0.005 and provides the xenobiotic name, class name, demographic group, MCR estimate, 95% CI, and uncorrected P-value, plus information to help assess data adequacy in the subgroups. The P-values for the two results in Table 1 and nine results in Table 2 that remained statistically significant at the 0.1 level after FDR correction are bolded and footnoted.

The ANA/xenobiotic associations of greatest magnitude in Fig. 1 are those farthest from the reference line. Ignoring statistical significance, the largest positive association was with ethylbenzene in Mexican Americans (MCR = 3.50) and the largest negative association was with alachlor mercapturate in males (MCR = 0.25). Restricting attention to overall (non-subgroup) results, 3-diethylcarbamoylbenzoic acid (DEET acid) had the largest positive ANA association (MCR = 1.37) and butyl paraben had the largest negative ANA association (MCR = 0.71). The set of xenobiotics with the largest associations in either direction, based on symmetric thresholds of MCR > 3/2 (positive) and MCR < 2/3 (negative), are listed in Tables 3 and 4, respectively.

The following subsections explore xenobiotic classes, and the xenobiotics within them, with respect to both the statistical significance and magnitude of observed ANA associations.

### 3.3. Dioxin-like compounds

Only 21 of the 26 DL xenobiotics had sufficient data for our analysis: seven PCDDs, five PCDFs, three NODL PCBs, and six MODL PCBs (Supp. Table 1a). In addition to estimating ANA associations with individual DL xenobiotics, we also predicted associations with TEF-based mixtures of xenobiotics within each DL class, as well as across all DL classes (Supp. Tables 2a, 3a, and 4a). The DL xenobiotics were only measured in cycles 2–4 and, for reasons described previously (Dinse et al. 2016), our mixture analyses excluded one PCDD and one MODL PCB because they had data in only one or two of those cycles.

In the full sample, all MCR estimates equaled or exceeded 1.0, suggesting positive ANA associations with DL xenobiotics and their mixtures (Supp. Table 2a). This consistency in direction is noteworthy, but its relevance may depend on the degree to which these compounds are correlated, and an extensive correlation analysis is beyond the scope of our study. The positive ANA association with 1,2,3,4,6,7,8,9-octachlor-odibenzo-p-dioxin (1,2,3,4,6,7,8,9-OCDD) was the most statistically significant (P = 0.007), and many overall ANA associations with DL xenobiotics or their mixtures had uncorrected P-values below the conventional 0.05 cutoff (Supp. Table 2a), but none remained statistically significant after correcting for multiple comparisons. With respect to magnitude, the positive ANA association with 1,2,3,7,8,9-hexachlorodibenzo-p-dioxin (1,2,3,7,8,9-HxCDD) was largest (MCR = 1.14).

Some subgroup analyses also suggested positive ANA associations with DL xenobiotics and their mixtures, especially in males (Supp. Table 2a) and non-Hispanic Whites (Supp. Table 4a). Nevertheless, only the positive ANA association with 3,4,4′,5-tetrachlorobiphenyl (PCB 81) in Mexican Americans (MCR = 1.26; CI = 1.12,1.42; P_FDR_ = 0.013) remained statistically significant after correcting for multiple comparisons (Tables 2 and 5). When combining results across race/ethnicity subgroups rather than directly analyzing the full sample (Table 1), the overall ANA association with PCB 81 was significant after correction (MCR = 1.20; CI = 1.08, 1.33; P_FDR_ = 0.057), which may have been driven by the association in Mexican Americans.

### 3.4. Non-dioxin-like polychlorinated biphenyls

In the full sample, all except one of the 28 nonDL PCBs with adequate data were positively associated with ANA, though the relevance of this consistency may depend on how correlated these compounds are. The positive ANA association with 2,2′,4,5′-tetrachlorobiphenyl (PCB 49) was the most significant (P = 1.2×10^−3^), and 11 other nonDL PCBs also had uncorrected P-values below 0.05, but none remained statistically significant after correcting for multiple comparisons in the full-sample analysis (Table 1).

Among the demographic subgroups, eight ANA associations with nonDL PCBs in males, five in females, six in 12–19 year-olds, one in 20–49 year-olds, four in ≥ 50 year-olds, six in non-Hispanic Whites, and one in Mexican Americans had uncorrected P-values below 0.05 (Supp. Tables 2b, 3b, and 4b). Of those, only the ANA association with 2,2′,3,3′,4,5,5′-heptachlorobiphenyl (PCB 172) in the 12–19 age group was negative. After correcting for multiple comparisons, the four findings that remained statistically significant (Tables 2 and 5) were the positive ANA associations in the 12–19 age group for 2,2′,3,5′-tetrachlorobiphenyl (PCB 44) (MCR = 1.21; CI = 1.07,1.36; P_FDR_ = 0.081), PCB 49 (MCR = 1.20; CI = 1.08,1.34; P_FDR_ = 0.081), and 2,2′,3,4′,5′,6-hexachlorobiphenyl (PCB 149) (MCR = 1.21; CI = 1.08,1.36; P_FDR_ = 0.081), and in Mexican Americans for 2,2′,3,3′,4,4′,5,5′,6-nonachlorobiphenyl (PCB 206) (MCR = 1.22; CI = 1.09,1.36; P_FDR_ = 0.030).

### 3.5. Volatile organic compounds and metabolites

Only 14 of the 43 VOCs had adequate data for analysis. Of those, none appeared associated with ANA overall, and while four showed weak evidence (P < 0.05) of a positive ANA association in subgroups (Supp. Tables 2c, 3c, and 4c), none remained statistically significant after correcting for multiple comparisons. Ignoring statistical significance, nine associations were relatively large in magnitude (Tables 2 and 3), especially the positive ANA association with ethylbenzene (MCR = 3.50) in Mexican Americans.

Of the 27 VOC metabolites, 22 had adequate data, and 13 showed at least mild evidence of an association with ANA (Supp. Tables 2d, 3d, and 4d). However, the only two results that remained statistically significant after correcting for multiple comparisons were the positive ANA associations with MHB2 in males (MCR = 2.12; CI = 1.59,2.82; P_FDR_ = 4.8×10^−5^) and with PMA in Mexican Americans (MCR = 1.82; CI = 1.37,2.43; P_FDR_ = 0.0080), and both had fairly large MCR estimates (Tables 2 and 5).

### 3.6. Metals

Cadmium, lead, manganese, mercury, and selenium were measured in two of the three matrices (blood, serum, urine). Of the 33 metal/matrix combinations, 29 had adequate data, with MCR estimates ranging from 0.75 to 1.38 (Supp. Tables 2e, 3e, and 4e). Though several metals showed some evidence of an association with ANA (P < 0.05), none remained significant after correcting for multiple comparisons.

### 3.7. Phthalates

Of the 14 phthalates, none were associated with ANA in the full sample and in most subgroups, though two showed mild evidence of a positive association in non-Hispanic Blacks (Supp. Tables 2f, 3f, and 4f). However, ANA was significantly negatively associated with MBzP in 20–49 year-olds (MCR = 0.70; CI = 0.59,0.83; P_FDR_ = 0.0086) and with mono-n-butyl phthalate (MnBP) in 12–19 year-olds (MCR = 0.62; CI = 0.46,0.84; P_FDR_ = 0.081) (Tables 2 and 5).

### 3.8. Polycyclic aromatic hydrocarbons

None of the 10 PAH metabolites showed an overall association with ANA, though two showed weak evidence (P < 0.05) of an ANA association in certain subgroups (Supp. Tables 2f, 3f, and 4f). The MCR estimates ranged from 0.82 to 1.20, but none differed significantly from 1.0 after correcting for multiple comparisons.

### 3.9. Pesticides, herbicides, fungicides, and insecticides

Ten of the 15 organochlorine pesticides (but neither carbamate pesticide) had adequate data. Only the positive ANA association with *trans*-nonachlor in males and the negative ANA associations with *trans*-nonachlor in females, with p,p’-dichlorodiphenyltrichloroethylene (p, p’-DDE) in ≥ 50 year-olds, and with beta-hexachlorocyclohexane in non-Hispanic Blacks had an uncorrected P < 0.05, but none of these associations were statistically significant after correcting for multiple comparisons. The MCR estimates for the 10 pesticides ranged from 0.68 to 1.42 (Supp. Tables 2g, 3g, and 4g).

Only two of the six herbicides had adequate data for analysis, and neither was associated with ANA (Supp. Tables 2h, 3h, and 4h). Similarly, four of the six organophosphorus insecticides in the specific pesticides and metabolites class had adequate data and none were associated with ANA. Both fungicides had adequate data and *ortho*-phenylphenol showed weak evidence of a negative ANA association in Mexican Americans, but not after correcting for multiple comparisons. Three of the five pyrethroid insecticide metabolites had adequate data and *cis*-3-(2,2-dichlorovinyl)-2,2-dimethyl cyclopropane carboxylic acid (*cis*-DCCA) showed weak evidence of a positive ANA association in non-Hispanic Whites, but not after correcting for multiple comparisons. All six organophosphorus insecticides in the dialkyl phosphate metabolites class had adequate data, but only diethylthiophosphate (DETP) in non-Hispanic Blacks showed an association (negative) with ANA, which was not statistically significant after correcting for multiple comparisons. Finally, two of the three insect repellents had adequate data, but only DEET acid showed evidence of an ANA association (P < 0.05), which was positive both overall and in males, but it did not remain statistically significant after correcting for multiple comparisons. Though not statistically significant, several of these xenobiotics had large positive MCR estimates (Table 3), especially pentachlorophenol in Mexican Americans (MCR = 1.99), and several had large negative MCR estimates (Table 4), most notably alachlor mercapturate in males (MCR = 0.25).

### 3.10. Perfluoroalkyl and polyfluoroalkyl substances

Nine of the 12 PFAS had adequate data in some groups, with MCR estimates ranging from 0.66 to 1.37 (Supp. Tables 2i, 3i, and 4i). Only two PFAS had an uncorrected P < 0.05, either overall or in a subgroup, and none of these ANA associations remained statistically significant after correcting for multiple comparisons.

### 3.11. Anions

All three anions had adequate data for analysis, with MCR estimates ranging from 0.86 to 1.25 (Supp. Tables 2i, 3i, and 4i). Both nitrate and perchlorate had an uncorrected P < 0.05, overall and in several subgroups, but none of these ANA associations were statistically significant after correcting for multiple comparisons.

### 3.12. Consumer products

All nine personal care and consumer product chemicals had adequate data, with a wide range of MCR estimates from 0.42 to 1.77 (Supp. Tables 2i, 3i, and 4i). Although three had an uncorrected P < 0.05, either overall or in a subgroup, none of these ANA associations remained statistically significant after correcting for multiple comparisons.

### 3.13. Tobacco biomarkers

Both tobacco biomarkers had adequate data, with MCR estimates ranging from 0.56 to 1.09 for cotinine and from 0.56 to 1.44 for NNAL (Supp. Tables 2i, 3i, and 4i). Though not statistically significant after correcting for multiple comparisons, we observed some evidence (P < 0.05) of a negative ANA association with cotinine in all participants (MCR = 0.77), in females (MCR = 0.68), in 12–19 year-olds (MCR = 0.57), in 20–49 year-olds (MCR = 0.63), in non-Hispanic Whites (MCR = 0.60), and in Mexican Americans (MCR = 0.56).

### 3.14. Additional analyses

Beyond the metals examined in our initial analyses, we also investigated iron, which can be viewed as a xenobiotic as well as an essential nutrient. We found no overall association between ANA and iron in the full sample and only weak evidence of a negative association in females (MCR = 0.96; CI = 0.93,1.00; P = 0.038), in ≥ 50 year-olds (MCR = 0.96; CI = 0.93,0.99; P = 0.018), and in non-Hispanic Blacks (MCR = 0.94; CI = 0.89,1.00; P = 0.043), none of which were statistically significant after correcting for multiple comparisons.

Our initial analyses focused on the full sample and on subgroups defined by sex, age, or race/ethnicity, but we also considered two-way combinations of these three demographic factors. Results stratified by sex and age are shown in Supp. Table 5 (three age groups for males) and Supp. Table 6 (three age groups for females). However, we do not provide results stratified by sex and race/ethnicity or by age and race/ethnicity because data in some strata were too sparse to reliably interpret. In the sex-by-age analysis, only two ANA/xenobiotic associations (both with metals) were statistically significant after correcting for multiple comparisons. We found a positive ANA association with molybdenum in ≥ 50 year-old females (MCR = 1.21; CI = 1.10,1.33; P_FDR_ = 0.029) and a negative ANA association with tin in 12–19 year-old females (MCR = 0.45; CI = 0.30,0.68; P_FDR_ = 0.022). When sex and age were considered individually, there was some evidence of an ANA association with molybdenum for females but little for ≥ 50 year-olds, and only weak evidence of an ANA association with tin for 12–19 year-olds and none for females (Supp. Tables 2e and 3e).

### 3.15. Summary of overall results

When simultaneously analyzing all participants (i.e., the full sample), uncorrected P-values suggested several xenobiotics might be associated with ANA, but none of these associations remained statistically significant after correcting for multiple comparisons (Table 1). Alternative assessments based on combining subgroup results did not indicate overall ANA associations with any xenobiotics when averaging across sex-by-age strata, but they did for race/ethnicity strata. Combining stratum-specific MCR estimates across the three race/ethnicity groups suggested a positive overall association with PCB 81 (MCR = 1.20; CI = 1.08,1.33; P_FDR_ = 0.057) and a negative overall association with N-acetyl-S-(2-hydroxyethyl)-L-cysteine (HEMA) (MCR = 0.82; CI = 0.74,0.92; P_FDR_ = 0.057). Ignoring statistical significance, ANA had the greatest positive overall association with DEET acid (MCR = 1.37; CI = 1.13,1.66) and the greatest negative overall association with butyl paraben (MCR = 0.71; CI = 0.42,1.20).

### 3.16. Summary of subgroup results

Within single-factor demographic subgroups, ANA associations with nine xenobiotics remained statistically significant after correcting for multiple comparisons (Tables 2 and 5). Ordered by FDR-corrected P-value, ANA appeared positively associated with MHB2 in males (MCR = 2.12; CI = 1.59,2.82; P_FDR_ = 4.8×10^−5^), PMA in Mexican Americans (MCR = 1.82; CI = 1.37,2.43; P_FDR_ = 0.0080), PCB 81 in Mexican Americans (MCR = 1.26; CI = 1.12,1.42; P_FDR_ = 0.013), PCB 206 in Mexican Americans (MCR = 1.22; CI = 1.09,1.36; P_FDR_ = 0.030), PCB 44 in 12–19 year-olds (MCR = 1.21; CI = 1.07,1.36; P_FDR_ = 0.081), PCB 49 in 12–19 year-olds (MCR = 1.20; CI = 1.08,1.34; P_FDR_ = 0.081), and PCB 149 in 12–19 year-olds (MCR = 1.21; CI = 1.08,1.36; P_FDR_ = 0.081), while ANA appeared negatively associated with MBzP in 20–49 year-olds (MCR = 0.70; CI = 0.59,0.83; P_FDR_ = 0.0086) and MnBP in 12–19 year-olds (MCR = 0.62; CI = 0.46,0.84; P_FDR_ = 0.081). Focusing only on magnitude, ANA was most positively associated with ethylbenzene in Mexican Americans (MCR = 3.50; CI = 0.48,25.54) and most negatively associated with alachlor mercapturate in males (MCR = 0.25; CI = 0.04,1.74). Considering both statistical significance and effect size, the positive ANA/MHB2 association in males and the negative ANA/MnBP association in 12–19 year-olds were the most extreme in magnitude while remaining statistically significant after correcting for multiple comparisons.

### 3.17. Exposure sources and toxicologic associations

Possible exposure sources for, and toxicologic associations with, the xenobiotics having ANA associations of greatest statistical significance after correcting for multiple comparisons (P_FDR_ < 0.1) are listed in Table 5.

## 4. Discussion

To our knowledge, this is the largest and most comprehensive study to date of associations between ANA and xenobiotic exposures. The observed ANA/xenobiotic associations varied in direction, magnitude, and statistical significance. Though some observed associations may be due to sampling variation alone, others may reflect true associations. Also, members of a few classes of xenobiotics showed consistently positive (or negative) associations with ANA, even though not all were statistically significant. Some associations observed in the full sample were reinforced by similar findings in multiple subgroups, whereas other overall associations appeared driven by a particular demographic.

In the full sample, xenobiotics in several classes consistently had ANA associations in the same direction; correlations among these xenobiotics could be relevant, but an extensive correlation analysis was beyond our scope. Overall, DL compounds were positively associated with ANA, as were the PCDD, PCDF, NODL PCB, and MODL PCB classes (and the various TEF mixtures). Similarly, ANA associations with nonDL PCBs also were consistently positive. In addition to the consistency in direction, many of these positive ANA associations initially appeared statistically significant (P < 0.05), though few remained significant after correcting for multiple comparisons. In contrast, some classes suggested mainly negative associations with ANA, most notably phthalates. Other classes were either too small to draw general conclusions about overall ANA associations or else exhibited a mix of positive and negative associations, most of which were not statistically significant after correcting for multiple comparisons.

Many subgroup analyses supported the class-specific results based on all participants, though the smaller sample sizes often led to weaker statistical significances. The consistently positive ANA associations estimated for DL compounds and nonDL PCBs were mirrored in both sexes (especially males), adults (ages ≥ 20 years), and non-Hispanic Whites. Likewise, the generally negative ANA associations with phthalates overall were also observed in several subgroups. Occasionally, however, subgroup analyses led to findings that differed from those in the full sample. For example, ANA appeared to be negatively associated with PAH metabolites in females and non-Hispanic Blacks but showed little evidence of an association in either direction for all participants combined or within the other subgroups.

Several individual xenobiotics had noteworthy ANA associations. The most statistically significant finding was a positive association between MHB2 and ANA in males, which remained highly significant after correcting for multiple comparisons (P_FDR_ = 4.8 × 10^−5^). The estimated MCR was 2.12 (CI = 1.59,2.82), suggesting that the average concentration of MHB2 in men with ANA was more than twice that in men without ANA. Though statistical significances were less, estimated ANA associations with MHB2 also were positive in the full sample and in several other subgroups. The MCR estimates ranged from 0.75 in females to 2.12 in males, suggesting a possible sex effect with higher exposures in males, which is consistent with the probable sources (Table 5). The next most significant finding was a positive ANA association with PMA in Mexican Americans, which also remained significant after correcting for multiple comparisons (P_FDR_ = 0.0080). The estimated MCR was 1.82 (CI = 1.37,2.43), suggesting that the average concentration of PMA in Mexican Americans with ANA was nearly twice that in those without ANA. Both of these xenobiotics are VOC metabolites; MHB2 is a metabolite of 1,3-butadiene and PMA is a known irritant that is a metabolite of benzene (NCBI 2022a,b). Benzene is a carcinogen which has many toxicities, including immunotoxicity, and could alter immune regulation as seen in autoimmunity, but no studies have specifically evaluated its possible association with autoimmune diseases (NTP 2021).

Of the other findings that remained statistically significant after correcting for multiple comparisons (P_FDR_ < 0.1), five involved PCBs. Specifically, PCB 44, PCB 49, and PCB 149 were positively associated with ANA in 12–19 year-olds, while PCB 81 and PCB 206 were positively associated with ANA in Mexican Americans. Ignoring statistical significance, nearly all ANA associations with these five PCBs were positive, both in the full sample and in subgroups. There are limited data regarding the immune impacts of specific PCBs, but most studies suggest immunosuppressive effects or disruptions in immune homeostasis, which may allow for increased or more persistent infections or other immune stimulatory processes that may relate to these positive ANA associations (Pollard et al. 2010).

The most statistically significant negative associations were between ANA and two metabolites of phthalates, MBzP in 20–49 year-olds and MnBP in 12–19 year-olds, both of which remained significant after correcting for multiple comparisons (P_FDR_ = 0.0086 for MBzP and P_FDR_ = 0.081 for MnBP). The estimated MCR for MBzP was 0.70 (CI = 0.59,0.83), suggesting that the average concentration of MBzP in 20–49 year-olds with ANA was only about two-thirds of that in those without ANA. Though statistical significances were less, ANA associations with MBzP were consistently negative in the full sample and all but one subgroup, with MCR estimates ranging from 0.70 to 1.02. Similarly, all ANA associations with the related chemical MnBP were negative, with MCR estimates ranging from 0.62 to 0.92; the most statistically significant negative ANA association with MnBP was in 12–19 year-olds (MCR = 0.62; CI = 0.46,0.84; P_FDR_ = 0.081). Both MBzP and MnBP are metabolites of phthalates used in the manufacture of polyvinyl chloride plastics and belong to a group of endocrine-disrupting chemicals suspected of having numerous impacts on human health, including influences on the immune system (Table 5). Although only a few studies have specifically assessed their immune roles, phthalates have been found to have variable impacts on immune or inflammatory responses in both epidemiologic and animal studies, ranging from stimulatory activity to the absence of any effect, to inhibitory or immunosuppressive activity (Kimber et al., 2010). Hansen et al. (2015) showed that, *in vitro*, MnBP enhanced the secretion of interleukin (IL)-6, IL-10, chemokine CXCL8, and tumor necrosis factor (TNF)-α by stimulated monocytes/macrophages, as well as the secretion of IL-6 by stimulated T cells. Also, Wang et al. (2020) reported that MBzP increased the expression of TNF-α, monocyte chemoattractant protein-1 (MCP-1), and cell surface cluster of differentiation antigen 68 (CD68) in placentae of male, but not female, fetuses. In addition, MBzP was positively associated with current allergic symptoms and sensitization in adults in a prior NHANES study (Hoppin et al. 2013). While it remains unclear how these effects and associations might relate to protection from development of ANA in younger Americans, they clearly show an immune influence.

Though associations may appear interesting due to their statistical significance or the size of their MCR estimate, some are based on sparse data, especially in the subgroups, and should be interpreted cautiously. Tables 2–4 list four factors to help assess reliability in the subgroups: percentage of participants with a concentration below the limit of detection (%BLOD); number of ANA-positive participants with a detectable concentration (#ANA+); number of strata with a singleton cluster (#SING); and number of participants analyzed (#ANAL). Ideally, %BLOD and #SING should be small, while #ANA+ and #ANAL should be large. For example, regarding two of the most statistically significant findings, all four factors appear reasonable for the negative ANA association with MBzP in 20–49 year-olds, but the somewhat low #ANA+ for the positive ANA association with PMA in Mexican Americans may merit cautious interpretation (Table 2).

Despite evidence of ANA/xenobiotic associations in several subgroups, the same was not necessarily true for the population as a whole. After correcting for multiple comparisons, none of the xenobiotics were statistically significantly associated with ANA when analyzing the full sample directly, though two showed evidence of an overall ANA association when stratifying by race/ethnicity and then combining results across those strata (Table 1). The latter analysis allowed adjustment covariates to vary with race/ethnicity and suggested a positive overall ANA association with PCB 81 (MCR = 1.20; CI = 1.08,1.33; P_FDR_ = 0.057) and a negative overall ANA association with HEMA (MCR = 0.82; CI = 0.74,0.92; P_FDR_ = 0.057).

One strength of the present study is its large size. Compared with our earlier study of ANA/xenobiotic associations (Dinse et al. 2016), which involved data on 109 xenobiotics from 4,754 NHANES participants, the current study involved data on more than twice as many xenobiotics (253) from nearly three times as many participants (13,519). After excluding xenobiotics with inadequate data and participants with missing covariates, our earlier study ultimately analyzed data on 87 xenobiotics from 4,340 participants, while our present study analyzed data on 192 xenobiotics from 12,058 participants. Another strength is that the current study used an ANA assay that is more standard in clinical laboratories, whereas the earlier study used an ANA assay with more sensitive secondary antibodies, deployed mainly in research settings. Rather than combining old assay results, obtained a decade earlier from the original NHANES cycles (1999–2004), with new assay results from the additional cycles (1988–1991, 2011–2012), we applied the new assay to biospecimens from all five cycles so that all results were determined in the same way and at the same time.

The current study had several other strengths. As in our earlier study, exposures were objectively measured (in serum, whole blood, or urine samples) rather than being self-reported (in surveys). Compared to conventional analyses that substitute specific values (e.g., LOD/2 or $\text{LOD}/\sqrt{2}$) for nondetects, thereby treating unknown values as known, our methods considered concentrations below the LOD to be left-censored, which avoids biases and underestimates of variability that are common in traditional analyses. Also, rather than analyzing detect/nondetect dichotomies or discarding nondetects altogether, our method made full use of the available xenobiotic concentration data. Finally, some detection limits change over time, which can adversely affect conventional analyses that use substitution or that focus on the proportion of concentrations above the LOD, whereas our censored-data approach avoids this problem.

As for limitations or areas of concern, one striking inconsistency between our two studies relates to triclosan. The most statistically significant finding in the earlier study was a positive ANA association with triclosan in males, which was the only result that remained significant after correcting for multiple comparisons; no similar association was observed in females, and analyses stratified by age or race/ethnicity were not performed (Dinse et al. 2016). In contrast, the current analyses indicated no significant ANA/triclosan association in both sexes, all three race/ethnicity groups, two of the three age groups, and overall; the only weakly significant finding was a negative association in 12–19 year-olds, which was not significant after correcting for multiple comparisons. Observing a weakly significant association in one study but nothing in the other is not surprising, given the many analyses performed, but the disappearance of a highly significant association merits attention and there may be several contributing factors. The earlier triclosan analysis involved half as many participants as the current analysis, with data from only one cycle versus two cycles. The two studies used secondary antibodies with different sensitivities, performed more than a decade apart. Perhaps the ANA associated with triclosan in males are present at lower titers and were only detected by the earlier study’s more sensitive assay. Whatever the reason for this discrepancy, we consider the current study’s assay to be more appropriate and its larger sample size should confer greater credibility.

Other actual or potential limitations were detailed earlier (Dinse et al. 2016) but are briefly summarized here. Xenobiotic concentrations and ANA were assessed cross-sectionally at only one point in time, so measured exposures may poorly reflect former levels of non-persistent xenobiotics at the time ANA most likely developed. This may relate to our finding that many of the positive associations with ANA were with lipid-soluble, serum/plasma-based biomarkers like PCBs with long half-lives rather than toxicants with shorter half-lives whose effects may be more episodic in terms of exposure, such as the phthalates that were negatively associated with ANA. Many xenobiotics were assessed in only some of the NHANES cycles with ANA data, which reduced the statistical power to detect ANA/xenobiotic associations compared with xenobiotics assessed in all five cycles. Some NHANES xenobiotics might have been of interest but were not assessed in any cycles with ANA data. Model misspecification with respect to confounders could potentially produce underestimates or overestimates of ANA associations with xenobiotic exposures, though we attempted to minimize this problem by adjusting for suspected determinants of exposure or ANA. Finally, the associations we reported, even if confirmed, may not be causal; there could be unmeasured confounding if sources of an exposure are causally related to development of (or protection from) ANA, but not through the associated exposure. In fact, there could be reverse-causal effects if immune-system changes associated with ANA influence either a person’s behavior or the metabolism of certain studied xenobiotics.

In conclusion, this large exploratory study identified potential associations, both positive and negative, between ANA and various xenobiotics. Some associations were seen in the full sample and several subgroups, while others were only apparent in a certain demographic. Further investigation is needed to determine which associations can be confirmed, which groups are at greatest (or least) risk for autoimmunity, and which mechanisms are involved.

## Supplementary Material

MMC4

MMC3

MMC6

MMC2

MMC5

MMC1

## Figures and Tables

**Table 1:** Xenobiotics with overall ANA associations of greatest statistical significance (uncorrected P-value < 0.005), either in the full sample or after combining across sex-by-age or race/ethniciy strata.

				Analysis Combining Subgroup Results across:			
		Analysis of Full Sample		Sex-by-Age Strata		Race/Ethnicity Strata	
Xenobiotic Class	Xenobiotic Name [Alternative/Abbreviated Name]	MCR (95% CI)^a^	P-value^b^	MCR (95% CI)^a^	P-value^b^	MCR (95% CI)^a^	P-value^b^
PCDDs	1,2,3,7,8,9-Hexachlorodibenzo-p-dioxin [1,2,3,7,8,9-HxCDD]	1.14 (1.02, 1.28)	0.023	1.17 (1.05, 1.31)	0.0044	1.16 (1.05, 1.29)	0.0048
NODL PCBs	3,4,4′,5-Tetrachlorobiphenyl [PCB 81]	1.11 (0.97, 1.27)	0.116	1.12 (0.99, 1.27)	0.074	1.20 (1.08, 1.33)	**0.00060** ^c^
nonDL PCBs	2,2′,3,5′-Tetrachlorobiphenyl [PCB 44]	1.13 (1.05, 1.21)	0.0017	1.11 (1.04, 1.19)	0.0030	1.12 (1.04, 1.21)	0.0028
nonDL PCBs	2,2^’^,4,5^’^-Tetrachlorobiphenyl [PCB 49]	1.13 (1.05, 1.22)	0.0012	1.13 (1.05, 1.22)	0.0020	1.13 (1.04, 1.22)	0.0022
nonDL PCBs	2,2′,3,3′,4,5,5′,6-Octachlorobiphenyl [PCB 199]	1.14 (1.05, 1.24)	0.0024	1.11 (1.01, 1.23)	0.030	1.12 (1.02, 1.22)	0.020
nonDL PCBs	2,2′,3,3′,4,4′,5,5′,6-Nonachlorobiphenyl [PCB 206]	1.09 (1.01, 1.18)	0.022	1.05 (0.97, 1.13)	0.238	1.11 (1.04, 1.19)	0.0035
VOC Metabolites	N-Acetyl-S-(2-hydroxyethyl)-L-cysteine [HEMA]	0.86 (0.75, 1.00)	0.051	0.88 (0.76, 1.02)	0.080	0.82 (0.74, 0.92)	**0.00054** ^c^
Metals	Cadmium, urinary	1.07 (1.00, 1.15)	0.043	1.09 (1.02, 1.15)	0.0078	1.10 (1.03, 1.17)	0.0049
Phthalates	Mono-benzyl phthalate [MBzP]	0.88 (0.76, 1.01)	0.072	0.84 (0.72, 0.98)	0.023	0.84 (0.75, 0.95)	0.0040
Insect Repellents	3-Diethylcarbamoylbenzoic acid [DCBA or DEET acid]	1.37 (1.13, 1.66)	0.0016	1.28 (1.01, 1.64)	0.042	1.28 (0.97, 1.70)	0.080
Anions	Nitrate	0.93 (0.87, 0.99)	0.015	0.93 (0.88, 1.00)	0.042	0.91 (0.86, 0.97)	0.0033
Tobacco Biomarkers^d^	Cotinine	0.77 (0.60, 1.00)	0.047	0.71 (0.58, 0.88)	0.0013	0.74 (0.56, 0.98)	0.034

Abbreviations: ANA = antinuclear antibodies; CI = confidence interval; MCR = mean concentration ratio; NODL = non-ortho dioxin-like; nonDL = non-dioxin-like; PCB = polychlorinated biphenyl; PCDD = polychlorinated dibenzo-p-dioxin; VOC = volatile organic compound.

a The MCR is the ratio of mean concentrations for ANA-positive versus ANA-negative participants under a lognormal model for xenobiotic concentration; it is adjusted for sex, age, elderly status, race/ethnicity, BMI, PIR, smoking, birthplace, and cycle, but not for the sampling weights. The null MCR value is 1; MCR > 1 indicates a positive association between ANA and xenobiotic concentration; and MCR < 1 indicates a negative association between ANA and xenobiotic concentration. The CIs are based on variance estimates that make jackknife adjustments for correlations induced by the sampling strata and clusters. When sex-by-age or race/ethnicity stratum-specific MCR values are combined to produce a weighted average MCR value, the weights are proportional to the inverse variance estimates.

b The P-values are displayed to three decimal places (or two significant digits if below 0.100), are two-sided, and are not corrected for multiple comparisons.

c The bolded P-values are statistically significant at the 0.1 level after applying the FDR correction for multiple comparisons.

d The smoking covariate was not included in the model for tobacco biomarkers.

**Table 2:** Xenobiotics with ANA associations of greatest statistical significance (uncorrected P-value < 0.005) within demographic groups defined by sex, age, or race/ethnicity.

Xenobiotic Class	Xenobiotic Name [Alternative/Abbreviated Name]	Demographic Group	%BLOD	#ANA+	#SING	#ANAL	MCR (95% CI)^a^	P-value^b^
PCDDs	1,2,3,4,7,8-Hexachlorodibenzo-p-dioxin [1,2,3,4,7,8-HxCDD]	Non-Hispanic Whites	67	78	1	1,372	1.20 (1.07, 1.35)	0.0016
PCDDs	1,2,3,7,8,9-Hexachlorodibenzo-p-dioxin [1,2,3,7,8,9-HxCDD]	Ages ≥ 50 years	55	148	0	1,446	1.24 (1.08, 1.42)	0.0023
PCDDs	1,2,3,4,6,7,8,9-Hexachlorodibenzo-p-dioxin [1,2,3,4,6,7,8,9-OCDD]	Non-Hispanic Whites	19	212	2	1,877	1.17 (1.05, 1.31)	0.0039
NODL PCBs	3,4,4′,5-Tetrachlorobiphenyl [PCB 81]	Mexican Americans	81	38	12	1,040	1.26 (1.12, 1.42)	**0.00014** ^c^
nonDL PCBs	2,4,4′-Trichlorobiphenyl [PCB 28]	Ages ≥ 50 years	44	113	0	916	1.16 (1.06, 1.27)	0.0015
nonDL PCBs	2,2′,3,5′-Tetrachlorobiphenyl [PCB 44]	Ages 12–19 years	1	59	0	517	1.21 (1.07, 1.36)	**0.0017** ^c^
nonDL PCBs	2,2′,3,5′-Tetrachlorobiphenyl [PCB 44]	Ages ≥ 50 years	0	108	0	527	1.16 (1.06, 1.27)	0.0016
nonDL PCBs	2,2′,4,5′-Tetrachlorobiphenyl [PCB 49]	Ages 12–19 years	1	59	0	511	1.20 (1.08, 1.34)	**0.00080** ^c^
nonDL PCBs	2,2′,3,4′,5′,6-Hexachlorobiphenyl [PCB 149]	Ages 12–19 years	5	59	0	513	1.21 (1.08, 1.36)	**0.0016** ^c^
nonDL PCBs	2,2′,3,3′,4,4′,5,5′,6-Nonachlorobiphenyl [PCB 206]	Mexican Americans	46	51	11	586	1.22 (1.09, 1.36)	**0.00052** ^c^
VOC Metabolites	N-Acetyl-S-(2-hydroxy-3-butenyl)-L-cysteine [MHB2]	Males	77	45	0	975	2.12 (1.59, 2.82)	**0.00000026** ^c^
VOC Metabolites	N-Acetyl-S-(2-hydroxyethyl)-L-cysteine [HEMA]	Non-Hispanic Blacks	30	58	2	452	0.80 (0.70, 0.92)	0.0022
VOC Metabolites	N-Acetyl-S-(3-hydroxypropyl)-L-cysteine [HPMA]	Males	0	117	0	975	1.12 (1.05, 1.21)	0.0015
VOC Metabolites	N-Acetyl-S-(phenyl)-L-cysteine [PMA]	Mexican Americans	48	9	5	188	1.82 (1.37, 2.43)	**0.000045** ^c^
Metals	Cadmium, urinary	Non-Hispanic Blacks	7	246	6	1,452	1.14 (1.04, 1.24)	0.0039
Metals	Uranium	Non-Hispanic Whites	27	98	1	736	1.29 (1.09, 1.53)	0.0034
Phthalates	Mono-benzyl phthalate [MBzP]	Ages 20–49 years	3	80	0	673	0.70 (0.59, 0.83)	**0.000046** ^c^
Phthalates	Mono-n-butyl phthalate [MnBP]	Ages 12–19 years	3	33	0	275	0.62 (0.46, 0.84)	**0.0018** ^c^
Anions	Nitrate	Ages ≥ 50 years	0	235	0	1,213	0.86 (0.77, 0.95)	0.0045
Tobacco Biomarkers^d^	Cotinine	Ages 12–19 years	21	178	0	2,267	0.57 (0.39, 0.83)	0.0035

Abbreviations: ANA = antinuclear antibodies; CI = confidence interval; MCR = mean concentration ratio; NODL = non-ortho dioxin-like; nonDL = non-dioxin-like; PCB = polychlorinated biphenyl; PCDD = polychlorinated dibenzo-p-dioxin; VOC = volatile organic compound; %BLOD = percent of participants with a concentration below the limit of detection; #ANA+ = number of ANA-positive participants with a detectable concentration; #SING = number of singleton clusters (i.e., number of sampling strata containing only one cluster); #ANAL = number of participants analyzed.

a The MCR is the ratio of mean concentrations for ANA-positive versus ANA-negative participants under a lognormal model for xenobiotic concentration; it is adjusted for sex, age, elderly status, race/ethnicity, BMI, PIR, smoking, birthplace, and cycle, but not for the sampling weights. The null MCR value is 1; MCR > 1 indicates a positive association between ANA and xenobiotic concentration; and MCR < 1 indicates a negative association between ANA and xenobiotic concentration. The CIs are based on variance estimates that make jackknife adjustments for correlations induced by the sampling strata and clusters.

b The P-values are displayed to two significant digits, are two-sided, and are not corrected for multiple comparisons.

c The bolded P-values are statistically significant at the 0.1 level after applying the FDR correction for multiple comparisons within each demographic group.

d The smoking covariate was not included in the model for tobacco biomarkers.

**Table 3:** Xenobiotics with positive ANA associations of greatest magnitude (MCR > 3/2) within demographic groups defined by sex, age, or race/ethnicity.

Xenobiotic Class	Xenobiotic Name [Alternative/Abbreviated Name]	Demographic Group	% BLOD	#ANA+	#SING	#ANAL	MCR (95% CI)^a^	P-value^b^
VOCs	1,4-Dichlorobenzene [Paradichlorobenzene]	Non-Hispanic Blacks	18	102	0	473	1.57 (0.87, 2.85)	0.134
VOCs	2,5-Dimethylfuran	Males	78	30	0	1,019	1.78 (1.14, 2.78)	0.012
VOCs	2,5-Dimethylfuran	Ages 20–49 years	77	37	0	887	1.54 (0.95, 2.51)	0.079
VOCs	2,5-Dimethylfuran	Non-Hispanic Whites	77	32	1	761	1.57 (0.85, 2.90)	0.149
VOCs	Benzene	Mexican Americans	49	33	5	214	1.76 (0.67, 4.60)	0.249
VOCs	Ethylbenzene	Mexican Americans	47	39	5	209	3.50 (0.48, 25.54)	0.216
VOCs	o-Xylene	Mexican Americans	57	31	5	219	2.17 (0.41, 11.53)	0.362
VOCs	Tetrachloroethene [Perchloroethylene]	Ages 20–49 years	75	60	0	821	1.89 (1.06, 3.38)	0.032
VOC Metabolites	2-Methylhippuric acid [2MHA]	Mexican Americans	3	17	5	188	2.16 (1.00, 4.70)	0.051
VOC Metabolites	2-Thioxothiazolidine-4-carboxylic acid [TTCA]	Ages 12–19 years	67	14	0	271	1.69 (0.90, 3.16)	0.101
VOC Metabolites	3- & 4-Methylhippuric acid [34MH]	Mexican Americans	0	17	5	188	1.53 (0.77, 3.02)	0.225
VOC Metabolites	N-Acetyl-S-(2-hydroxy-3-butenyl)-L-cysteine [MHB2]	Males	77	45	0	975	2.12 (1.59, 2.82)	**0.00000026** ^c^
VOC Metabolites	N-Acetyl-S-(2-hydroxy-3-butenyl)-L-cysteine [MHB2]	Ages 20–49 years	75	25	0	848	1.51 (1.06, 2.17)	0.024
VOC Metabolites	N-Acetyl-S-(2-hydroxy-3-butenyl)-L-cysteine [MHB2]	Non-Hispanic Blacks	79	23	2	452	1.60 (1.04, 2.47)	0.033
VOC Metabolites	N-Acetyl-S-(phenyl-2-hydroxyethyl)-L-cysteine [PHEM]	Mexican Americans	66	5	5	188	1.94 (0.40, 9.49)	0.412
VOC Metabolites	N-Acetyl-S-(phenyl)-L-cysteine [PMA]	Mexican Americans	48	9	5	188	1.82 (1.37, 2.43)	**0.000045** ^c^
Phthalates	Mono-(carboxyoctyl) phthalate [MCOP]	Non-Hispanic Blacks	0	71	1	379	1.51 (1.03, 2.19)	0.033
Herbicides	Alachlor mercapturate	Non-Hispanic Whites	68	8	2	336	1.56 (0.29, 8.51)	0.606
Fungicides	Pentachlorophenol	Mexican Americans	71	18	4	326	1.99 (0.99, 4.00)	0.053
Pyrethroid Insecticides	Cis-3-(2,2-dichlorovinyl)-2,2-dimethylcyclopropane carboxylic acid [*cis*-DCCA]	Non-Hispanic Whites	65	43	3	935	1.51 (1.01, 2.28)	0.046
OP/Other Insecticides	*para*-Nitrophenol	Ages 12–19 years	54	39	0	600	1.53 (0.74, 3.17)	0.251
OP/Other Insecticides	*para*-Nitrophenol	Mexican Americans	53	53	15	683	1.79 (0.74, 4.33)	0.198
Insect Repellents	3-(Diethylcarbamoyl)benzoic acid [DCBA or DEET acid]	Males	17	65	0	561	1.65 (1.01, 2.67)	0.044
Consumer Products	2,5-Dichlorophenol	Mexican Americans	2	76	9	541	1.52 (0.89, 2.61)	0.127
Consumer Products	Ethyl paraben	Mexican Americans	55	9	5	174	1.77 (0.37, 8.40)	0.474

Abbreviations: ANA = antinuclear antibodies; CI = confidence interval; MCR = mean concentration ratio; OP = organophosphorus; VOC = volatile organic compound; %BLOD = percent of participants with a concentration below the limit of detection; #ANA+ = number of ANA-positive participants with a detectable concentration; #SING = number of singleton clusters (i.e., number of sampling strata containing only one cluster); #ANAL = number of participants analyzed.

a The MCR is the ratio of mean concentrations for ANA-positive versus ANA-negative participants under a lognormal model for xenobiotic concentration; it is adjusted for sex, age, elderly status, race/ethnicity, BMI, PIR, smoking, birthplace, and cycle, but not for the sampling weights. The null MCR value is 1; MCR > 1 indicates a positive association between ANA and xenobiotic concentration; and MCR < 1 indicates a negative association between ANA and xenobiotic concentration. The CIs are based on variance estimates that make jackknife adjustments for correlations induced by the sampling strata and clusters.

b The P-values are displayed to three decimal places (or two significant digits if below 0.100), are two-sided, and are not corrected for multiple comparisons.

c The bolded P-values are statistically significant at the 0.1 level after applying the FDR correction for multiple comparisons within each demographic group.

**Table 4:** Xenobiotics with negative ANA associations of greatest magnitude (MCR < 2/3) within demographic groups defined by sex, age, or race/ethnicity.

Xenobiotic Class	Xenobiotic Name [Alternative/Abbreviated Name]	Demographic Group	% BLOD	#ANA+	#SING	#ANAL	MCR (95% CI)^a^	P-value^b^
nonDL PCBs	2,2′,3,3′,4,5,5′-Heptachlorobiphenyl [PCB 172]	Ages 12–19 years	88	10	0	969	0.58 (0.35, 0.96)	0.034
nonDL PCBs	2,2′,3,3′,4,4′,5,6-Octachlorobiphenyl [PCB 195]	Ages 12–19 years	84	5	0	499	0.55 (0.23, 1.33)	0.187
VOCs	Tribromomethane [Bromoform]	Ages 12–19 years	81	6	0	356	0.50 (0.21, 1.19)	0.116
VOC Metabolites	N-Acetyl-S-(phenyl-2-hydroxyethyl)-L-cysteine [PHEM]	Ages 12–19 years	65	5	0	271	0.48 (0.29, 0.82)	0.0072
Phthalates	Mono-n-butyl phthalate [MnBP]	Ages 12–19 years	3	33	0	275	0.62 (0.46, 0.84)	**0.0018** ^c^
Phthalates	Mono-n-butyl phthalate [MnBP]	Mexican Americans	4	15	5	174	0.55 (0.29, 1.02)	0.057
Herbicides	Alachlor mercapturate	Males	65	5	0	449	0.25 (0.04, 1.74)	0.162
OP/DPM Insecticides	Diethyldithiophosphate [DEDTP]	Males	79	27	0	2,319	0.63 (0.38, 1.05)	0.076
OP/Other Insecticides	Malathion dicarboxylic acid	Males	49	8	0	447	0.53 (0.22, 1.28)	0.157
OP/Other Insecticides	Malathion dicarboxylic acid	Non-Hispanic Whites	50	9	2	337	0.61 (0.28, 1.32)	0.211
OP/Other Insecticides	Malathion dicarboxylic acid	Non-Hispanic Blacks	50	11	4	172	0.58 (0.19, 1.77)	0.340
Insect Repellents	N,N-Diethyl-*meta*-toluamide [DEET]	Non-Hispanic Whites	89	17	3	1,375	0.66 (0.33, 1.32)	0.244
PFAS	2-(N-methyl-perfluorooctane sulfonamido) acetic acid [MeFOSAA]	Ages 12–19 years	45	19	0	263	0.66 (0.45, 0.97)	0.033
Consumer Products	Butyl paraben	Ages 20–49 years	70	24	0	673	0.42 (0.20, 0.91)	0.029
Consumer Products	Propyl paraben [n-Propyl paraben]	Ages ≥ 50 years	9	115	0	600	0.60 (0.30, 1.19)	0.143
Consumer Products	Triclosan	Ages 12–19 years	22	68	0	797	0.58 (0.35, 0.95)	0.030
Tobacco Biomarkers^d^	Cotinine	Ages 12–19 years	21	178	0	2,267	0.57 (0.39, 0.83)	0.0035
Tobacco Biomarkers^d^	Cotinine	Ages 20–49 years	17	506	0	5,356	0.63 (0.44, 0.91)	0.013
Tobacco Biomarkers^d^	Cotinine	Non-Hispanic Whites	23	497	2	5,175	0.60 (0.38, 0.95)	0.030
Tobacco Biomarkers^d^	Cotinine	Mexican Americans	26	263	19	2,620	0.56 (0.32, 0.97)	0.040
Tobacco Biomarkers^d^	4-(Methylnitrosamino)-1-(3-pyridyl)-1-butanol [NNAL]	Ages 20–49 years	26	161	0	1,621	0.61 (0.40, 0.92)	0.018
Tobacco Biomarkers^d^	4-(Methylnitrosamino)-1-(3-pyridyl)-1-butanol [NNAL]	Mexican Americans	33	34	2	433	0.56 (0.28, 1.10)	0.091

Abbreviations: ANA = antinuclear antibodies; CI = confidence interval; DPM = dialkyl phosphate metabolites; MCR = mean concentration ratio; nonDL = non-dioxinlike; OP = organophosphorus; PCB = polychlorinated biphenyl; PFAS = perfluoroalkyl and polyfluoroalkyl substances; VOC = volatile organic compound; %BLOD = percent of participants with a concentration below the limit of detection; #ANA+ = number of ANA-positive participants with a detectable concentration; #SING = number of singleton clusters (i.e., number of sampling strata containing only one cluster); #ANAL = number of participants analyzed.

a The MCR is the ratio of mean concentrations for ANA-positive versus ANA-negative participants under a lognormal model for xenobiotic concentration; it is adjusted for sex, age, elderly status, race/ethnicity, BMI, PIR, smoking, birthplace, and cycle, but not for the sampling weights. The null MCR value is 1; MCR > 1 indicates a positive association between ANA and xenobiotic concentration; and MCR < 1 indicates a negative association between ANA and xenobiotic concentration. The CIs are based on variance estimates that make jackknife adjustments for correlations induced by the strata and clusters.

b The P-values are displayed to three decimal places (or two significant digits if below 0.100), are two-sided, and are not corrected for multiple comparisons.

c The bolded P-value is statistically significant at the 0.1 level after applying the FDR correction for multiple comparisons within each demographic group.

d The smoking covariate was not included in the model for tobacco biomarkers.

**Table 5:** Possible exposure sources and prior toxicologic associations for the xenobiotics with ANA associations of greatest statistical significance after correcting for multiple comparisons (P_FDR_ < 0.1).

Xenobiotic Class	Xenobiotic Name	Possible Exposure Source (references)^a^	Prior Toxicologic Associations (references)^a^	Demographic Group	MCR (95% CI)^b^	P_FDR_^c^
*Xenobiotics with Positive ANA Associations*						
NODL PCBs	PCB 81	High-fat foods such as dairy products, eggs, and fish and animal fats; rare waste site and occupational exposures (Ododo and Wabalo 2019)	Possible hepatotoxicity, endocrine disruption, genotoxicity, neurotoxicity, carcinogenicity, immunosuppression (Ododo and Wabalo 2019; Lu and Wu 1985)	Mexican Americans	1.26 (1.12, 1.42)	0.013
nonDL PCBs	PCB 44			Ages 12–19 years	1.21 (1.07, 1.36)	0.081
nonDL PCBs	PCB 49			Ages 12–19 years	1.20 (1.08, 1.34)	0.081
nonDL PCBs	PCB 149			Ages 12–19 years	1.21 (1.08, 1.36)	0.081
nonDL PCBs	PCB 206			Mexican Americans	1.22 (1.09, 1.36)	0.030
VOC Metabolites	MHB2	Metabolite of 1,3-butadiene - vehicular exhaust; petrochemical plants; smoking; burning of wood, plastics, and rubber; hazardous waste sites (Kim et al. 2001; Nieto et al. 2021)	Possible neurotoxicity, endocrine disruption, cancer (Kirman et al. 2010)	Males	2.12 (1.59, 2.82)	0.000048
VOC Metabolites	PMA	Metabolite of benzene - occupational exposures in petroleum and chemical industries, tobacco smoke and other air pollutants (NTP 2021; Medeiros et al. 1997; NCBI 2022a)	Benzene is a carcinogen, neurotoxin, hematologic toxin and immunotoxin (NCBI 2022a); PMA causes skin, eye and respiratory irritation - possible leukopenia with neutropenia, thrombopenia, and some anemia with preferential effect on lymphocytes (NCBI 2022b)	Mexican Americans	1.82 (1.37, 2.43)	0.0080
*Xenobiotics with Negative ANA Associations*						
Phthalates	MBzP	Exposure to polyvinyl chloride plastics; indoor air inhalation (CDC 2021b; Chen et al. 2017)	Possible role in asthma and allergies, obesity, endocrine disruption, insulin resistance, infertility, immune regulation (Stahlhut et al. 2007; Tranfo et al., 2012; Wang et al. 2020)	Ages 20–49 years	0.70 (0.59, 0.83)	0.0086
Phthalates	MnBP	Exposure to polyvinyl chloride plastics; plastic container or food packaging (CDC 2021b; Chen et al. 2017)	Possible role in asthma and allergies, endocrine disruption, hepatotoxicity, immune alteration (Hansen et al. 2015; Ema 2002)	Ages 12–19 years	0.62 (0.46, 0.84)	0.081

Abbreviations: ANA = antinuclear antibodies; CI = confidence interval; MBzP = mono-benzyl phthalate; MCR = mean concentration ratio; MHB2 = N-acetyl-S-(2-hydroxy-3-butenyl)-L-cysteine; MnBP = mono-n-butyl phthalate; NODL = non-ortho dioxin-like; nonDL = non-dioxin-like; P_FDR_ = false-discovery-rate-corrected P-value; PCB = polychlorinated biphenyl; PCB 44 = 2,2′,3,5′-tetrachlorobiphenyl; PCB 49 = 2,2′,4,5′-tetrachlorobiphenyl; PCB 81 = 3,4,4′,5-tetrachlorobiphenyl; PCB 149 = 2,2′,3,4′,5′,6-hexachlorobiphenyl; PCB 206 = 2,2′,3,3′,4,4′,5,5′,6-nonachlorobiphenyl; PMA = N-acetyl-S-(phenyl)-L-cysteine; VOC = volatile organic compound.

a Data are limited for some xenobiotics, but given common structures and mechanisms for many xenobiotics within a class, information is listed for the class when available and qualified as a possible association.

b The MCR is the ratio of mean concentrations for ANA-positive versus ANA-negative participants under a lognormal model for xenobiotic concentration; it is adjusted for sex, age, elderly status, race/ethnicity, BMI, PIR, smoking, birthplace, and cycle, but not for the sampling weights. The null MCR value is 1; MCR > 1 indicates a positive association between ANA and xenobiotic concentration; and MCR < 1 indicates a negative association between ANA and xenobiotic concentration. The CIs are based on variance estimates that make jackknife adjustments for correlations induced by the sampling strata and clusters.

c The P-values are displayed to two significant digits, are two-sided, and incorporate an FDR correction for multiple comparisons within each demographic group.
