# Supplementary material for: Expanded assessment of xenobiotic associations with antinuclear antibodies in the United States, 1988–2012
Source: Environ Int. Author manuscript; Available in PMC 2023 Oct 18. (PMC9792625; doi:10.1016/j.envint.2022.107376)
Supplement: MMC5 [file NIHMS1820607-supplement-MMC5.docx]

| Supplemental Table 5a. Covariate-adjusted estimates of ANA associations with dioxin-like compounds, for males stratified by age. | | | | | |
| --- | --- | --- | --- | --- | --- |
|  |  |  |  |  |  |
|  |  |  |  |  |  |
| Xenobiotic Class | |  | Estimated MCR (and 95% CI) for Assessing Association between ANA and Xenobiotic ^a^ | | |
|  | Xenobiotic Name [Alternative/Abbreviated Name] |  | Males, Ages 12-19 years | Males, Ages 20-49 years | Males, Ages ≥50 years |
|  |  |  |  |  |  |
| Polychlorinated Dibenzo-p-dioxins [PCDDs] | |  |  |  |  |
|  | 2,3,7,8-Tetrachlorodibenzo-p-dioxin [2,3,7,8-TCDD] |  | -----^b^ | -----^b^ | 1.02 (0.72, 1.46) |
|  | 1,2,3,7,8-Pentachlorodibenzo-p-dioxin [1,2,3,7,8-PeCDD] |  | -----^b^ | 0.87 (0.50, 1.50) | 1.19 (0.93, 1.52) |
|  | 1,2,3,4,7,8-Hexachlorodibenzo-p-dioxin [1,2,3,4,7,8-HxCDD] |  | -----^b^ | -----^b^ | 1.35 (1.11, 1.65) |
|  | 1,2,3,6,7,8-Hexachlorodibenzo-p-dioxin [1,2,3,6,7,8-HxCDD] |  | 0.89 (0.59, 1.34) | 1.12 (0.87, 1.44) | 1.18 (0.97, 1.44) |
|  | 1,2,3,7,8,9-Hexachlorodibenzo-p-dioxin [1,2,3,7,8,9-HxCDD] |  | -----^b^ | 0.98 (0.63, 1.52) | 1.33 (1.08, 1.63) |
|  | 1,2,3,4,6,7,8-Heptachlorodibenzo-p-dioxin [1,2,3,4,6,7,8-HpCDD] |  | 0.81 (0.59, 1.10) | 1.27 (1.02, 1.57) | 1.17 (0.97, 1.41) |
|  | 1,2,3,4,6,7,8,9-Octachlorodibenzo-p-dioxin [1,2,3,4,6,7,8,9-OCDD] |  | 0.69 (0.50, 0.96) | 1.34 (1.07, 1.68) | 1.18 (0.97, 1.44) |
|  | TEQ mixture of PCDDs |  | 0.89 (0.73, 1.08) | 1.05 (0.90, 1.23) | 1.13 (0.99, 1.30) |
|  |  |  |  |  |  |
| Polychlorinated Dibenzofurans [PCDFs] | |  |  |  |  |
|  | 2,3,4,7,8-Pentachlorodibenzofuran [2,3,4,7,8-PeCDF] |  | 1.03 (0.79, 1.35) | 1.08 (0.81, 1.44) | 0.97 (0.81, 1.16) |
|  | 2,3,7,8-Tetrachlorodibenzofuran [2,3,7,8-TCDF] |  | -----^b^ | -----^b^ | -----^b^ |
|  | 1,2,3,4,7,8-Hexachlorodibenzofuran [1,2,3,4,7,8-HxCDF] |  | -----^b^ | 1.25 (1.02, 1.53) | 1.01 (0.87, 1.18) |
|  | 1,2,3,6,7,8-Hexachlorodibenzofuran [1,2,3,6,7,8-HxCDF] |  | 1.15 (0.80, 1.66) | 1.31 (1.00, 1.71) | 1.01 (0.87, 1.17) |
|  | 1,2,3,7,8,9-Hexachlorodibenzofuran [1,2,3,7,8,9-HxCDF] |  | -----^b^ | -----^b^ | -----^b^ |
|  | 2,3,4,6,7,8-Hexachlorodibenzofuran [2,3,4,6,7,8-HxCDF] |  | -----^b^ | -----^b^ | -----^b^ |
|  | 1,2,3,7,8-Pentachlorodibenzofuran [1,2,3,7,8-PeCDF] |  | -----^b^ | -----^b^ | -----^b^ |
|  | 1,2,3,4,6,7,8-Heptachlorodibenzofuran [1,2,3,4,6,7,8-HpCDF] |  | 0.73 (0.55, 0.99) | 1.03 (0.75, 1.40) | 0.97 (0.85, 1.11) |
|  | 1,2,3,4,7,8,9-Heptachlorodibenzofuran [1,2,3,4,7,8,9-HpCDF] |  | -----^b^ | -----^b^ | -----^b^ |
|  | 1,2,3,4,6,7,8,9-Octachlorodibenzofuran [1,2,3,4,6,7,8,9-OCDF] |  | -----^b^ | -----^b^ | 1.22 (0.77, 1.95) |
|  | TEQ mixture of PCDFs |  | 0.96 (0.76, 1.21) | 1.16 (0.93, 1.46) | 0.98 (0.85, 1.14) |
|  |  |  |  |  |  |
| Non-Ortho Dioxin-Like Polychlorinated Biphenyls [NODL PCBs] | |  |  |  |  |
|  | 3,3',4,4',5-Pentachlorobiphenyl [PCB 126] |  | 0.95 (0.74, 1.20) | 1.23 (0.95, 1.59) | 1.14 (0.94, 1.38) |
|  | 3,3',4,4',5,5'-Hexachlorobiphenyl [PCB 169] |  | 0.94 (0.58, 1.52) | 1.19 (0.96, 1.46) | 1.10 (0.95, 1.26) |
|  | 3,4,4',5-Tetrachlorobiphenyl [PCB 81] |  | -----^b^ | -----^b^ | 1.16 (0.82, 1.65) |
|  | TEQ mixture of NODL PCBs |  | 0.94 (0.77, 1.15) | 1.23 (0.98, 1.53) | 1.12 (0.95, 1.32) |
|  |  |  |  |  |  |
| Mono-Ortho Dioxin-Like Polychlorinated Biphenyls [MODL PCBs] | |  |  |  |  |
|  | 2,3,3',4,4'-Pentachlorobiphenyl [PCB 105] |  | 1.00 (0.78, 1.28) | 1.46 (0.83, 2.58) | 0.95 (0.66, 1.35) |
|  | 2,3',4,4',5-Pentachlorobiphenyl [PCB 118] |  | 0.89 (0.73, 1.08) | 1.46 (1.06, 2.00) | 1.00 (0.78, 1.29) |
|  | 2,3,3',4,4',5-Hexachlorobiphenyl [PCB 156] |  | 0.90 (0.50, 1.60) | 1.35 (1.00, 1.82) | 1.02 (0.86, 1.20) |
|  | 2,3,3',4,4',5'-Hexachlorobiphenyl [PCB 157] |  | -----^b^ | 1.28 (0.88, 1.86) | 1.06 (0.83, 1.36) |
|  | 2,3',4,4',5,5'-Hexachlorobiphenyl [PCB 167] |  | -----^b^ | 1.16 (0.64, 2.08) | 1.06 (0.76, 1.46) |
|  | 2,3,3',4,4',5,5'-Heptachlorobiphenyl [PCB 189] |  | -----^b^ | -----^b^ | 0.93 (0.52, 1.67) |
|  | TEQ mixture of MODL PCBs |  | 0.87 (0.67, 1.13) | 1.35 (1.00, 1.82) | 1.01 (0.83, 1.24) |
|  |  |  |  |  |  |
| All Dioxin-Like Compounds | |  |  |  |  |
|  | TEQ mixture of PCDDs, PCDFs, NODL PCBs, and MODL PCBs |  | 0.91 (0.77, 1.07) | 1.12 (0.95, 1.31) | 1.11 (0.97, 1.26) |
|  |  |  |  |  |  |
|  |  |  |  |  |  |
| Abbreviations: ANA = antinuclear antibodies; CI = confidence interval; MCR = mean concentration ratio; TEF = toxic equivalency factor; TEQ = toxic equivalent. | | | | | |
| Note: green highlighting indicates 0.01 < P-value ≤ 0.05; yellow highlighting indicates 0.001 < P-value ≤ 0.01; and red highlighting indicates P-value ≤ 0.001. | | | | | |
| Note: the dioxin-like compounds within each class are arranged by decreasing TEF value to match their order in Supplemental Table 1a. | | | | | |
| ^a^ The MCR is the ratio of mean concentrations for ANA-positive versus ANA-negative participants under a lognormal model for xenobiotic concentration; it is adjusted for sex, age, elderly status, race/ethnicity, BMI, PIR, smoking, birthplace, and cycle, but not for the sampling weights. The null MCR value is 1; MCR>1 indicates a positive association between ANA and xenobiotic concentration; and MCR<1 indicates a negative association between ANA and xenobiotic concentration. The CIs are based on variance estimates that make jackknife adjustments for correlations induced by the strata and clusters. | | | | | |
| ^b^ No estimates are shown if <5 ANA-positive participants had detectable concentrations or if >90% of all concentrations were below the limit of detection. | | | | | |

| Supplemental Table 5b. Covariate-adjusted estimates of ANA associations with non-dioxin-like polychlorinated biphenyls, for males stratified by age. | | | | |
| --- | --- | --- | --- | --- |
|  |  |  |  |  |
|  |  |  |  |  |
|  |  | Estimated MCR (and 95% CI) for Assessing Association between ANA and Xenobiotic ^a^ | | |
| Xenobiotic Name [Alternative/Abbreviated Name] |  | Males, Ages 12-19 years | Males, Ages 20-49 years | Males, Ages ≥50 years |
|  |  |  |  |  |
| 2,4,4'-Trichlorobiphenyl [PCB 28] |  | 1.27 (0.98, 1.63) | 0.95 (0.67, 1.34) | 1.14 (0.99, 1.32) |
| 2,2',3,5'-Tetrachlorobiphenyl [PCB 44] |  | 1.28 (0.93, 1.78) | 0.94 (0.65, 1.36) | 1.27 (1.09, 1.47) |
| 2,2',4,5'-Tetrachlorobiphenyl [PCB 49] |  | 1.26 (0.92, 1.74) | 0.96 (0.62, 1.50) | 1.26 (1.06, 1.50) |
| 2,2',5,5'-Tetrachlorobiphenyl [PCB 52] |  | 1.24 (0.92, 1.66) | 0.87 (0.60, 1.25) | 1.21 (1.04, 1.40) |
| 2,3',4,4'-Tetrachlorobiphenyl [PCB 66] |  | 1.14 (0.93, 1.39) | 1.19 (0.91, 1.56) | 1.14 (0.93, 1.39) |
| 2,4,4',5-Tetrachlorobiphenyl [PCB 74] |  | 1.07 (0.94, 1.23) | 1.39 (1.14, 1.71) | 1.00 (0.80, 1.25) |
| 2,2’,3,4,5’-Pentachlorobiphenyl [PCB 87] |  | 1.05 (0.64, 1.71) | 1.41 (0.98, 2.04) | 1.37 (1.04, 1.82) |
| 2,2',4,4',5-Pentachlorobiphenyl [PCB 99] |  | 0.97 (0.82, 1.15) | 1.35 (0.98, 1.86) | 0.92 (0.73, 1.15) |
| 2,2',4,5,5'-Pentachlorobiphenyl [PCB 101] |  | 1.09 (0.83, 1.43) | 0.88 (0.52, 1.49) | 1.07 (0.86, 1.32) |
| 2,3,3’,4’,6-Pentachlorobiphenyl [PCB 110] |  | 1.13 (0.81, 1.59) | 1.12 (0.76, 1.65) | 1.20 (0.99, 1.46) |
| 2,2',3,3',4,4'-Hexachlorobiphenyl [PCB 128] |  | -----^b^ | -----^b^ | 0.72 (0.31, 1.68) |
| 2,2',3,4,4',5'-Hexachlorobiphenyl [PCB 138] ^c^ |  | 0.87 (0.69, 1.09) | 1.39 (1.09, 1.77) | 0.96 (0.78, 1.18) |
| 2,2',3,4',5,5'-Hexachlorobiphenyl [PCB 146] |  | 0.91 (0.71, 1.16) | 1.59 (1.17, 2.15) | 0.99 (0.82, 1.20) |
| 2,2’,3,4’,5’,6-Hexachlorobiphenyl [PCB 149] |  | 1.06 (0.75, 1.50) | 1.08 (0.77, 1.52) | 1.21 (0.89, 1.63) |
| 2,2’,3,5,5’,6-Hexachlorobiphenyl [PCB 151] |  | 1.10 (0.76, 1.59) | 1.40 (0.93, 2.11) | 1.24 (0.93, 1.64) |
| 2,2',4,4',5,5'-Hexachlorobiphenyl [PCB 153] |  | 0.87 (0.69, 1.09) | 1.33 (1.06, 1.65) | 0.96 (0.79, 1.17) |
| 2,2',3,3',4,4',5-Heptachlorobiphenyl [PCB 170] |  | 0.99 (0.72, 1.35) | 1.24 (1.01, 1.51) | 1.01 (0.88, 1.17) |
| 2,2',3,3',4,5,5'-Heptachlorobiphenyl [PCB 172] |  | -----^b^ | 1.17 (0.85, 1.62) | 0.97 (0.76, 1.23) |
| 2,2',3,3',4,5',6'-Heptachlorobiphenyl [PCB 177] |  | 0.94 (0.49, 1.83) | 1.54 (0.99, 2.39) | 0.98 (0.75, 1.27) |
| 2,2',3,3',5,5',6-Heptachlorobiphenyl [PCB 178] |  | 0.69 (0.32, 1.49) | 1.38 (0.87, 2.19) | 1.02 (0.87, 1.21) |
| 2,2',3,4,4',5,5'-Heptachlorobiphenyl [PCB 180] |  | 0.90 (0.65, 1.25) | 1.22 (0.99, 1.50) | 1.02 (0.88, 1.18) |
| 2,2',3,4,4',5',6-Heptachlorobiphenyl [PCB 183] |  | 1.02 (0.74, 1.42) | 1.46 (1.02, 2.09) | 0.92 (0.73, 1.17) |
| 2,2',3,4',5,5',6-Heptachlorobiphenyl [PCB 187] |  | 0.96 (0.72, 1.30) | 1.29 (0.97, 1.72) | 0.98 (0.83, 1.15) |
| 2,2’,3,3’,4,4’,5,5’-Octachlorobiphenyl [PCB 194] |  | 1.41 (0.72, 2.77) | 1.24 (0.86, 1.78) | 1.08 (0.93, 1.25) |
| 2,2’,3,3’,4,4’,5,6-Octachlorobiphenyl [PCB 195] |  | -----^b^ | 0.68 (0.26, 1.80) | 1.14 (0.78, 1.67) |
| 2,2’,3,3’,4,4’,5,6’-Octachlorobiphenyl [PCB 196] ^d^ |  | 0.96 (0.55, 1.69) | 1.37 (1.00, 1.88) | 1.06 (0.90, 1.26) |
| 2,2’,3,3’,4,5,5’,6-Octachlorobiphenyl [PCB 199] |  | 0.98 (0.51, 1.88) | 1.21 (0.94, 1.56) | 1.09 (0.89, 1.34) |
| 2,2’,3,3’,4,4’,5,5’,6-Nonachlorobiphenyl [PCB 206] |  | 1.05 (0.68, 1.62) | 1.03 (0.85, 1.24) | 1.12 (0.92, 1.37) |
| 2,2',3,3',4,4',5,5',6,6'-Decachlorobiphenyl [PCB 209] |  | 1.16 (0.78, 1.71) | 1.02 (0.87, 1.20) | 1.09 (0.89, 1.32) |
|  |  |  |  |  |
|  |  |  |  |  |
| Abbreviations: ANA = antinuclear antibodies; CI = confidence interval; MCR = mean concentration ratio. | | | | |
| Note: green highlighting indicates 0.01 < P-value ≤ 0.05; yellow highlighting indicates 0.001 < P-value ≤ 0.01; and red highlighting indicates P-value ≤ 0.001. | | | | |
| ^a^ The MCR is the ratio of mean concentrations for ANA-positive versus ANA-negative participants under a lognormal model for xenobiotic concentration; it is adjusted for sex, age, elderly status, race/ethnicity, BMI, PIR, smoking, birthplace, and cycle, but not for the sampling weights. The null MCR value is 1; MCR>1 indicates a positive association between ANA and xenobiotic concentration; and MCR<1 indicates a negative association between ANA and xenobiotic concentration. The CIs are based on variance estimates that make jackknife adjustments for correlations induced by the strata and clusters. | | | | |
| ^b^ No estimates are shown if <5 ANA-positive participants had detectable concentrations or if >90% of all concentrations were below the limit of detection. | | | | |
| ^c^ The information in this row pertains to both 2,2',3,4,4',5'-Hexachlorobiphenyl [PCB 138] and 2,3,3’,4,4’,6-Hexachlorobiphenyl [PCB 158]. | | | | |
| ^d^ The information in this row pertains to both 2,2’,3,3’,4,4’,5,6’-Octachlorobiphenyl [PCB 196] and 2,2’,3,4,4’,5,5’,6-Octachlorobiphenyl [PCB 203]. | | | | |

| Supplemental Table 5c. Covariate-adjusted estimates of ANA associations with volatile organic compounds, for males stratified by age. | | | | |
| --- | --- | --- | --- | --- |
|  |  |  |  |  |
|  |  |  |  |  |
|  |  | Estimated MCR (and 95% CI) for Assessing Association between ANA and Xenobiotic ^a^ | | |
| Xenobiotic Name [Alternative/Abbreviated Name] |  | Males, Ages 12-19 years | Males, Ages 20-49 years | Males, Ages ≥50 years |
|  |  |  |  |  |
| 1,1-Dichloroethane |  | -----^b^ | -----^b^ | -----^b^ |
| 1,1-Dichloroethene [Vinylidene chloride] |  | -----^b^ | -----^b^ | -----^b^ |
| 1,1,1-Trichloroethane [Methyl chloroform] |  | -----^b^ | -----^b^ | -----^b^ |
| 1,1,1,2-Tetrachloroethane |  | -----^b^ | -----^b^ | -----^b^ |
| 1,1,2-Trichloroethane |  | -----^b^ | -----^b^ | -----^b^ |
| 1,1,2,2-Tetrachloroethane |  | -----^b^ | -----^b^ | -----^b^ |
| 1,2-Dibromo-3-chloropropane [DBCP] |  | -----^b^ | -----^b^ | -----^b^ |
| 1,2-Dibromoethane |  | -----^b^ | -----^b^ | -----^b^ |
| 1,2-Dichlorobenzene [o-Dichlorobenzene] |  | -----^b^ | -----^b^ | -----^b^ |
| 1,2-Dichloroethane [Ethylene dichloride] |  | -----^b^ | -----^b^ | -----^b^ |
| 1,2-Dichloropropane |  | -----^b^ | -----^b^ | -----^b^ |
| 1,2,3-Trichloropropane |  | -----^b^ | -----^b^ | -----^b^ |
| 1,3-Dichlorobenzene |  | -----^b^ | -----^b^ | -----^b^ |
| 1,4-Dichlorobenzene [Paradichlororbenzene] |  | 0.84 (0.29, 2.43) | 1.81 (0.98, 3.35) | 0.94 (0.47, 1.89) |
| 1,4-Dioxane |  | -----^b^ | -----^b^ | -----^b^ |
| 2-Butanone |  | -----^b^ | -----^b^ | -----^b^ |
| 2,5-Dimethylfuran |  | -----^b^ | 2.51 (0.85, 7.40) | 1.41 (0.88, 2.28) |
| Acetone |  | -----^b^ | -----^b^ | -----^b^ |
| Benzene |  | -----^b^ | 1.41 (0.92, 2.17) | 1.22 (0.78, 1.91) |
| Bromodichloromethane |  | 1.61 (0.85, 3.04) | 0.74 (0.38, 1.46) | 1.10 (0.78, 1.54) |
| Chlorobenzene |  | -----^b^ | -----^b^ | -----^b^ |
| cis-1,2-Dichloroethene [cis-1,2-Dichloroethelene] |  | -----^b^ | -----^b^ | -----^b^ |
| Dibromochloromethane [Chlorodibromomethane] |  | 1.29 (0.48, 3.51) | 0.74 (0.33, 1.66) | 1.10 (0.73, 1.67) |
| Dibromomethane |  | -----^b^ | -----^b^ | -----^b^ |
| Dichloromethane [Methylene chloride] |  | -----^b^ | -----^b^ | -----^b^ |
| Ethylbenzene |  | 1.32 (0.73, 2.39) | 1.19 (0.79, 1.79) | 1.16 (0.85, 1.58) |
| Furan |  | -----^b^ | 1.65 (0.94, 2.89) | 1.13 (0.81, 1.58) |
| Hexachloroethane |  | -----^b^ | -----^b^ | -----^b^ |
| Isopropylbenzene [Cumene] |  | -----^b^ | -----^b^ | -----^b^ |
| m-/p-Xylene |  | 1.08 (0.77, 1.49) | 0.93 (0.77, 1.12) | 1.10 (0.93, 1.32) |
| Methyl-tert-butyl ether [MTBE] |  | 1.15 (0.64, 2.07) | 0.92 (0.58, 1.47) | 1.38 (0.87, 2.20) |
| n-Hexane [Hexane] |  | -----^b^ | -----^b^ | -----^b^ |
| Nitrobenzene |  | -----^b^ | -----^b^ | -----^b^ |
| Nitromethane |  | 1.13 (1.01, 1.27) | 0.97 (0.84, 1.12) | 0.97 (0.88, 1.07) |
| o-Xylene |  | 1.21 (0.70, 2.11) | 0.96 (0.55, 1.66) | 1.11 (0.87, 1.41) |
| Styrene |  | -----^b^ | -----^b^ | -----^b^ |
| Tetrachloroethene [Perchloroethylene] |  | -----^b^ | 1.33 (0.53, 3.33) | 1.05 (0.42, 2.60) |
| Tetrachloromethane [Carbon tetrachloride] |  | -----^b^ | -----^b^ | -----^b^ |
| Toluene |  | -----^b^ | -----^b^ | -----^b^ |
| trans-1,2-Dichloroethene |  | -----^b^ | -----^b^ | -----^b^ |
| Tribromomethane [Bromoform] |  | 0.92 (0.25, 3.42) | 0.66 (0.15, 2.79) | 1.26 (0.79, 2.00) |
| Trichloroethene [Trichloroethylene] |  | -----^b^ | -----^b^ | -----^b^ |
| Trichloromethane [Chloroform] |  | 1.27 (0.81, 1.99) | 0.76 (0.48, 1.21) | 0.96 (0.73, 1.27) |
|  |  |  |  |  |
|  |  |  |  |  |
| Abbreviations: ANA = antinuclear antibodies; CI = confidence interval; MCR = mean concentration ratio. | | | | |
| Note: green highlighting indicates 0.01 < P-value ≤ 0.05; yellow highlighting indicates 0.001 < P-value ≤ 0.01; and red highlighting indicates P-value ≤ 0.001. | | | | |
| ^a^ The MCR is the ratio of mean concentrations for ANA-positive versus ANA-negative participants under a lognormal model for xenobiotic concentration; it is adjusted for sex, age, elderly status, race/ethnicity, BMI, PIR, smoking, birthplace, and cycle, but not for the sampling weights. The null MCR value is 1; MCR>1 indicates a positive association between ANA and xenobiotic concentration; and MCR<1 indicates a negative association between ANA and xenobiotic concentration. The CIs are based on variance estimates that make jackknife adjustments for correlations induced by the strata and clusters. | | | | |
| ^b^ No estimates are shown if <5 ANA-positive participants had detectable concentrations or if >90% of all concentrations were below the limit of detection. | | | | |

| Supplemental Table 5d. Covariate-adjusted estimates of ANA associations with volatile organic compound metabolites, for males stratified by age. | | | | |
| --- | --- | --- | --- | --- |
|  |  |  |  |  |
|  |  |  |  |  |
|  |  | Estimated MCR (and 95% CI) for Assessing Association between ANA and Xenobiotic ^a^ | | |
| Xenobiotic Name [Alternative/Abbreviated Name] |  | Males, Ages 12-19 years | Males, Ages 20-49 years | Males, Ages ≥50 years |
|  |  |  |  |  |
| 2-Aminothiazoline-4-carboxylic acid [ATCA] |  | 0.80 (0.41, 1.57) | 1.10 (0.78, 1.54) | 0.98 (0.81, 1.18) |
| 2-Methylhippuric acid [2MHA] |  | 0.69 (0.39, 1.24) | 1.00 (0.66, 1.50) | 1.07 (0.76, 1.49) |
| 2-Thioxothiazolidine-4-carboxylic acid [TTCA] |  | 1.10 (0.44, 2.74) | 1.24 (0.76, 2.04) | 0.77 (0.39, 1.54) |
| 3- & 4-Methylhippuric acid [34MH] |  | 0.67 (0.41, 1.10) | 1.07 (0.74, 1.55) | 1.14 (0.86, 1.50) |
| Mandelic acid [MADA] |  | 0.98 (0.72, 1.33) | 1.23 (0.89, 1.68) | 1.04 (0.91, 1.18) |
| N-Acetyl-S-(1-hydroxymethyl-2-propenyl)-L-cysteine [MHB1] |  | -----^b^ | -----^b^ | -----^b^ |
| N-Acetyl-S-(1,2-dichlorovinyl)-L-cysteine [1DCV] |  | -----^b^ | -----^b^ | -----^b^ |
| N-Acetyl-S-(2-carbamoyl-2-hydroxyethyl)-L-cysteine [GAMA] |  | 1.10 (0.69, 1.76) | 1.03 (0.75, 1.42) | 0.91 (0.76, 1.09) |
| N-Acetyl-S-(2-carbamoylethyl)-L-cysteine [AAMA] |  | 0.95 (0.66, 1.37) | 1.00 (0.76, 1.30) | 0.89 (0.79, 1.02) |
| N-Acetyl-S-(2-carboxyethyl)-L-cysteine [CEMA] |  | 1.31 (0.94, 1.83) | 1.08 (0.83, 1.41) | 0.88 (0.75, 1.02) |
| N-Acetyl-S-(2-cyanoethyl)-L-cysteine [CYMA] |  | 1.06 (0.62, 1.83) | 1.28 (0.80, 2.06) | 1.02 (0.78, 1.32) |
| N-Acetyl-S-(2-hydroxy-3-butenyl)-L-cysteine [MHB2] |  | -----^b^ | 2.77 (1.35, 5.68) | 1.76 (1.24, 2.51) |
| N-Acetyl-S-(2-hydroxyethyl)-L-cysteine [HEMA] |  | 0.94 (0.59, 1.49) | 0.94 (0.61, 1.46) | 0.81 (0.60, 1.10) |
| N-Acetyl-S-(2-hydroxypropyl)-L-cysteine [HPM2] |  | 1.07 (0.62, 1.86) | 1.16 (0.87, 1.55) | 1.08 (0.87, 1.35) |
| N-Acetyl-S-(2,2-dichlorovinyl)-L-cysteine [2DCV] |  | -----^b^ | -----^b^ | -----^b^ |
| N-Acetyl-S-(3-hydroxypropyl-1-methyl)-L-cysteine [HPMM] |  | 1.10 (0.73, 1.64) | 1.23 (1.01, 1.49) | 1.02 (0.85, 1.22) |
| N-Acetyl-S-(3-hydroxypropyl)-L-cysteine [HPMA] |  | 1.30 (0.96, 1.75) | 1.27 (1.01, 1.59) | 0.99 (0.81, 1.21) |
| N-Acetyl-S-(3,4-dihydroxybutyl)-L-cysteine [DHBM] |  | 1.07 (0.90, 1.27) | 1.08 (0.93, 1.26) | 1.01 (0.92, 1.10) |
| N-Acetyl-S-(4-hydroxy-2-butenyl)-L-cysteine [MHB3] |  | 1.08 (0.75, 1.56) | 1.16 (0.92, 1.46) | 0.95 (0.78, 1.17) |
| N-Acetyl-S-(benzyl)-L-cysteine [BMA] |  | 1.01 (0.74, 1.38) | 0.97 (0.69, 1.35) | 1.41 (1.02, 1.97) |
| N-Acetyl-S-(dimethylphenyl)-L-cysteine [DPMA] |  | -----^b^ | -----^b^ | -----^b^ |
| N-Acetyl-S-(n-methylcarbamoyl)-L-cysteine [AMCA] |  | 0.82 (0.58, 1.17) | 1.04 (0.82, 1.30) | 1.03 (0.94, 1.12) |
| N-Acetyl-S-(n-propyl)-L-cysteine [BPMA] |  | 1.36 (0.62, 3.01) | 0.85 (0.56, 1.30) | 0.92 (0.64, 1.33) |
| N-Acetyl-S-(phenyl-2-hydroxyethyl)-L-cysteine [PHEM] |  | -----^b^ | 0.86 (0.46, 1.58) | 1.11 (0.74, 1.67) |
| N-Acetyl-S-(phenyl)-L-cysteine [PMA] |  | 1.16 (0.80, 1.67) | 0.85 (0.61, 1.18) | 0.99 (0.75, 1.30) |
| N-Acetyl-S-(trichlorovinyl)-L-cysteine [TCVM] |  | -----^b^ | -----^b^ | -----^b^ |
| Phenylglyoxylic acid [PHGA] |  | 1.03 (0.80, 1.33) | 1.04 (0.73, 1.47) | 1.07 (0.92, 1.25) |
|  |  |  |  |  |
|  |  |  |  |  |
| Abbreviations: ANA = antinuclear antibodies; CI = confidence interval; MCR = mean concentration ratio. | | | | |
| Note: green highlighting indicates 0.01 < P-value ≤ 0.05; yellow highlighting indicates 0.001 < P-value ≤ 0.01; and red highlighting indicates P-value ≤ 0.001. | | | | |
| ^a^ The MCR is the ratio of mean concentrations for ANA-positive versus ANA-negative participants under a lognormal model for xenobiotic concentration; it is adjusted for sex, age, elderly status, race/ethnicity, BMI, PIR, smoking, birthplace, and cycle, but not for the sampling weights. The null MCR value is 1; MCR>1 indicates a positive association between ANA and xenobiotic concentration; and MCR<1 indicates a negative association between ANA and xenobiotic concentration. The CIs are based on variance estimates that make jackknife adjustments for correlations induced by the strata and clusters. | | | | |
| ^b^ No estimates are shown if <5 ANA-positive participants had detectable concentrations or if >90% of all concentrations were below the limit of detection. | | | | |

| Supplemental Table 5e. Covariate-adjusted estimates of ANA associations with metals and metalloids, for males stratified by age. | | | | |
| --- | --- | --- | --- | --- |
|  |  |  |  |  |
|  |  |  |  |  |
|  |  | Estimated MCR (and 95% CI) for Assessing Association between ANA and Xenobiotic ^a^ | | |
| Xenobiotic Name |  | Males, Ages 12-19 years | Males, Ages 20-49 years | Males, Ages ≥50 years |
|  |  |  |  |  |
| Antimony |  | 0.89 (0.74, 1.08) | 0.82 (0.64, 1.03) | 1.24 (0.98, 1.58) |
| Arsenic, total |  | 0.77 (0.50, 1.18) | 1.00 (0.72, 1.41) | 0.94 (0.77, 1.16) |
| Arsenic (V) acid |  | -----^b^ | -----^b^ | -----^b^ |
| Arsenobetaine |  | 1.10 (0.16, 7.69) | 1.56 (0.82, 2.97) | 0.84 (0.47, 1.50) |
| Arsenocholine |  | -----^b^ | -----^b^ | -----^b^ |
| Arsenous (III) acid |  | 0.76 (0.50, 1.14) | 0.91 (0.60, 1.39) | 0.90 (0.64, 1.26) |
| Dimethylarsinic acid |  | 0.82 (0.62, 1.10) | 0.91 (0.73, 1.13) | 1.16 (0.94, 1.43) |
| Monomethylarsonic acid |  | -----^b^ | 0.98 (0.71, 1.35) | 1.08 (0.86, 1.34) |
| Trimethylarsine oxide |  | -----^b^ | -----^b^ | -----^b^ |
| Barium |  | 0.75 (0.49, 1.15) | 1.02 (0.66, 1.56) | 0.88 (0.67, 1.15) |
| Cadmium, blood |  | 0.98 (0.82, 1.18) | 0.98 (0.87, 1.10) | 1.02 (0.93, 1.12) |
| Cadmium, urinary |  | 1.38 (0.88, 2.19) | 1.02 (0.85, 1.22) | 1.14 (1.04, 1.25) |
| Cesium |  | 1.00 (0.81, 1.25) | 1.04 (0.90, 1.20) | 0.99 (0.87, 1.12) |
| Cobalt |  | 0.92 (0.72, 1.19) | 1.09 (0.91, 1.31) | 0.95 (0.79, 1.14) |
| Copper |  | 0.97 (0.89, 1.05) | 0.94 (0.85, 1.05) | 1.00 (0.95, 1.06) |
| Lead, blood |  | 0.93 (0.81, 1.06) | 1.04 (0.95, 1.14) | 1.01 (0.94, 1.09) |
| Lead, urinary |  | 0.77 (0.57, 1.04) | 1.01 (0.86, 1.20) | 1.08 (0.91, 1.30) |
| Manganese, blood |  | 0.96 (0.87, 1.05) | 0.97 (0.89, 1.05) | 0.99 (0.93, 1.05) |
| Manganese, urinary |  | 0.83 (0.53, 1.28) | 1.03 (0.71, 1.48) | 0.98 (0.78, 1.22) |
| Mercury, total |  | 1.20 (0.94, 1.52) | 0.98 (0.77, 1.23) | 1.03 (0.86, 1.24) |
| Mercury, ethyl |  | -----^b^ | -----^b^ | -----^b^ |
| Mercury, inorganic |  | 1.18 (0.85, 1.63) | 0.94 (0.78, 1.13) | 1.16 (0.98, 1.37) |
| Mercury, methyl |  | 1.38 (0.91, 2.09) | 1.09 (0.81, 1.45) | 1.05 (0.83, 1.33) |
| Mercury, urinary |  | 1.10 (0.73, 1.64) | 1.10 (0.83, 1.47) | 0.98 (0.67, 1.42) |
| Molybdenum |  | 0.89 (0.65, 1.21) | 0.90 (0.69, 1.18) | 0.94 (0.82, 1.08) |
| Selenium, blood |  | 0.98 (0.94, 1.02) | 1.00 (0.98, 1.03) | 0.98 (0.95, 1.01) |
| Selenium, serum |  | 1.02 (0.98, 1.06) | 0.98 (0.95, 1.01) | 0.99 (0.96, 1.02) |
| Strontium |  | 0.94 (0.70, 1.26) | 0.99 (0.74, 1.31) | 0.96 (0.79, 1.16) |
| Thallium |  | 1.00 (0.81, 1.24) | 0.97 (0.84, 1.11) | 1.04 (0.85, 1.27) |
| Tin |  | 1.07 (0.60, 1.89) | 0.75 (0.55, 1.04) | 1.27 (1.00, 1.61) |
| Tungsten |  | 1.13 (0.80, 1.61) | 1.05 (0.81, 1.35) | 0.81 (0.62, 1.06) |
| Uranium |  | 0.93 (0.59, 1.46) | 1.16 (0.76, 1.76) | 1.20 (0.90, 1.59) |
| Zinc |  | 1.00 (0.94, 1.06) | 1.02 (0.95, 1.10) | 0.97 (0.91, 1.03) |
|  |  |  |  |  |
|  |  |  |  |  |
| Abbreviations: ANA = antinuclear antibodies; CI = confidence interval; MCR = mean concentration ratio. | | | | |
| Note: green highlighting indicates 0.01 < P-value ≤ 0.05; yellow highlighting indicates 0.001 < P-value ≤ 0.01; and red highlighting indicates P-value ≤ 0.001. | | | | |
| ^a^ The MCR is the ratio of mean concentrations for ANA-positive versus ANA-negative participants under a lognormal model for xenobiotic concentration; it is adjusted for sex, age, elderly status, race/ethnicity, BMI, PIR, smoking, birthplace, and cycle, but not for the sampling weights. The null MCR value is 1; MCR>1 indicates a positive association between ANA and xenobiotic concentration; and MCR<1 indicates a negative association between ANA and xenobiotic concentration. The CIs are based on variance estimates that make jackknife adjustments for correlations induced by the strata and clusters. | | | | |
| ^b^ No estimates are shown if <5 ANA-positive participants had detectable concentrations or if >90% of all concentrations were below the limit of detection. | | | | |

| Supplemental Table 5f. Covariate-adjusted estimates of ANA associations with metabolites of phthalates, phthalate alternatives, and polycyclic aromatic hydrocarbons, for males stratified by age. | | | | | |
| --- | --- | --- | --- | --- | --- |
|  |  |  |  |  |  |
|  |  |  |  |  |  |
| Xenobiotic Class | |  | Estimated MCR (and 95% CI) for Assessing Association between ANA and Xenobiotic ^a^ | | |
|  | Xenobiotic Name [Alternative/Abbreviated Name] |  | Males, Ages 12-19 years | Males, Ages 20-49 years | Males, Ages ≥50 years |
|  |  |  |  |  |  |
| Phthalate and Phthalate Alternative Metabolites | |  |  |  |  |
|  | Cyclohexane 1,2-dicarboxylic acid monohydroxy isononyl ester [MHNCH] |  | 1.36 (0.84, 2.20) | -----^b^ | 0.85 (0.50, 1.46) |
|  | Mono-(2-ethyl-5-hydroxyhexyl) phthalate [MEHPP] |  | 0.96 (0.54, 1.72) | 1.23 (0.75, 2.02) | 0.79 (0.54, 1.15) |
|  | Mono-(2-ethyl-5-oxohexyl) phthalate [MEOHP] |  | 0.97 (0.57, 1.65) | 1.08 (0.67, 1.75) | 0.83 (0.59, 1.18) |
|  | Mono-(2-ethyl)-hexyl phthalate [MEHP] |  | 1.08 (0.52, 2.25) | 1.00 (0.53, 1.89) | 0.87 (0.56, 1.35) |
|  | Mono-(3-carboxypropyl) phthalate [MCPP] |  | 1.40 (0.66, 2.98) | 0.76 (0.39, 1.47) | 1.07 (0.68, 1.67) |
|  | Mono-2-ethyl-5-carboxypentyl phthalate [MECPP] |  | 0.97 (0.59, 1.62) | 1.13 (0.68, 1.88) | 0.87 (0.62, 1.23) |
|  | Mono-benzyl phthalate [MBzP] |  | 0.94 (0.56, 1.59) | 0.62 (0.40, 0.97) | 1.06 (0.70, 1.61) |
|  | Mono-ethyl phthalate [MEP] |  | 0.85 (0.38, 1.89) | 1.36 (0.79, 2.33) | 0.84 (0.59, 1.21) |
|  | Mono-isobutyl phthalate [MiBP] |  | 0.86 (0.58, 1.29) | 0.78 (0.49, 1.26) | 0.86 (0.56, 1.31) |
|  | Mono-isononyl phthalate [MiNP] |  | 2.02 (0.60, 6.79) | 0.67 (0.37, 1.24) | 1.15 (0.56, 2.39) |
|  | Mono-n-butyl phthalate [MnBP] |  | 0.55 (0.32, 0.95) | 0.75 (0.48, 1.16) | 0.91 (0.52, 1.61) |
|  | Mono-n-methyl phthalate [MMP] |  | 1.21 (0.41, 3.52) | 0.96 (0.64, 1.44) | 0.75 (0.36, 1.54) |
|  | Mono(carboxynonyl) phthalate [MCNP] |  | 1.08 (0.73, 1.62) | 0.74 (0.58, 0.96) | 1.28 (0.91, 1.79) |
|  | Mono(carboxyoctyl) phthalate [MCOP] |  | 1.14 (0.62, 2.08) | 0.78 (0.49, 1.24) | 1.17 (0.69, 1.98) |
|  |  |  |  |  |  |
| Polycyclic Aromatic Hydrocarbon Metabolites | |  |  |  |  |
|  | 1-Hydroxynaphthalene [1-Naphthol] |  | 0.75 (0.46, 1.25) | 1.51 (1.10, 2.09) | 1.19 (0.97, 1.46) |
|  | 1-Hydroxyphenanthrene |  | 0.90 (0.64, 1.25) | 1.05 (0.83, 1.32) | 1.03 (0.84, 1.27) |
|  | 1-Hydroxypyrene |  | 0.86 (0.54, 1.36) | 0.97 (0.72, 1.31) | 1.06 (0.90, 1.24) |
|  | 2-Hydroxyfluorene |  | 0.94 (0.61, 1.43) | 1.16 (0.96, 1.40) | 1.10 (0.91, 1.32) |
|  | 2-Hydroxynaphthalene [2-Naphthol] |  | 1.26 (0.80, 2.00) | 1.13 (0.93, 1.36) | 1.14 (0.97, 1.35) |
|  | 2-Hydroxyphenanthrene |  | 0.89 (0.57, 1.37) | 1.01 (0.78, 1.31) | 1.04 (0.87, 1.23) |
|  | 3-Hydroxyfluorene |  | 0.96 (0.62, 1.48) | 1.31 (1.06, 1.63) | 1.12 (0.87, 1.46) |
|  | 3-Hydroxyphenanthrene |  | 0.84 (0.53, 1.32) | 1.10 (0.84, 1.42) | 1.10 (0.91, 1.33) |
|  | 4-Hydroxyphenanthrene |  | 0.94 (0.57, 1.55) | 1.12 (0.84, 1.50) | 1.10 (0.88, 1.39) |
|  | 9-Hydroxyfluorene |  | 0.91 (0.63, 1.32) | 1.11 (0.81, 1.53) | 1.11 (0.89, 1.38) |
|  |  |  |  |  |  |
|  |  |  |  |  |  |
| Abbreviations: ANA = antinuclear antibodies; CI = confidence interval; MCR = mean concentration ratio. | | | | | |
| Note: green highlighting indicates 0.01 < P-value ≤ 0.05; yellow highlighting indicates 0.001 < P-value ≤ 0.01; and red highlighting indicates P-value ≤ 0.001. | | | | | |
| ^a^ The MCR is the ratio of mean concentrations for ANA-positive versus ANA-negative participants under a lognormal model for xenobiotic concentration; it is adjusted for sex, age, elderly status, race/ethnicity, BMI, PIR, smoking, birthplace, and cycle, but not for the sampling weights. The null MCR value is 1; MCR>1 indicates a positive association between ANA and xenobiotic concentration; and MCR<1 indicates a negative association between ANA and xenobiotic concentration. The CIs are based on variance estimates that make jackknife adjustments for correlations induced by the strata and clusters. | | | | | |
| ^b^ No estimates are shown if <5 ANA-positive participants had detectable concentrations or if >90% of all concentrations were below the limit of detection. | | | | | |

| Supplemental Table 5g. Covariate-adjusted estimates of ANA associations with pesticides and their metabolites, for males stratified by age. | | | | | |
| --- | --- | --- | --- | --- | --- |
|  |  |  |  |  |  |
|  |  |  |  |  |  |
| Xenobiotic Class | |  | Estimated MCR (and 95% CI) for Assessing Association between ANA and Xenobiotic ^a^ | | |
|  | Xenobiotic Name [Alternative/Abbreviated Name] |  | Males, Ages 12-19 years | Males, Ages 20-49 years | Males, Ages ≥50 years |
|  |  |  |  |  |  |
| Organochlorine Pesticides and Metabolites | |  |  |  |  |
|  | 2,4,5-Trichlorophenol [2,4,5-TCP] |  | 0.92 (0.50, 1.70) | 1.27 (0.91, 1.79) | 1.14 (0.75, 1.74) |
|  | 2,4,6-Trichlorophenol |  | 1.02 (0.61, 1.72) | 1.20 (0.75, 1.93) | 1.45 (1.01, 2.10) |
|  | Aldrin |  | -----^b^ | -----^b^ | -----^b^ |
|  | beta-Hexachlorocyclohexane |  | -----^b^ | 1.03 (0.76, 1.40) | 0.98 (0.74, 1.29) |
|  | Dieldrin |  | -----^b^ | 0.95 (0.69, 1.32) | 0.93 (0.68, 1.27) |
|  | Endrin |  | -----^b^ | -----^b^ | -----^b^ |
|  | gamma-Hexachlorocyclohexane |  | -----^b^ | -----^b^ | -----^b^ |
|  | Heptachlor epoxide |  | -----^b^ | 1.00 (0.78, 1.27) | 1.05 (0.82, 1.35) |
|  | Hexachlorobenzene |  | -----^b^ | -----^b^ | -----^b^ |
|  | Mirex |  | -----^b^ | 1.11 (0.56, 2.21) | 1.22 (0.76, 1.95) |
|  | Oxychlordane |  | -----^b^ | 1.04 (0.80, 1.34) | 1.13 (0.92, 1.38) |
|  | o,p'-Dichlorodiphenyltrichloro ethane [o,p'-DDT] |  | -----^b^ | -----^b^ | -----^b^ |
|  | p,p'-Dichlorodiphenyltrichloro ethane [p,p'-DDT] |  | -----^b^ | 1.50 (0.67, 3.36) | 1.20 (0.88, 1.64) |
|  | p,p'-Dichlorodiphenyltrichloro ethylene [p,p'-DDE] |  | 0.88 (0.48, 1.62) | 1.07 (0.79, 1.45) | 1.04 (0.80, 1.34) |
|  | trans-Nonachlor |  | -----^b^ | 1.09 (0.78, 1.52) | 1.22 (1.02, 1.45) |
|  |  |  |  |  |  |
| Carbamate Pesticide Metabolites | |  |  |  |  |
|  | 2-Isopropoxyphenol |  | -----^b^ | -----^b^ | -----^b^ |
|  | Carbofuranphenol |  | -----^b^ | -----^b^ | -----^b^ |
|  |  |  |  |  |  |
|  |  |  |  |  |  |
| Abbreviations: ANA = antinuclear antibodies; CI = confidence interval; MCR = mean concentration ratio. | | | | | |
| Note: green highlighting indicates 0.01 < P-value ≤ 0.05; yellow highlighting indicates 0.001 < P-value ≤ 0.01; and red highlighting indicates P-value ≤ 0.001. | | | | | |
| ^a^ The MCR is the ratio of mean concentrations for ANA-positive versus ANA-negative participants under a lognormal model for xenobiotic concentration; it is adjusted for sex, age, elderly status, race/ethnicity, BMI, PIR, smoking, birthplace, and cycle, but not for the sampling weights. The null MCR value is 1; MCR>1 indicates a positive association between ANA and xenobiotic concentration; and MCR<1 indicates a negative association between ANA and xenobiotic concentration. The CIs are based on variance estimates that make jackknife adjustments for correlations induced by the strata and clusters. | | | | | |
| ^b^ No estimates are shown if <5 ANA-positive participants had detectable concentrations or if >90% of all concentrations were below the limit of detection. | | | | | |

| Supplemental Table 5h. Covariate-adjusted estimates of ANA associations with herbicides, fungicides, insecticides, and their metabolites, for males stratified by age. | | | | | |
| --- | --- | --- | --- | --- | --- |
|  |  |  |  |  |  |
|  |  |  |  |  |  |
| Xenobiotic Class | |  | Estimated MCR (and 95% CI) for Assessing Association between ANA and Xenobiotic ^a^ | | |
|  | Xenobiotic Name [Alternative/Abbreviated Name] |  | Males, Ages 12-19 years | Males, Ages 20-49 years | Males, Ages ≥50 years |
|  |  |  |  |  |  |
| Herbicides and Metabolites | |  |  |  |  |
|  | 2,4-Dichlorophenoxyacetic acid [2,4-D] |  | 0.83 (0.53, 1.30) | 0.83 (0.49, 1.41) | 0.91 (0.66, 1.25) |
|  | 2,4,5-Trichlorophenoxyacetic acid [2,4,5-T] |  | -----^b^ | -----^b^ | -----^b^ |
|  | Acetochlor mercapturate |  | -----^b^ | -----^b^ | -----^b^ |
|  | Alachlor mercapturate |  | -----^b^ | -----^b^ | -----^b^ |
|  | Atrazine mercapturate |  | -----^b^ | -----^b^ | -----^b^ |
|  | Metolachlor mercapturate |  | -----^b^ | -----^b^ | -----^b^ |
|  |  |  |  |  |  |
| Fungicides and Metabolites | |  |  |  |  |
|  | Pentachlorophenol |  | 1.06 (0.42, 2.66) | 2.81 (0.78, 10.10) | 0.34 (0.15, 0.75) |
|  | ortho-Phenylphenol |  | 0.88 (0.47, 1.67) | 1.04 (0.41, 2.68) | 0.80 (0.42, 1.53) |
|  |  |  |  |  |  |
| Pyrethroid Insecticide Metabolites | |  |  |  |  |
|  | 3-Phenoxybenzoic acid [3PBA] |  | 0.60 (0.31, 1.13) | 1.32 (0.90, 1.93) | 0.81 (0.55, 1.19) |
|  | 4-Fluoro-3-phenoxybenzoic acid [4F3PBA] |  | -----^b^ | -----^b^ | -----^b^ |
|  | Cis-3-(2,2-dibromovinyl)-2,2-dimethyl cyclopropane carboxylic acid [cis-DBCA] |  | -----^b^ | -----^b^ | -----^b^ |
|  | Cis-3-(2,2-dichlorovinyl)-2,2-dimethyl cyclopropane carboxylic acid [cis-DCCA] |  | -----^b^ | 0.90 (0.34, 2.36) | 1.08 (0.45, 2.55) |
|  | trans-3-(2,2-Dichlorovinyl)-2,2-dimethyl cyclopropane carboxylic acid [trans-DCCA] |  | -----^b^ | 0.88 (0.34, 2.24) | 0.58 (0.18, 1.86) |
|  |  |  |  |  |  |
| Organophosphorus Insecticides: Dialkyl Phosphate Metabolites | |  |  |  |  |
|  | Diethyldithiophosphate [DEDTP] |  | 0.70 (0.23, 2.15) | 0.42 (0.14, 1.25) | 0.92 (0.43, 1.96) |
|  | Diethylphosphate [DEP] |  | 0.94 (0.32, 2.77) | 1.24 (0.69, 2.22) | 1.26 (0.75, 2.13) |
|  | Diethylthiophosphate [DETP] |  | 0.79 (0.41, 1.51) | 1.13 (0.69, 1.87) | 1.22 (0.87, 1.72) |
|  | Dimethyldithiophosphate [DMDTP] |  | 1.48 (0.62, 3.55) | 0.41 (0.17, 0.97) | 1.21 (0.65, 2.23) |
|  | Dimethylphosphate [DMP] |  | 0.78 (0.39, 1.55) | 0.91 (0.57, 1.44) | 1.19 (0.81, 1.76) |
|  | Dimethylthiophosphate [DMTP] |  | 0.88 (0.41, 1.88) | 0.78 (0.47, 1.28) | 0.94 (0.61, 1.44) |
|  |  |  |  |  |  |
| Organophosphorus Insecticides: Specific Pesticides & Metabolites | | |  |  |  |
|  | 2-(Diethylamino)-6-methylpyrimidin-4-ol/one |  | -----^b^ | -----^b^ | -----^b^ |
|  | 2-Isopropyl-4-methyl-6-hydroxy pyrimidine [Oxypyrimidine] |  | 0.73 (0.32, 1.71) | 2.14 (0.82, 5.55) | 1.32 (0.65, 2.67) |
|  | 3-Chloro-7-hydroxy-4-methyl-2H-chromen-2-one/ol |  | -----^b^ | -----^b^ | -----^b^ |
|  | 3,5,6-Trichloro-2-pyridinol [TCPy] |  | 1.03 (0.62, 1.74) | 0.94 (0.63, 1.40) | 1.00 (0.56, 1.77) |
|  | Malathion dicarboxylic acid |  | -----^b^ | -----^b^ | -----^b^ |
|  | para-Nitrophenol |  | 0.62 (0.35, 1.10) | 1.40 (0.81, 2.44) | 1.08 (0.72, 1.62) |
|  |  |  |  |  |  |
| Insect Repellents and Metabolites | |  |  |  |  |
|  | 3-Diethylcarbamoylbenzoic acid [DCBA or DEET acid] |  | 1.34 (0.48, 3.77) | 1.38 (0.63, 3.01) | 1.63 (0.92, 2.88) |
|  | N,N-Diethyl-3-(hydroxymethyl) benzamide [DHMB] |  | -----^b^ | -----^b^ | -----^b^ |
|  | N,N-Diethyl-meta-toluamide [DEET] |  | -----^b^ | -----^b^ | -----^b^ |
|  |  |  |  |  |  |
|  |  |  |  |  |  |
| Abbreviations: ANA = antinuclear antibodies; CI = confidence interval; MCR = mean concentration ratio. | | | | | |
| Note: green highlighting indicates 0.01 < P-value ≤ 0.05; yellow highlighting indicates 0.001 < P-value ≤ 0.01; and red highlighting indicates P-value ≤ 0.001. | | | | | |
| ^a^ The MCR is the ratio of mean concentrations for ANA-positive versus ANA-negative participants under a lognormal model for xenobiotic concentration; it is adjusted for sex, age, elderly status, race/ethnicity, BMI, PIR, smoking, birthplace, and cycle, but not for the sampling weights. The null MCR value is 1; MCR>1 indicates a positive association between ANA and xenobiotic concentration; and MCR<1 indicates a negative association between ANA and xenobiotic concentration. The CIs are based on variance estimates that make jackknife adjustments for correlations induced by the strata and clusters. | | | | | |
| ^b^ No estimates are shown if <5 ANA-positive participants had detectable concentrations or if >90% of all concentrations were below the limit of detection. | | | | | |

| Supplemental Table 5i. Covariate-adjusted estimates of ANA associations with perfluoroalkyl and polyfluoroalkyl substances, perchlorate and other anions, personal care and consumer product chemicals and metabolites, and tobacco biomarkers, for males stratified by age. | | | | | |
| --- | --- | --- | --- | --- | --- |
|  |  |  |  |  |  |
|  |  |  |  |  |  |
| Xenobiotic Class | |  | Estimated MCR (and 95% CI) for Assessing Association between ANA and Xenobiotic ^a^ | | |
|  | Xenobiotic Name [Alternative/Abbreviated Name] |  | Males, Ages 12-19 years | Males, Ages 20-49 years | Males, Ages ≥50 years |
|  |  |  |  |  |  |
| Perfluoroalkyl and Polyfluoroalkyl Substances [PFAS] | |  |  |  |  |
|  | 2-(N-ethyl-perfluorooctane sulfonamido) acetic acid [EtFOSAA] |  | -----^b^ | -----^b^ | -----^b^ |
|  | 2-(N-methyl-perfluorooctane sulfonamido) acetic acid [MeFOSAA] |  | 0.51 (0.33, 0.78) | 1.10 (0.57, 2.15) | 1.11 (0.68, 1.81) |
|  | Perfluorodecanoic acid [PFDA] |  | 0.93 (0.70, 1.24) | 1.00 (0.72, 1.39) | 0.92 (0.66, 1.29) |
|  | Perfluorobutane sulfonic acid [PFBS] |  | -----^b^ | -----^b^ | -----^b^ |
|  | Perfluoroheptanoic acid [PFHpA] |  | -----^b^ | -----^b^ | 1.32 (1.04, 1.67) |
|  | Perfluorohexane sulfonic acid [PFHxS] |  | 1.20 (0.78, 1.87) | 1.04 (0.80, 1.36) | 0.82 (0.56, 1.21) |
|  | Perfluorononanoic acid [PFNA] |  | 1.26 (0.91, 1.76) | 1.06 (0.78, 1.45) | 0.91 (0.67, 1.23) |
|  | Perfluorooctane sulfonamide [PFOSA] |  | -----^b^ | -----^b^ | -----^b^ |
|  | Perfluorooctane sulfonic acid [PFOS] |  | 1.02 (0.81, 1.28) | 1.20 (0.94, 1.52) | 0.72 (0.49, 1.06) |
|  | Perfluorooctanoic acid [PFOA] |  | 1.06 (0.87, 1.29) | 1.06 (0.83, 1.36) | 0.95 (0.72, 1.24) |
|  | Perfluoroundecanoic acid [PFUA] |  | 0.85 (0.46, 1.58) | 1.17 (0.81, 1.68) | 1.08 (0.72, 1.62) |
|  | Perflurododecanoic acid [PFDoH] |  | -----^b^ | -----^b^ | 1.99 (1.17, 3.40) |
|  |  |  |  |  |  |
| Perchlorate and Other Anions | |  |  |  |  |
|  | Nitrate |  | 1.05 (0.77, 1.44) | 0.95 (0.82, 1.10) | 0.92 (0.80, 1.06) |
|  | Perchlorate |  | 1.04 (0.76, 1.41) | 0.84 (0.69, 1.02) | 1.01 (0.87, 1.18) |
|  | Thiocyanate |  | 1.04 (0.62, 1.76) | 1.13 (0.92, 1.38) | 1.10 (0.89, 1.38) |
|  |  |  |  |  |  |
| Personal Care and Consumer Product Chemicals and Metabolites | |  |  |  |  |
|  | 2,4-Dichlorophenol |  | 0.79 (0.50, 1.23) | 0.85 (0.57, 1.26) | 1.32 (0.89, 1.95) |
|  | 2,5-Dichlorophenol |  | 1.03 (0.58, 1.83) | 0.92 (0.49, 1.74) | 1.53 (0.94, 2.50) |
|  | Benzophenone-3 |  | 0.81 (0.52, 1.27) | 0.85 (0.50, 1.45) | 0.92 (0.64, 1.32) |
|  | Bisphenol A |  | 0.91 (0.69, 1.21) | 0.83 (0.67, 1.04) | 1.06 (0.82, 1.36) |
|  | Butyl paraben |  | -----^b^ | -----^b^ | -----^b^ |
|  | Ethyl paraben |  | 1.68 (0.33, 8.63) | 1.21 (0.41, 3.53) | 1.39 (0.60, 3.21) |
|  | Methyl paraben |  | 0.98 (0.33, 2.95) | 0.80 (0.45, 1.43) | 0.77 (0.45, 1.35) |
|  | Propyl paraben [n-Propyl paraben] |  | 1.39 (0.36, 5.28) | 0.94 (0.55, 1.61) | 0.51 (0.17, 1.53) |
|  | Triclosan |  | 0.62 (0.27, 1.42) | 0.98 (0.45, 2.14) | 1.29 (0.73, 2.27) |
|  |  |  |  |  |  |
| Tobacco Alkaloids and Metabolites ^c^ | |  |  |  |  |
|  | Cotinine |  | 0.65 (0.30, 1.40) | 0.97 (0.51, 1.84) | 1.21 (0.70, 2.09) |
|  | 4-(Methylnitrosamino)-1-(3-pyridyl)-1-butanol [NNAL] |  | 0.99 (0.52, 1.89) | 0.82 (0.37, 1.82) | 2.25 (1.09, 4.64) |
|  |  |  |  |  |  |
|  |  |  |  |  |  |
| Abbreviations: ANA = antinuclear antibodies; CI = confidence interval; MCR = mean concentration ratio. | | | | | |
| Note: green highlighting indicates 0.01 < P-value ≤ 0.05; yellow highlighting indicates 0.001 < P-value ≤ 0.01; and red highlighting indicates P-value ≤ 0.001. | | | | | |
| ^a^ The MCR is the ratio of mean concentrations for ANA-positive versus ANA-negative participants under a lognormal model for xenobiotic concentration; it is adjusted for sex, age, elderly status, race/ethnicity, BMI, PIR, smoking, birthplace, and cycle, but not for the sampling weights. The null MCR value is 1; MCR>1 indicates a positive association between ANA and xenobiotic concentration; and MCR<1 indicates a negative association between ANA and xenobiotic concentration. The CIs are based on variance estimates that make jackknife adjustments for correlations induced by the strata and clusters. | | | | | |
| ^b^ No estimates are shown if <5 ANA-positive participants had detectable concentrations or if >90% of all concentrations were below the limit of detection. | | | | | |
| ^c^ The smoking covariate was dropped from the model for tobacco biomarkers. | | | | | |
