# Supplementary material for: Expanded assessment of xenobiotic associations with antinuclear antibodies in the United States, 1988–2012
Source: Environ Int. Author manuscript; Available in PMC 2023 Oct 18. (PMC9792625; doi:10.1016/j.envint.2022.107376)
Supplement: MMC1 [file NIHMS1820607-supplement-MMC1.docx]

| Supplemental Table 1a. Summary information about dioxin-like compounds among NHANES participants with data on ANA. | | | | | | | | | | |
| --- | --- | --- | --- | --- | --- | --- | --- | --- | --- | --- |
|  |  |  |  |  |  |  |  |  |  |  |
|  |  |  |  |  |  |  |  |  |  |  |
|  |  |  |  | Number of Participants with Xenobiotic and ANA Data (Percent with Concentration <LOD) | | | | | | |
| Xenobiotic Class | |  |  | Cycle 1 ^a^ | Cycle 2 | Cycle 3 | Cycle 4 | Cycle 5 ^a^ |  | All Cycles |
|  | Xenobiotic Name [Alternative/Abbreviated Name] | TEF ^b^ |  | 1988-1991 | 1999-2000 | 2001-2002 | 2003-2004 | 2011-2012 |  | Combined |
|  |  |  |  |  |  |  |  |  |  |  |
| Polychlorinated Dibenzo-p-dioxins [PCDDs] ^c^ | |  |  |  |  |  |  |  |  |  |
|  | 2,3,7,8-Tetrachlorodibenzo-p-dioxin [2,3,7,8-TCDD] | 1 |  | 0 (---) | 1,578 (100%) | 1,192 ( 87%) | 1,757 ( 64%) | 0 (---) |  | 4,527 ( 82%) |
|  | 1,2,3,7,8-Pentachlorodibenzo-p-dioxin [1,2,3,7,8-PeCDD] | 1 |  | 0 (---) | 1,568 ( 88%) | 1,187 ( 66%) | 1,757 ( 47%) | 0 (---) |  | 4,512 ( 66%) |
|  | 1,2,3,4,7,8-Hexachlorodibenzo-p-dioxin [1,2,3,4,7,8-HxCDD] | 0.1 |  | 0 (---) | 0 (---) | 1,190 ( 66%) | 1,738 ( 75%) | 0 (---) |  | 2,928 ( 72%) |
|  | 1,2,3,6,7,8-Hexachlorodibenzo-p-dioxin [1,2,3,6,7,8-HxCDD] | 0.1 |  | 0 (---) | 1,538 ( 61%) | 1,186 ( 7%) | 1,747 ( 19%) | 0 (---) |  | 4,471 ( 30%) |
|  | 1,2,3,7,8,9-Hexachlorodibenzo-p-dioxin [1,2,3,7,8,9-HxCDD] | 0.1 |  | 0 (---) | 1,526 ( 87%) | 1,188 ( 59%) | 1,746 ( 72%) | 0 (---) |  | 4,460 ( 74%) |
|  | 1,2,3,4,6,7,8-Heptachlorodibenzo-p-dioxin [1,2,3,4,6,7,8-HpCDD] | 0.01 |  | 0 (---) | 1,531 ( 42%) | 1,170 ( 1%) | 1,751 ( 3%) | 0 (---) |  | 4,452 ( 16%) |
|  | 1,2,3,4,6,7,8,9-Octachlorodibenzo-p-dioxin [1,2,3,4,6,7,8,9-OCDD] | 0.0003 |  | 0 (---) | 1,557 ( 38%) | 1,128 ( 18%) | 1,728 ( 16%) | 0 (---) |  | 4,413 ( 24%) |
|  |  |  |  |  |  |  |  |  |  |  |
| Polychlorinated Dibenzofurans [PCDFs] ^c^ | |  |  |  |  |  |  |  |  |  |
|  | 2,3,4,7,8-Pentachlorodibenzofuran [2,3,4,7,8-PeCDF] | 0.3 |  | 0 (---) | 1,560 ( 62%) | 1,181 ( 34%) | 1,748 ( 35%) | 0 (---) |  | 4,489 ( 44%) |
|  | 2,3,7,8-Tetrachlorodibenzofuran [2,3,7,8-TCDF] | 0.1 |  | 0 (---) | 1,559 (100%) | 1,183 ( 99%) | 1,747 ( 97%) | 0 (---) |  | 4,489 ( 99%) |
|  | 1,2,3,4,7,8-Hexachlorodibenzofuran [1,2,3,4,7,8-HxCDF] | 0.1 |  | 0 (---) | 1,544 ( 63%) | 1,177 ( 18%) | 1,743 ( 40%) | 0 (---) |  | 4,464 ( 42%) |
|  | 1,2,3,6,7,8-Hexachlorodibenzofuran [1,2,3,6,7,8-HxCDF] | 0.1 |  | 0 (---) | 1,555 ( 80%) | 1,189 ( 30%) | 1,745 ( 51%) | 0 (---) |  | 4,489 ( 55%) |
|  | 1,2,3,7,8,9-Hexachlorodibenzofuran [1,2,3,7,8,9-HxCDF] | 0.1 |  | 0 (---) | 1,532 (100%) | 1,176 (100%) | 1,742 (100%) | 0 (---) |  | 4,450 (100%) |
|  | 2,3,4,6,7,8-Hexachlorodibenzofuran [2,3,4,6,7,8-HxCDF] | 0.1 |  | 0 (---) | 1,543 ( 98%) | 1,181 ( 89%) | 1,743 ( 95%) | 0 (---) |  | 4,467 ( 95%) |
|  | 1,2,3,7,8-Pentachlorodibenzofuran [1,2,3,7,8-PeCDF] | 0.03 |  | 0 (---) | 1,573 (100%) | 1,185 ( 99%) | 1,744 ( 98%) | 0 (---) |  | 4,502 ( 99%) |
|  | 1,2,3,4,6,7,8-Heptachlorodibenzofuran [1,2,3,4,6,7,8-HpCDF] | 0.01 |  | 0 (---) | 1,392 ( 56%) | 1,168 ( 10%) | 1,736 ( 10%) | 0 (---) |  | 4,296 ( 25%) |
|  | 1,2,3,4,7,8,9-Heptachlorodibenzofuran [1,2,3,4,7,8,9-HpCDF] | 0.01 |  | 0 (---) | 0 (---) | 1,173 (100%) | 1,731 ( 94%) | 0 (---) |  | 2,904 ( 97%) |
|  | 1,2,3,4,6,7,8,9-Octachlorodibenzofuran [1,2,3,4,6,7,8,9-OCDF] | 0.0003 |  | 0 (---) | 1,533 ( 99%) | 1,154 (100%) | 1,727 ( 73%) | 0 (---) |  | 4,414 ( 89%) |
|  |  |  |  |  |  |  |  |  |  |  |
| Non-Ortho Dioxin-Like Polychlorinated Biphenyls [NODL PCBs] ^c^ | |  |  |  |  |  |  |  |  |  |
|  | 3,3',4,4',5-Pentachlorobiphenyl [PCB 126] | 0.1 |  | 0 (---) | 1,560 ( 51%) | 1,178 ( 12%) | 1,738 ( 7%) | 0 (---) |  | 4,476 ( 23%) |
|  | 3,3',4,4',5,5'-Hexachlorobiphenyl [PCB 169] | 0.03 |  | 0 (---) | 1,543 ( 52%) | 1,175 ( 12%) | 1,742 ( 42%) | 0 (---) |  | 4,460 ( 38%) |
|  | 3,4,4',5-Tetrachlorobiphenyl [PCB 81] | 0.0003 |  | 0 (---) | 1,541 ( 99%) | 1,167 (100%) | 1,738 ( 63%) | 0 (---) |  | 4,446 ( 85%) |
|  |  |  |  |  |  |  |  |  |  |  |
| Mono-Ortho Dioxin-Like Polychlorinated Biphenyls [MODL PCBs] ^d^ | |  |  |  |  |  |  |  |  |  |
|  | 2,3,3',4,4'-Pentachlorobiphenyl [PCB 105] | 0.00003 |  | 0 (---) | 1,523 ( 90%) | 1,192 ( 77%) | 1,707 ( 2%) | 0 (---) |  | 4,422 ( 53%) |
|  | 2,3',4,4',5-Pentachlorobiphenyl [PCB 118] | 0.00003 |  | 0 (---) | 1,534 ( 61%) | 1,192 ( 26%) | 1,713 ( 0%) | 0 (---) |  | 4,439 ( 28%) |
|  | 2,3,3',4,4',5-Hexachlorobiphenyl [PCB 156] | 0.00003 |  | 0 (---) | 1,517 ( 73%) | 1,187 ( 44%) | 1,716 ( 17%) | 0 (---) |  | 4,420 ( 43%) |
|  | 2,3,3',4,4',5'-Hexachlorobiphenyl [PCB 157] | 0.00003 |  | 0 (---) | 1,514 ( 97%) | 1,186 ( 91%) | 1,701 ( 37%) | 0 (---) |  | 4,401 ( 72%) |
|  | 2,3',4,4',5,5'-Hexachlorobiphenyl [PCB 167] | 0.00003 |  | 0 (---) | 1,520 ( 95%) | 1,185 ( 88%) | 1,708 ( 42%) | 0 (---) |  | 4,413 ( 73%) |
|  | 2,3,3',4,4',5,5'-Heptachlorobiphenyl [PCB 189] | 0.00003 |  | 0 (---) | 0 (---) | 1,190 (100%) | 1,668 ( 76%) | 0 (---) |  | 2,858 ( 86%) |
|  |  |  |  |  |  |  |  |  |  |  |
|  |  |  |  |  |  |  |  |  |  |  |
| Abbreviations: ANA = antinuclear antibodies; LOD = limit of detection; NHANES = National Health and Nutrition Examination Survey; TEF = toxic equivalency factor. | | | | | | | | | | |
| ^a^ None of these dioxin-like compounds were measured in 1988-1991 or 2011-2012, but Cycles 1 and 5 are included in the table for completeness. | | | | | | | | | | |
| ^b^ The TEF values are the 2005 World Health Organization estimates (Van den Berg et al, 2006). | | | | | | | | | | |
| ^c^ All xenobiotics in this class were measured in serum and were lipid adjusted, with concentration units of pg/g lipid. | | | | | | | | | | |
| ^d^ All xenobiotics in this class were measured in serum and were lipid adjusted, with concentration units of ng/g lipid. | | | | | | | | | | |

| Supplemental Table 1b. Summary information about non-dioxin-like polychlorinated biphenyls among NHANES participants with data on ANA. | | | | | | | |
| --- | --- | --- | --- | --- | --- | --- | --- |
|  |  |  |  |  |  |  |  |
|  |  |  |  |  |  |  |  |
|  | Number of Participants with Xenobiotic and ANA Data (Percent with Concentration <LOD) | | | | | | |
|  | Cycle 1 ^a^ | Cycle 2 | Cycle 3 | Cycle 4 | Cycle 5 ^a^ |  | All Cycles |
| Xenobiotic Name [Alternative/Abbreviated Name] ^b^ | 1988-1991 | 1999-2000 | 2001-2002 | 2003-2004 | 2011-2012 |  | Combined |
|  |  |  |  |  |  |  |  |
| 2,4,4'-Trichlorobiphenyl [PCB 28] | 0 (---) | 1,479 (98%) | 0 (---) | 1,694 ( 0%) | 0 (---) |  | 3,173 (46%) |
| 2,2',3,5'-Tetrachlorobiphenyl [PCB 44] | 0 (---) | 0 (---) | 0 (---) | 1,716 ( 0%) | 0 (---) |  | 1,716 ( 0%) |
| 2,2',4,5'-Tetrachlorobiphenyl [PCB 49] | 0 (---) | 0 (---) | 0 (---) | 1,704 ( 1%) | 0 (---) |  | 1,704 ( 1%) |
| 2,2',5,5'-Tetrachlorobiphenyl [PCB 52] | 0 (---) | 1,523 (99%) | 980 ( 90%) | 1,723 ( 0%) | 0 (---) |  | 4,226 (57%) |
| 2,3',4,4'-Tetrachlorobiphenyl [PCB 66] | 0 (---) | 1,538 (97%) | 1,177 ( 89%) | 1,724 ( 1%) | 0 (---) |  | 4,439 (58%) |
| 2,4,4',5-Tetrachlorobiphenyl [PCB 74] | 0 (---) | 1,531 (63%) | 1,192 ( 31%) | 1,724 ( 0%) | 0 (---) |  | 4,447 (30%) |
| 2,2’,3,4,5’-Pentachlorobiphenyl [PCB 87] | 0 (---) | 0 (---) | 1,185 ( 99%) | 1,724 (17%) | 0 (---) |  | 2,909 (50%) |
| 2,2',4,4',5-Pentachlorobiphenyl [PCB 99] | 0 (---) | 1,513 (71%) | 1,176 ( 37%) | 1,706 ( 0%) | 0 (---) |  | 4,395 (34%) |
| 2,2',4,5,5'-Pentachlorobiphenyl [PCB 101] | 0 (---) | 1,536 (99%) | 1,192 ( 96%) | 1,724 ( 3%) | 0 (---) |  | 4,452 (61%) |
| 2,3,3’,4’,6-Pentachlorobiphenyl [PCB 110] | 0 (---) | 0 (---) | 1,185 ( 99%) | 1,710 ( 2%) | 0 (---) |  | 2,895 (41%) |
| 2,2',3,3',4,4'-Hexachlorobiphenyl [PCB 128] | 0 (---) | 1,540 (99%) | 1,185 (100%) | 1,722 (75%) | 0 (---) |  | 4,447 (90%) |
| 2,2',3,4,4',5'-Hexachlorobiphenyl [PCB 138] ^c^ | 0 (---) | 1,536 (66%) | 1,189 ( 6%) | 1,722 ( 0%) | 0 (---) |  | 4,447 (24%) |
| 2,2',3,4',5,5'-Hexachlorobiphenyl [PCB 146] | 0 (---) | 1,530 (77%) | 1,186 ( 50%) | 1,722 ( 2%) | 0 (---) |  | 4,438 (41%) |
| 2,2’,3,4’,5’,6-Hexachlorobiphenyl [PCB 149] | 0 (---) | 0 (---) | 1,192 (100%) | 1,701 ( 4%) | 0 (---) |  | 2,893 (44%) |
| 2,2’,3,5,5’,6-Hexachlorobiphenyl [PCB 151] | 0 (---) | 0 (---) | 1,192 ( 99%) | 1,702 (21%) | 0 (---) |  | 2,894 (53%) |
| 2,2',4,4',5,5'-Hexachlorobiphenyl [PCB 153] | 0 (---) | 1,533 (61%) | 1,192 ( 4%) | 1,722 ( 0%) | 0 (---) |  | 4,447 (22%) |
| 2,2',3,3',4,4',5-Heptachlorobiphenyl [PCB 170] | 0 (---) | 1,440 (63%) | 1,189 ( 23%) | 1,719 ( 3%) | 0 (---) |  | 4,348 (29%) |
| 2,2',3,3',4,5,5'-Heptachlorobiphenyl [PCB 172] | 0 (---) | 1,517 (97%) | 1,165 ( 82%) | 1,718 (35%) | 0 (---) |  | 4,400 (69%) |
| 2,2',3,3',4,5',6'-Heptachlorobiphenyl [PCB 177] | 0 (---) | 1,500 (94%) | 1,177 ( 81%) | 1,716 (20%) | 0 (---) |  | 4,393 (61%) |
| 2,2',3,3',5,5',6-Heptachlorobiphenyl [PCB 178] | 0 (---) | 1,538 (92%) | 1,186 ( 79%) | 1,722 (25%) | 0 (---) |  | 4,446 (62%) |
| 2,2',3,4,4',5,5'-Heptachlorobiphenyl [PCB 180] | 0 (---) | 1,535 (58%) | 1,190 ( 10%) | 1,723 ( 1%) | 0 (---) |  | 4,448 (23%) |
| 2,2',3,4,4',5',6-Heptachlorobiphenyl [PCB 183] | 0 (---) | 1,536 (87%) | 1,192 ( 67%) | 1,719 (11%) | 0 (---) |  | 4,447 (52%) |
| 2,2',3,4',5,5',6-Heptachlorobiphenyl [PCB 187] | 0 (---) | 1,535 (63%) | 1,192 ( 33%) | 1,717 ( 2%) | 0 (---) |  | 4,444 (31%) |
| 2,2’,3,3’,4,4’,5,5’-Octachlorobiphenyl [PCB 194] | 0 (---) | 0 (---) | 1,181 ( 37%) | 1,676 (22%) | 0 (---) |  | 2,857 (28%) |
| 2,2’,3,3’,4,4’,5,6-Octachlorobiphenyl [PCB 195] | 0 (---) | 0 (---) | 1,169 (100%) | 1,670 (46%) | 0 (---) |  | 2,839 (68%) |
| 2,2’,3,3’,4,4’,5,6’-Octachlorobiphenyl [PCB 196] ^d^ | 0 (---) | 0 (---) | 1,188 ( 43%) | 1,715 (13%) | 0 (---) |  | 2,903 (25%) |
| 2,2’,3,3’,4,5,5’,6-Octachlorobiphenyl [PCB 199] | 0 (---) | 0 (---) | 1,182 ( 40%) | 1,697 (14%) | 0 (---) |  | 2,879 (24%) |
| 2,2’,3,3’,4,4’,5,5’,6-Nonachlorobiphenyl [PCB 206] | 0 (---) | 0 (---) | 1,147 ( 88%) | 1,701 ( 7%) | 0 (---) |  | 2,848 (39%) |
| 2,2',3,3',4,4',5,5',6,6'-Decachlorobiphenyl [PCB 209] | 0 (---) | 0 (---) | 0 (---) | 1,688 ( 7%) | 0 (---) |  | 1,688 ( 7%) |
|  |  |  |  |  |  |  |  |
|  |  |  |  |  |  |  |  |
| Abbreviations: ANA = antinuclear antibodies; LOD = limit of detection; NHANES = National Health and Nutrition Examination Survey. | | | | | | | |
| ^a^ None of these non-dioxin-like PCBs were measured in 1988-1991 or 2011-2012, but Cycles 1 and 5 are included in the table for completeness. | | | | | | | |
| ^b^ All of these non-dioxin-like PCBs were measured in serum and were lipid adjusted, with concentration units of ng/g lipid. | | | | | | | |
| ^c^ The information in this row pertains to both 2,2',3,4,4',5'-Hexachlorobiphenyl [PCB 138] and 2,3,3’,4,4’,6-Hexachlorobiphenyl [PCB 158]. | | | | | | | |
| ^d^ The information in this row pertains to both 2,2’,3,3’,4,4’,5,6’-Octachlorobiphenyl [PCB 196] and 2,2’,3,4,4’,5,5’,6-Octachlorobiphenyl [PCB 203]. | | | | | | | |

| Supplemental Table 1c. Summary information about volatile organic compounds among NHANES participants with data on ANA. | | | | | | | |
| --- | --- | --- | --- | --- | --- | --- | --- |
|  |  |  |  |  |  |  |  |
|  |  |  |  |  |  |  |  |
|  | Number of Participants with Xenobiotic and ANA Data (Percent with Concentration <LOD) | | | | | | |
|  | Cycle 1 | Cycle 2 | Cycle 3 | Cycle 4 | Cycle 5 |  | All Cycles |
| Xenobiotic Name [Alternative/Abbreviated Name] ^a^ | 1988-1991 | 1999-2000 | 2001-2002 | 2003-2004 | 2011-2012 |  | Combined |
|  |  |  |  |  |  |  |  |
| 1,1-Dichloroethane | 267 ( 97%) | 0 (---) | 0 (---) | 785 (100%) | 2,168 (100%) |  | 3,220 (100%) |
| 1,1-Dichloroethene [Vinylidene chloride] | 256 ( 98%) | 0 (---) | 0 (---) | 785 (100%) | 2,228 (100%) |  | 3,269 (100%) |
| 1,1,1-Trichloroethane [Methyl chloroform] | 104 ( 24%) | 230 (96%) | 0 (--) | 769 (100%) | 2,110 ( 99%) |  | 3,213 ( 97%) |
| 1,1,1,2-Tetrachloroethane | 0 (---) | 0 (---) | 0 (---) | 0 (---) | 2,216 (100%) |  | 2,216 (100%) |
| 1,1,2-Trichloroethane | 259 (100%) | 0 (---) | 0 (---) | 777 (100%) | 2,217 (100%) |  | 3,253 (100%) |
| 1,1,2,2-Tetrachloroethane | 266 (100%) | 0 (---) | 0 (---) | 709 (100%) | 2,126 (100%) |  | 3,101 (100%) |
| 1,2-Dibromo-3-chloropropane [DBCP] | 0 (---) | 0 (---) | 0 (---) | 708 (100%) | 0 (---) |  | 708 (100%) |
| 1,2-Dibromoethane | 0 (---) | 0 (---) | 0 (---) | 0 (---) | 2,214 (100%) |  | 2,214 (100%) |
| 1,2-Dichlorobenzene [o-Dichlorobenzene] | 262 (100%) | 0 (---) | 0 (---) | 775 (100%) | 2,139 (100%) |  | 3,176 (100%) |
| 1,2-Dichloroethane [Ethylene dichloride] | 250 (100%) | 0 (---) | 0 (---) | 770 (100%) | 2,211 ( 98%) |  | 3,231 ( 99%) |
| 1,2-Dichloropropane | 219 ( 98%) | 0 (---) | 0 (---) | 782 (100%) | 2,170 (100%) |  | 3,171 (100%) |
| 1,2,3-Trichloropropane | 0 (---) | 0 (---) | 0 (---) | 0 (---) | 2,192 (100%) |  | 2,192 (100%) |
| 1,3-Dichlorobenzene | 255 ( 96%) | 0 (---) | 0 (---) | 765 (100%) | 2,186 ( 99%) |  | 3,206 ( 99%) |
| 1,4-Dichlorobenzene [Paradichlororbenzene] | 259 ( 2%) | 242 (11%) | 444 (47%) | 753 ( 48%) | 2,161 ( 43%) |  | 3,859 ( 39%) |
| 1,4-Dioxane | 0 (---) | 0 (---) | 0 (---) | 0 (---) | 2,219 (100%) |  | 2,219 (100%) |
| 2-Butanone | 264 ( 0%) | 0 (---) | 0 (---) | 0 (---) | 0 (---) |  | 264 ( 0%) |
| 2,5-Dimethylfuran | 0 (---) | 0 (---) | 0 (---) | 737 ( 75%) | 2,235 ( 83%) |  | 2,972 ( 81%) |
| Acetone | 237 ( 0%) | 0 (---) | 0 (---) | 0 (---) | 0 (---) |  | 237 ( 0%) |
| Benzene | 212 ( 18%) | 242 ( 0%) | 471 (47%) | 775 ( 42%) | 2,121 ( 74%) |  | 3,821 ( 57%) |
| Bromodichloromethane ^b^ | 257 ( 85%) | 286 ( 6%) | 439 ( 1%) | 765 ( 24%) | 2,139 ( 30%) |  | 3,886 ( 27%) |
| Chlorobenzene | 253 ( 71%) | 0 (---) | 0 (---) | 784 (100%) | 2,223 ( 99%) |  | 3,260 ( 97%) |
| cis-1,2-Dichloroethene [cis-1,2-Dichloroethelene] | 245 (100%) | 0 (---) | 0 (---) | 784 (100%) | 2,183 (100%) |  | 3,212 (100%) |
| Dibromochloromethane [Chlorodibromomethane] ^b^ | 249 ( 84%) | 283 (12%) | 440 (18%) | 772 ( 53%) | 2,093 ( 52%) |  | 3,837 ( 47%) |
| Dibromomethane | 99 (100%) | 0 (---) | 0 (---) | 777 (100%) | 2,186 (100%) |  | 3,062 (100%) |
| Dichloromethane [Methylene chloride] | 107 ( 95%) | 0 (---) | 0 (---) | 704 (100%) | 2,022 (100%) |  | 2,833 (100%) |
| Ethylbenzene | 111 ( 5%) | 212 ( 9%) | 502 (36%) | 740 ( 30%) | 2,111 ( 72%) |  | 3,676 ( 53%) |
| Furan | 0 (---) | 0 (---) | 0 (---) | 0 (---) | 2,073 ( 87%) |  | 2,073 ( 87%) |
| Hexachloroethane | 0 (---) | 0 (---) | 0 (---) | 784 (100%) | 2,214 (100%) |  | 2,998 (100%) |
| Isopropylbenzene [Cumene] | 0 (---) | 0 (---) | 0 (---) | 0 (---) | 2,193 ( 0%) |  | 2,193 ( 0%) |
| m-/p-Xylene | 276 ( 59%) | 238 ( 3%) | 550 ( 3%) | 773 ( 1%) | 2,059 ( 18%) |  | 3,896 ( 15%) |
| Methyl-tert-butyl ether [MTBE] ^b^ | 0 (---) | 229 ( 2%) | 364 ( 5%) | 759 ( 20%) | 2,004 ( 74%) |  | 3,356 ( 49%) |
| n-Hexane [Hexane] | 0 (---) | 0 (---) | 0 (---) | 0 (---) | 2,096 ( 99%) |  | 2,096 ( 99%) |
| Nitrobenzene | 0 (---) | 0 (---) | 0 (---) | 650 (100%) | 2,149 (100%) |  | 2,799 (100%) |
| Nitromethane ^b^ | 0 (---) | 0 (---) | 0 (---) | 0 (---) | 1,810 ( 0%) |  | 1,810 ( 0%) |
| o-Xylene | 111 ( 3%) | 248 (41%) | 563 (55%) | 785 ( 62%) | 2,159 ( 67%) |  | 3,866 ( 60%) |
| Styrene | 111 ( 8%) | 227 ( 4%) | 542 (43%) | 754 ( 60%) | 0 (---) |  | 1,634 ( 43%) |
| Tetrachloroethene [Perchloroethylene] | 109 ( 16%) | 229 (24%) | 558 (63%) | 755 ( 85%) | 2,101 ( 86%) |  | 3,752 ( 77%) |
| Tetrachloromethane [Carbon tetrachloride] | 248 ( 97%) | 233 (72%) | 397 (92%) | 782 ( 99%) | 2,161 (100%) |  | 3,821 ( 97%) |
| Toluene | 106 ( 0%) | 246 ( 1%) | 552 ( 5%) | 768 ( 4%) | 0 (---) |  | 1,672 ( 4%) |
| trans-1,2-Dichloroethene | 250 ( 98%) | 0 (---) | 0 (---) | 785 (100%) | 2,188 (100%) |  | 3,223 (100%) |
| Tribromomethane [Bromoform] ^b^ | 104 ( 95%) | 266 (24%) | 436 (16%) | 757 ( 60%) | 2,136 ( 79%) |  | 3,699 ( 64%) |
| Trichloroethene [Trichloroethylene] | 109 ( 80%) | 242 (87%) | 526 (96%) | 738 ( 99%) | 2,133 ( 99%) |  | 3,748 ( 97%) |
| Trichloromethane [Chloroform] ^b^ | 227 ( 45%) | 207 ( 0%) | 410 ( 3%) | 720 ( 7%) | 2,050 ( 13%) |  | 3,614 ( 12%) |
|  |  |  |  |  |  |  |  |
|  |  |  |  |  |  |  |  |
| Abbreviations: ANA = antinuclear antibodies; LOD = limit of detection; NHANES = National Health and Nutrition Examination Survey. | | | | | | | |
| ^a^ All of these volatile organic compounds were measured in whole blood, with concentration units of ng/mL, except as indicated by footnote b. | | | | | | | |
| ^b^ These volatile organic compounds were measured in whole blood, with concentration units of pg/mL. | | | | | | | |

| Supplemental Table 1d. Summary information about volatile organic compound metabolites among NHANES participants with data on ANA. | | | | | | | |
| --- | --- | --- | --- | --- | --- | --- | --- |
|  |  |  |  |  |  |  |  |
|  |  |  |  |  |  |  |  |
|  | Number of Participants with Xenobiotic and ANA Data (Percent with Concentration <LOD) | | | | | | |
|  | Cycle 1 ^a^ | Cycle 2 ^a^ | Cycle 3 ^a^ | Cycle 4 ^a^ | Cycle 5 |  | All Cycles |
| Xenobiotic Name [Alternative/Abbreviated Name] ^b^ | 1988-1991 | 1999-2000 | 2001-2002 | 2003-2004 | 2011-2012 |  | Combined |
|  |  |  |  |  |  |  |  |
| 2-Aminothiazoline-4-carboxylic acid [ATCA] | 0 (---) | 0 (---) | 0 (---) | 0 (---) | 2,013 ( 4%) |  | 2,013 ( 4%) |
| 2-Methylhippuric acid [2MHA] | 0 (---) | 0 (---) | 0 (---) | 0 (---) | 2,013 ( 4%) |  | 2,013 ( 4%) |
| 2-Thioxothiazolidine-4-carboxylic acid [TTCA] | 0 (---) | 0 (---) | 0 (---) | 0 (---) | 2,013 ( 62%) |  | 2,013 ( 62%) |
| 3- & 4-Methylhippuric acid [34MH] | 0 (---) | 0 (---) | 0 (---) | 0 (---) | 2,013 ( 0%) |  | 2,013 ( 0%) |
| Mandelic acid [MADA] | 0 (---) | 0 (---) | 0 (---) | 0 (---) | 2,013 ( 1%) |  | 2,013 ( 1%) |
| N-Acetyl-S-(1-hydroxymethyl-2-propenyl)-L-cysteine [MHB1] | 0 (---) | 0 (---) | 0 (---) | 0 (---) | 2,013 ( 98%) |  | 2,013 ( 98%) |
| N-Acetyl-S-(1,2-dichlorovinyl)-L-cysteine [1DCV] | 0 (---) | 0 (---) | 0 (---) | 0 (---) | 2,013 (100%) |  | 2,013 (100%) |
| N-Acetyl-S-(2-carbamoyl-2-hydroxyethyl)-L-cysteine [GAMA] | 0 (---) | 0 (---) | 0 (---) | 0 (---) | 2,013 ( 50%) |  | 2,013 ( 50%) |
| N-Acetyl-S-(2-carbamoylethyl)-L-cysteine [AAMA] | 0 (---) | 0 (---) | 0 (---) | 0 (---) | 2,013 ( 0%) |  | 2,013 ( 0%) |
| N-Acetyl-S-(2-carboxyethyl)-L-cysteine [CEMA] | 0 (---) | 0 (---) | 0 (---) | 0 (---) | 2,013 ( 1%) |  | 2,013 ( 1%) |
| N-Acetyl-S-(2-cyanoethyl)-L-cysteine [CYMA] | 0 (---) | 0 (---) | 0 (---) | 0 (---) | 2,013 ( 8%) |  | 2,013 ( 8%) |
| N-Acetyl-S-(2-hydroxy-3-butenyl)-L-cysteine [MHB2] | 0 (---) | 0 (---) | 0 (---) | 0 (---) | 2,013 ( 79%) |  | 2,013 ( 79%) |
| N-Acetyl-S-(2-hydroxyethyl)-L-cysteine [HEMA] | 0 (---) | 0 (---) | 0 (---) | 0 (---) | 2,013 ( 38%) |  | 2,013 ( 38%) |
| N-Acetyl-S-(2-hydroxypropyl)-L-cysteine [HPM2] | 0 (---) | 0 (---) | 0 (---) | 0 (---) | 2,013 ( 4%) |  | 2,013 ( 4%) |
| N-Acetyl-S-(2,2-dichlorovinyl)-L-cysteine [2DCV] | 0 (---) | 0 (---) | 0 (---) | 0 (---) | 2,013 (100%) |  | 2,013 (100%) |
| N-Acetyl-S-(3-hydroxypropyl-1-methyl)-L-cysteine [HPMM] | 0 (---) | 0 (---) | 0 (---) | 0 (---) | 2,013 ( 0%) |  | 2,013 ( 0%) |
| N-Acetyl-S-(3-hydroxypropyl)-L-cysteine [HPMA] | 0 (---) | 0 (---) | 0 (---) | 0 (---) | 2,013 ( 0%) |  | 2,013 ( 0%) |
| N-Acetyl-S-(3,4-dihydroxybutyl)-L-cysteine [DHBM] | 0 (---) | 0 (---) | 0 (---) | 0 (---) | 2,013 ( 0%) |  | 2,013 ( 0%) |
| N-Acetyl-S-(4-hydroxy-2-butenyl)-L-cysteine [MHB3] | 0 (---) | 0 (---) | 0 (---) | 0 (---) | 2,013 ( 2%) |  | 2,013 ( 2%) |
| N-Acetyl-S-(benzyl)-L-cysteine [BMA] | 0 (---) | 0 (---) | 0 (---) | 0 (---) | 2,013 ( 1%) |  | 2,013 ( 1%) |
| N-Acetyl-S-(dimethylphenyl)-L-cysteine [DPMA] | 0 (---) | 0 (---) | 0 (---) | 0 (---) | 2,013 (100%) |  | 2,013 (100%) |
| N-Acetyl-S-(n-methylcarbamoyl)-L-cysteine [AMCA] | 0 (---) | 0 (---) | 0 (---) | 0 (---) | 2,013 ( 0%) |  | 2,013 ( 0%) |
| N-Acetyl-S-(n-propyl)-L-cysteine [BPMA] | 0 (---) | 0 (---) | 0 (---) | 0 (---) | 1,960 ( 23%) |  | 1,960 ( 23%) |
| N-Acetyl-S-(phenyl-2-hydroxyethyl)-L-cysteine [PHEM] | 0 (---) | 0 (---) | 0 (---) | 0 (---) | 2,013 ( 60%) |  | 2,013 ( 60%) |
| N-Acetyl-S-(phenyl)-L-cysteine [PMA] | 0 (---) | 0 (---) | 0 (---) | 0 (---) | 2,013 ( 52%) |  | 2,013 ( 52%) |
| N-Acetyl-S-(trichlorovinyl)-L-cysteine [TCVM] | 0 (---) | 0 (---) | 0 (---) | 0 (---) | 2,013 (100%) |  | 2,013 (100%) |
| Phenylglyoxylic acid [PHGA] | 0 (---) | 0 (---) | 0 (---) | 0 (---) | 2,013 ( 1%) |  | 2,013 ( 1%) |
|  |  |  |  |  |  |  |  |
|  |  |  |  |  |  |  |  |
| Abbreviations: ANA = antinuclear antibodies; LOD = limit of detection; NHANES = National Health and Nutrition Examination Survey. | | | | | | | |
| ^a^ None of these metabolites were measured in 1988-1991, 1999-2000, 2001-2002, or 2003-2004, but Cycles 1-4 are included in the table for completeness. | | | | | | | |
| ^b^ All of these volatile organic compound metabolites were measured in urine, with concentration units of μg/L (or equivalently ng/mL). | | | | | | | |

| Supplemental Table 1e. Summary information about metals and metalloids among NHANES participants with data on ANA. | | | | | | | | | |
| --- | --- | --- | --- | --- | --- | --- | --- | --- | --- |
|  |  |  |  |  |  |  |  |  |  |
|  |  |  |  |  |  |  |  |  |  |
|  |  |  | Number of Participants with Xenobiotic and ANA Data (Percent with Concentration <LOD) | | | | | | |
|  |  |  | Cycle 1 | Cycle 2 | Cycle 3 | Cycle 4 | Cycle 5 |  | All Cycles |
| Xenobiotic Name ^a^ | Matrix ^b^ |  | 1988-1991 | 1999-2000 | 2001-2002 | 2003-2004 | 2011-2012 |  | Combined |
|  |  |  |  |  |  |  |  |  |  |
| Antimony | urine |  | 0 (---) | 0 (---) | 0 (---) | 0 (---) | 2,040 (40%) |  | 2,040 (40%) |
| Arsenic, total | urine |  | 0 (---) | 0 (---) | 0 (---) | 0 (---) | 2,040 ( 4%) |  | 2,040 ( 4%) |
| Arsenic (V) acid ^c^ | urine |  | 0 (---) | 0 (---) | 0 (---) | 0 (---) | 2,044 (97%) |  | 2,044 (97%) |
| Arsenobetaine ^c^ | urine |  | 0 (---) | 0 (---) | 0 (---) | 0 (---) | 2,044 (54%) |  | 2,044 (54%) |
| Arsenocholine ^c^ | urine |  | 0 (---) | 0 (---) | 0 (---) | 0 (---) | 2,044 (96%) |  | 2,044 (96%) |
| Arsenous (III) acid ^c^ | urine |  | 0 (---) | 0 (---) | 0 (---) | 0 (---) | 2,044 (70%) |  | 2,044 (70%) |
| Dimethylarsinic acid ^c^ | urine |  | 0 (---) | 0 (---) | 0 (---) | 0 (---) | 2,044 (21%) |  | 2,044 (21%) |
| Monomethylarsonic acid ^c^ | urine |  | 0 (---) | 0 (---) | 0 (---) | 0 (---) | 2,044 (73%) |  | 2,044 (73%) |
| Trimethylarsine oxide ^c^ | urine |  | 0 (---) | 0 (---) | 0 (---) | 0 (---) | 2,044 (98%) |  | 2,044 (98%) |
| Barium | urine |  | 0 (---) | 0 (---) | 0 (---) | 0 (---) | 2,040 ( 1%) |  | 2,040 ( 1%) |
| Cadmium | blood |  | 0 (---) | 1,577 (23%) | 1,191 (26%) | 1,755 (23%) | 4,261 (19%) |  | 8,784 (22%) |
| Cadmium | urine |  | 4,523 (10%) | 0 (---) | 0 (---) | 0 (---) | 2,040 (14%) |  | 6,563 (12%) |
| Cesium | urine |  | 0 (---) | 0 (---) | 0 (---) | 0 (---) | 2,040 ( 0%) |  | 2,040 ( 0%) |
| Cobalt | urine |  | 0 (---) | 0 (---) | 0 (---) | 0 (---) | 2,040 ( 1%) |  | 2,040 ( 1%) |
| Copper ^d^ | serum |  | 0 (---) | 0 (---) | 0 (---) | 0 (---) | 1,728 ( 0%) |  | 1,728 ( 0%) |
| Lead ^d^ | blood |  | 4,708 ( 7%) | 1,577 ( 1%) | 1,191 ( 1%) | 1,755 ( 0%) | 4,261 ( 1%) |  | 13,492 ( 3%) |
| Lead | urine |  | 0 (---) | 0 (---) | 0 (---) | 0 (---) | 2,040 ( 4%) |  | 2,040 ( 4%) |
| Manganese | blood |  | 0 (---) | 0 (---) | 0 (---) | 0 (---) | 4,261 ( 0%) |  | 4,261 ( 0%) |
| Manganese | urine |  | 0 (---) | 0 (---) | 0 (---) | 0 (---) | 2,040 (32%) |  | 2,040 (32%) |
| Mercury, total | blood |  | 0 (---) | 0 (---) | 0 (---) | 1,755 ( 8%) | 4,261 ( 4%) |  | 6,016 ( 5%) |
| Mercury, ethyl | blood |  | 0 (---) | 0 (---) | 0 (---) | 0 (---) | 4,258 (96%) |  | 4,258 (96%) |
| Mercury, inorganic | blood |  | 0 (---) | 0 (---) | 0 (---) | 1,724 (74%) | 4,258 (74%) |  | 5,982 (74%) |
| Mercury, methyl | blood |  | 0 (---) | 0 (---) | 0 (---) | 0 (---) | 4,258 (12%) |  | 4,258 (12%) |
| Mercury, urinary | urine |  | 0 (---) | 0 (---) | 0 (---) | 0 (---) | 1,711 ( 4%) |  | 1,711 ( 4%) |
| Molybdenum | urine |  | 0 (---) | 0 (---) | 0 (---) | 0 (---) | 2,040 ( 0%) |  | 2,040 ( 0%) |
| Selenium | blood |  | 0 (---) | 0 (---) | 0 (---) | 0 (---) | 4,261 ( 0%) |  | 4,261 ( 0%) |
| Selenium | serum |  | 4,541 ( 0%) | 0 (---) | 0 (---) | 0 (---) | 1,728 ( 0%) |  | 6,269 ( 0%) |
| Strontium | urine |  | 0 (---) | 0 (---) | 0 (---) | 0 (---) | 2,038 ( 0%) |  | 2,038 ( 0%) |
| Thallium | urine |  | 0 (---) | 0 (---) | 0 (---) | 0 (---) | 2,040 ( 1%) |  | 2,040 ( 1%) |
| Tin | urine |  | 0 (---) | 0 (---) | 0 (---) | 0 (---) | 2,040 (16%) |  | 2,040 (16%) |
| Tungsten | urine |  | 0 (---) | 0 (---) | 0 (---) | 0 (---) | 2,027 (16%) |  | 2,027 (16%) |
| Uranium | urine |  | 0 (---) | 0 (---) | 0 (---) | 0 (---) | 2,040 (26%) |  | 2,040 (26%) |
| Zinc ^d^ | serum |  | 0 (---) | 0 (---) | 0 (---) | 0 (---) | 1,728 ( 0%) |  | 1,728 ( 0%) |
|  |  |  |  |  |  |  |  |  |  |
|  |  |  |  |  |  |  |  |  |  |
| Abbreviations: ANA = antinuclear antibodies; LOD = limit of detection; NHANES = National Health and Nutrition Examination Survey. | | | | | | | | | |
| ^a^ All of these metals and metalloids were measured with concentration units of μg/L (or equivalently ng/mL), except as indicated by footnotes c and d. | | | | | | | | | |
| ^b^ Metals and metalloids were measured in various matrices (serum, urine, and whole blood) and some of them (cadmium, lead, manganese, mercury, and selenium) are listed twice because they were measured in two different matrices. | | | | | | | | | |
| ^c^ These arsenic (As) compounds were measured with concentration units of μg As/L. | | | | | | | | | |
| ^d^ These metals and metalloids were measured with concentration units of μg/dL. | | | | | | | | | |

| Supplemental Table 1f. Summary information about metabolites of phthalates, phthalate alternatives, and polycyclic aromatic hydrocarbons among NHANES participants with data on ANA. | | | | | | | | | |
| --- | --- | --- | --- | --- | --- | --- | --- | --- | --- |
|  |  |  |  |  |  |  |  |  |  |
|  |  |  |  |  |  |  |  |  |  |
|  |  |  | Number of Participants with Xenobiotic and ANA Data (Percent with Concentration <LOD) | | | | | | |
| Xenobiotic Class | |  | Cycle 1 | Cycle 2 ^a^ | Cycle 3 ^a^ | Cycle 4 ^a^ | Cycle 5 |  | All Cycles |
|  | Xenobiotic Name [Alternative/Abbreviated Name] |  | 1988-1991 | 1999-2000 | 2001-2002 | 2003-2004 | 2011-2012 |  | Combined |
|  |  |  |  |  |  |  |  |  |  |
| Phthalate and Phthalate Alternative Metabolites ^b^ | |  |  |  |  |  |  |  |  |
|  | Cyclohexane 1,2-dicarboxylic acid monohydroxy isononyl ester [MHNCH] |  | 0 (---) | 0 (---) | 0 (---) | 0 (---) | 1,704 ( 0%) |  | 1,704 ( 0%) |
|  | Mono-(2-ethyl-5-hydroxyhexyl) phthalate [MEHHP] |  | 0 (---) | 0 (---) | 0 (---) | 0 (---) | 1,704 ( 0%) |  | 1,704 ( 0%) |
|  | Mono-(2-ethyl-5-oxohexyl) phthalate [MEOHP] |  | 0 (---) | 0 (---) | 0 (---) | 0 (---) | 1,704 ( 1%) |  | 1,704 ( 1%) |
|  | Mono-(2-ethyl)-hexyl phthalate [MEHP] |  | 0 (---) | 0 (---) | 0 (---) | 0 (---) | 1,704 (24%) |  | 1,704 (24%) |
|  | Mono-(3-carboxypropyl) phthalate [MCPP] |  | 0 (---) | 0 (---) | 0 (---) | 0 (---) | 1,704 ( 2%) |  | 1,704 ( 2%) |
|  | Mono-2-ethyl-5-carboxypentyl phthalate [MECPP] |  | 0 (---) | 0 (---) | 0 (---) | 0 (---) | 1,704 ( 0%) |  | 1,704 ( 0%) |
|  | Mono-benzyl phthalate [MBzP] |  | 0 (---) | 0 (---) | 0 (---) | 0 (---) | 1,704 ( 2%) |  | 1,704 ( 2%) |
|  | Mono-ethyl phthalate [MEP] |  | 0 (---) | 0 (---) | 0 (---) | 0 (---) | 1,704 ( 0%) |  | 1,704 ( 0%) |
|  | Mono-isobutyl phthalate [MiBP] |  | 0 (---) | 0 (---) | 0 (---) | 0 (---) | 1,704 ( 1%) |  | 1,704 ( 1%) |
|  | Mono-isononyl phthalate [MiNP] |  | 0 (---) | 0 (---) | 0 (---) | 0 (---) | 1,704 (42%) |  | 1,704 (42%) |
|  | Mono-n-butyl phthalate [MnBP] |  | 0 (---) | 0 (---) | 0 (---) | 0 (---) | 1,704 ( 6%) |  | 1,704 ( 6%) |
|  | Mono-n-methyl phthalate [MMP] |  | 0 (---) | 0 (---) | 0 (---) | 0 (---) | 1,704 (39%) |  | 1,704 (39%) |
|  | Mono-(carboxynonyl) phthalate [MCNP] |  | 0 (---) | 0 (---) | 0 (---) | 0 (---) | 1,704 ( 1%) |  | 1,704 ( 1%) |
|  | Mono-(carboxyoctyl) phthalate [MCOP] |  | 0 (---) | 0 (---) | 0 (---) | 0 (---) | 1,704 ( 0%) |  | 1,704 ( 0%) |
|  |  |  |  |  |  |  |  |  |  |
| Polycyclic Aromatic Hydrocarbon Metabolites ^c^ | |  |  |  |  |  |  |  |  |
|  | 1-Hydroxynaphthalene [1-Naphthol] |  | 265 (13%) | 0 (---) | 0 (---) | 0 (---) | 2,032 ( 0%) |  | 2,297 ( 2%) |
|  | 1-Hydroxyphenanthrene |  | 0 (---) | 0 (---) | 0 (---) | 0 (---) | 2,032 ( 0%) |  | 2,032 ( 0%) |
|  | 1-Hydroxypyrene |  | 0 (---) | 0 (---) | 0 (---) | 0 (---) | 2,027 ( 1%) |  | 2,027 ( 1%) |
|  | 2-Hydroxyfluorene |  | 0 (---) | 0 (---) | 0 (---) | 0 (---) | 2,032 ( 0%) |  | 2,032 ( 0%) |
|  | 2-Hydroxynaphthalene [2-Naphthol] |  | 262 (15%) | 0 (---) | 0 (---) | 0 (---) | 2,032 ( 0%) |  | 2,294 ( 2%) |
|  | 2-Hydroxyphenanthrene |  | 0 (---) | 0 (---) | 0 (---) | 0 (---) | 2,028 ( 2%) |  | 2,028 ( 2%) |
|  | 3-Hydroxyfluorene |  | 0 (---) | 0 (---) | 0 (---) | 0 (---) | 2,028 ( 2%) |  | 2,028 ( 2%) |
|  | 3-Hydroxyphenanthrene |  | 0 (---) | 0 (---) | 0 (---) | 0 (---) | 2,032 ( 3%) |  | 2,032 ( 3%) |
|  | 4-Hydroxyphenanthrene |  | 0 (---) | 0 (---) | 0 (---) | 0 (---) | 2,028 (20%) |  | 2,028 (20%) |
|  | 9-Hydroxyfluorene |  | 0 (---) | 0 (---) | 0 (---) | 0 (---) | 2,031 ( 0%) |  | 2,031 ( 0%) |
|  |  |  |  |  |  |  |  |  |  |
|  |  |  |  |  |  |  |  |  |  |
| Abbreviations: ANA = antinuclear antibodies; LOD = limit of detection; NHANES = National Health and Nutrition Examination Survey. | | | | | | | | | |
| ^a^ None of these xenobiotics were measured in 1999-2000, 2001-2002, or 2003-2004, but Cycles 2-4 are included in the table for completeness. | | | | | | | | | |
| ^b^ All of these phthalate and phthalate alternative metabolites were measured in urine, with concentration units of μg/L (or equivalently ng/mL). | | | | | | | | | |
| ^c^ All of these polycyclic aromatic hydrocarbon metabolites were measured in urine, with concentration units of ng/L. | | | | | | | | | |

| Supplemental Table 1g. Summary information about pesticides and their metabolites among NHANES participants with data on ANA. | | | | | | | | | | |
| --- | --- | --- | --- | --- | --- | --- | --- | --- | --- | --- |
|  |  |  |  |  |  |  |  |  |  |  |
|  |  |  |  |  |  |  |  |  |  |  |
|  |  |  |  | Number of Participants with Xenobiotic and ANA Data (Percent with Concentration <LOD) | | | | | | |
| Xenobiotic Class | |  |  | Cycle 1 | Cycle 2 | Cycle 3 | Cycle 4 | Cycle 5 ^a^ |  | All Cycles |
|  | Xenobiotic Name [Alternative/Abbreviated Name] | Matrix |  | 1988-1991 | 1999-2000 | 2001-2002 | 2003-2004 | 2011-2012 |  | Combined |
|  |  |  |  |  |  |  |  |  |  |  |
| Organochlorine Pesticides and Metabolites ^b^ | |  |  |  |  |  |  |  |  |  |
|  | 2,4,5-Trichlorophenol [2,4,5-TCP] ^c^ | urine |  | 258 (73%) | 0 (---) | 0 (---) | 1,721 (63%) | 0 (---) |  | 1,979 ( 65%) |
|  | 2,4,6-Trichlorophenol ^c^ | urine |  | 244 (84%) | 0 (---) | 0 (---) | 1,721 (68%) | 0 (---) |  | 1,965 ( 70%) |
|  | Aldrin | serum |  | 0 (---) | 0 (---) | 1,166 (100%) | 0 (---) | 0 (---) |  | 1,166 (100%) |
|  | beta-Hexachlorocyclohexane | serum |  | 0 (---) | 1,511 (36%) | 1,177 ( 27%) | 0 (---) | 0 (---) |  | 2,688 ( 32%) |
|  | Dieldrin | serum |  | 0 (---) | 0 (---) | 1,114 ( 34%) | 0 (---) | 0 (---) |  | 1,114 ( 34%) |
|  | Endrin | serum |  | 0 (---) | 0 (---) | 1,122 (100%) | 0 (---) | 0 (---) |  | 1,122 (100%) |
|  | gamma-Hexachlorocyclohexane | serum |  | 0 (---) | 1,437 (97%) | 1,169 ( 99%) | 0 (---) | 0 (---) |  | 2,606 ( 98%) |
|  | Heptachlor epoxide | serum |  | 0 (---) | 1,249 (67%) | 1,164 ( 40%) | 0 (---) | 0 (---) |  | 2,413 ( 54%) |
|  | Hexachlorobenzene | serum |  | 0 (---) | 1,356 (98%) | 1,174 ( 91%) | 0 (---) | 0 (---) |  | 2,530 ( 95%) |
|  | Mirex | serum |  | 0 (---) | 1,464 (92%) | 1,178 ( 66%) | 0 (---) | 0 (---) |  | 2,642 ( 81%) |
|  | Oxychlordane | serum |  | 0 (---) | 1,308 (46%) | 1,150 ( 18%) | 0 (---) | 0 (---) |  | 2,458 ( 33%) |
|  | o,p'-Dichlorodiphenyltrichloroethane [o,p'-DDT] | serum |  | 0 (---) | 1,312 (99%) | 1,175 ( 98%) | 0 (---) | 0 (---) |  | 2,487 ( 98%) |
|  | p,p'-Dichlorodiphenyltrichloroethane [p,p'-DDT] | serum |  | 0 (---) | 1,321 (71%) | 1,192 ( 61%) | 0 (---) | 0 (---) |  | 2,513 ( 66%) |
|  | p,p'-Dichlorodiphenyltrichloroethylene [p,p'-DDE] | serum |  | 0 (---) | 1,560 ( 0%) | 1,190 ( 0%) | 0 (---) | 0 (---) |  | 2,750 ( 0%) |
|  | trans-Nonachlor | serum |  | 0 (---) | 1,538 (30%) | 1,173 ( 10%) | 0 (---) | 0 (---) |  | 2,711 ( 21%) |
|  |  |  |  |  |  |  |  |  |  |  |
| Carbamate Pesticide Metabolites ^d^ | |  |  |  |  |  |  |  |  |  |
|  | 2-Isopropoxyphenol | urine |  | 268 (86%) | 1,045 (97%) | 1,153 (100%) | 1,624 (100%) | 0 (---) |  | 4,090 (98%) |
|  | Carbofuranphenol | urine |  | 265 (98%) | 1,085 (87%) | 1,162 ( 99%) | 1,627 (100%) | 0 (---) |  | 4,139 (96%) |
|  |  |  |  |  |  |  |  |  |  |  |
|  |  |  |  |  |  |  |  |  |  |  |
| Abbreviations: ANA = antinuclear antibodies; LOD = limit of detection; NHANES = National Health and Nutrition Examination Survey. | | | | | | | | | | |
| ^a^ None of these xenobiotics were measured in 2011-2012, but Cycle 5 is included in the table for completeness. | | | | | | | | | | |
| ^b^ All of these organochlorine pesticides and metabolites were measured in serum and were lipid adjusted, with concentration units of ng/g lipid, except as indicated by footnote c. | | | | | | | | | | |
| ^c^ These organochlorine pesticides and metabolites were measured in urine, with concentration units of μg/L (or equivalently ng/mL). | | | | | | | | | | |
| ^d^ Both carbamate pesticide metabolites were measured in urine, with concentration units of μg/L (or equivalently ng/mL). | | | | | | | | | | |

| Supplemental Table 1h. Summary information about herbicides, fungicides, insecticides, and their metabolites among NHANES participants with data on ANA. | | | | | | | | | |
| --- | --- | --- | --- | --- | --- | --- | --- | --- | --- |
|  |  |  |  |  |  |  |  |  |  |
|  |  |  |  |  |  |  |  |  |  |
|  |  |  | Number of Participants with Xenobiotic and ANA Data (Percent with Concentration <LOD) | | | | | | |
| Xenobiotic Class | |  | Cycle 1 | Cycle 2 | Cycle 3 | Cycle 4 | Cycle 5 |  | All Cycles |
|  | Xenobiotic Name [Alternative/Abbreviated Name] ^a^ |  | 1988-1991 | 1999-2000 | 2001-2002 | 2003-2004 | 2011-2012 |  | Combined |
|  |  |  |  |  |  |  |  |  |  |
| Herbicides and Metabolites | |  |  |  |  |  |  |  |  |
|  | 2,4-Dichlorophenoxyacetic acid [2,4-D] |  | 265 (80%) | 1,076 (47%) | 1,123 ( 75%) | 0 (---) | 1,199 (27%) |  | 3,663 (51%) |
|  | 2,4,5-Trichlorophenoxyacetic acid [2,4,5-T] |  | 0 (---) | 994 (97%) | 1,168 (100%) | 1,642 (90%) | 0 (---) |  | 3,804 (95%) |
|  | Acetochlor mercapturate |  | 0 (---) | 0 (---) | 1,152 ( 98%) | 0 (---) | 0 (---) |  | 1,152 (98%) |
|  | Alachlor mercapturate |  | 0 (---) | 1,061 (66%) | 0 (---) | 0 (---) | 0 (---) |  | 1,061 (66%) |
|  | Atrazine mercapturate |  | 0 (---) | 1,031 (95%) | 1,142 ( 99%) | 0 (---) | 0 (---) |  | 2,173 (97%) |
|  | Metolachlor mercapturate |  | 0 (---) | 0 (---) | 1,168 ( 97%) | 0 (---) | 0 (---) |  | 1,168 (97%) |
|  |  |  |  |  |  |  |  |  |  |
| Fungicides and Metabolites | |  |  |  |  |  |  |  |  |
|  | Pentachlorophenol |  | 246 (22%) | 0 (---) | 0 (---) | 1,603 (64%) | 0 (---) |  | 1,849 (59%) |
|  | ortho-Phenylphenol |  | 0 (---) | 0 (---) | 0 (---) | 1,721 (45%) | 0 (---) |  | 1,721 (45%) |
|  |  |  |  |  |  |  |  |  |  |
| Pyrethroid Insecticide Metabolites | |  |  |  |  |  |  |  |  |
|  | 3-Phenoxybenzoic acid [3PBA] |  | 0 (---) | 1,088 (30%) | 1,169 ( 26%) | 0 (---) | 1,219 ( 8%) |  | 3,476 (21%) |
|  | 4-Fluoro-3-phenoxybenzoic acid [4F3PBA] |  | 0 (---) | 1,059 (97%) | 1,169 (100%) | 0 (---) | 1,225 (83%) |  | 3,453 (93%) |
|  | Cis-3-(2,2-dibromovinyl)-2,2-dimethylcyclopropane carboxylic acid [cis-DBCA] |  | 0 (---) | 926 (99%) | 1,169 ( 99%) | 0 (---) | 0 (---) |  | 2,095 (99%) |
|  | Cis-3-(2,2-dichlorovinyl)-2,2-dimethylcyclopropane carboxylic acid [cis-DCCA] |  | 0 (---) | 1,066 (56%) | 1,169 ( 66%) | 0 (---) | 0 (---) |  | 2,235 (61%) |
|  | trans-3-(2,2-Dichlorovinyl)-2,2-dimethylcyclopropane carboxylic acid [trans-DCCA] |  | 0 (---) | 1,077 (67%) | 1,164 ( 74%) | 0 (---) | 1,163 (91%) |  | 3,404 (78%) |
|  |  |  |  |  |  |  |  |  |  |
| Organophosphorus Insecticides: Dialkyl Phosphate Metabolites | | |  |  |  |  |  |  |  |
|  | Diethyldithiophosphate [DEDTP] |  | 45 (64%) | 1,063 (47%) | 1,155 (79%) | 1,702 (90%) | 1,225 (95%) |  | 5,190 (80%) |
|  | Diethylphosphate [DEP] |  | 39 ( 0%) | 1,063 (29%) | 1,159 (49%) | 1,669 (48%) | 1,219 ( 2%) |  | 5,149 (33%) |
|  | Diethylthiophosphate [DETP] |  | 44 ( 0%) | 1,063 (49%) | 1,158 (25%) | 1,683 (48%) | 1,211 (25%) |  | 5,159 (38%) |
|  | Dimethyldithiophosphate [DMDTP] |  | 41 ( 0%) | 1,063 (50%) | 1,158 (63%) | 1,676 (57%) | 1,226 (45%) |  | 5,164 (54%) |
|  | Dimethylphosphate [DMP] |  | 42 ( 0%) | 1,063 (47%) | 1,159 (51%) | 1,702 (49%) | 1,208 ( 2%) |  | 5,174 (38%) |
|  | Dimethylthiophosphate [DMTP] |  | 43 (26%) | 1,062 (39%) | 1,158 (47%) | 1,702 (20%) | 1,219 ( 5%) |  | 5,184 (26%) |
|  |  |  |  |  |  |  |  |  |  |
| Organophosphorus Insecticides: Specific Pesticides & Metabolites | | |  |  |  |  |  |  |  |
|  | 2-(Diethylamino)-6-methylpyrimidin-4-ol/one |  | 0 (---) | 0 (--) | 1,144 (94%) | 0 (---) | 0 (--) |  | 1,144 (94%) |
|  | 2-Isopropyl-4-methyl-6-hydroxypyrimidine [Oxypyrimidine] |  | 0 (---) | 994 (69%) | 1,168 (96%) | 0 (---) | 1,222 (74%) |  | 3,384 (80%) |
|  | 3-Chloro-7-hydroxy-4-methyl-2H-chromen-2-one/ol |  | 0 (---) | 0 (--) | 1,140 (97%) | 0 (---) | 0 (---) |  | 1,140 (97%) |
|  | 3,5,6-Trichloro-2-pyridinol [TCPy] |  | 269 (16%) | 1,086 ( 8%) | 1,151 (28%) | 0 (---) | 0 (---) |  | 2,506 (18%) |
|  | Malathion dicarboxylic acid |  | 0 (---) | 1,053 (47%) | 0 (---) | 0 (---) | 0 (---) |  | 1,053 (47%) |
|  | para-Nitrophenol |  | 257 (51%) | 1,085 (77%) | 1,138 (52%) | 0 (---) | 1,189 ( 4%) |  | 3,669 (44%) |
|  |  |  |  |  |  |  |  |  |  |
| Insect Repellents and Metabolites | |  |  |  |  |  |  |  |  |
|  | 3-(Diethylcarbamoyl)benzoic acid [DCBA or DEET acid] |  | 0 (---) | 0 (---) | 0 (---) | 0 (---) | 1,226 (18%) |  | 1,226 (18%) |
|  | N,N-Diethyl-3-(hydroxymethyl)benzamide [DHMB] |  | 0 (---) | 0 (---) | 0 (---) | 0 (---) | 1,220 (92%) |  | 1,220 (92%) |
|  | N,N-Diethyl-meta-toluamide [DEET] |  | 0 (---) | 1,073 (84%) | 1,168 (88%) | 0 (---) | 1,225 (96%) |  | 3,466 (90%) |
|  |  |  |  |  |  |  |  |  |  |
|  |  |  |  |  |  |  |  |  |  |
| Abbreviations: ANA = antinuclear antibodies; LOD = limit of detection; NHANES = National Health and Nutrition Examination Survey. | | | | | | | | | |
| ^a^ All of these xenobiotics were measured in urine, with concentration units of μg/L (or equivalently ng/mL). | | | | | | | | | |

| Supplemental Table 1i. Summary information about perfluoroalkyl and polyfluoroalkyl substances, perchlorate and other anions, personal care and consumer product chemicals and metabolites, and tobacco biomarkers among NHANES participants with data on ANA. | | | | | | | | | | |
| --- | --- | --- | --- | --- | --- | --- | --- | --- | --- | --- |
|  |  |  |  |  |  |  |  |  |  |  |
|  |  |  |  |  |  |  |  |  |  |  |
|  |  |  |  | Number of Participants with Xenobiotic and ANA Data (Percent with Concentration <LOD) | | | | | | |
| Xenobiotic Class | |  |  | Cycle 1 | Cycle 2 | Cycle 3 | Cycle 4 | Cycle 5 |  | All Cycles |
|  | Xenobiotic Name [Alternative/Abbreviated Name] | Matrix |  | 1988-1991 | 1999-2000 | 2001-2002 | 2003-2004 | 2011-2012 |  | Combined |
|  |  |  |  |  |  |  |  |  |  |  |
| Perfluoroalkyl and Polyfluoroalkyl Substances [PFAS] ^a^ | |  |  |  |  |  |  |  |  |  |
|  | 2-(N-ethyl-perfluorooctane sulfonamido) acetic acid [EtFOSAA] | serum |  | 0 (---) | 0 (---) | 0 (---) | 0 (---) | 1,679 (95%) |  | 1,679 (95%) |
|  | 2-(N-methyl-perfluorooctane sulfonamido) acetic acid [MeFOSAA] | serum |  | 0 (---) | 0 (---) | 0 (---) | 0 (---) | 1,679 (46%) |  | 1,679 (46%) |
|  | Perfluorodecanoic acid [PFDA] | serum |  | 0 (---) | 0 (---) | 0 (---) | 0 (---) | 1,679 (15%) |  | 1,679 (15%) |
|  | Perfluorobutane sulfonic acid [PFBS] | serum |  | 0 (---) | 0 (---) | 0 (---) | 0 (---) | 1,679 (99%) |  | 1,679 (99%) |
|  | Perfluoroheptanoic acid PFHpA] | serum |  | 0 (---) | 0 (---) | 0 (---) | 0 (---) | 1,679 (80%) |  | 1,679 (80%) |
|  | Perfluorohexane sulfonic acid [PFHxS] | serum |  | 0 (---) | 0 (---) | 0 (---) | 0 (---) | 1,679 ( 2%) |  | 1,679 ( 2%) |
|  | Perfluorononanoic acid [PFNA] | serum |  | 0 (---) | 0 (---) | 0 (---) | 0 (---) | 1,679 ( 1%) |  | 1,679 ( 1%) |
|  | Perfluorooctane sulfonamide [PFOSA] | serum |  | 0 (---) | 0 (---) | 0 (---) | 0 (---) | 1,679 (99%) |  | 1,679 (99%) |
|  | Perfluorooctane sulfonic acid [PFOS] | serum |  | 0 (---) | 0 (---) | 0 (---) | 0 (---) | 1,679 ( 0%) |  | 1,679 ( 0%) |
|  | Perfluorooctanoic acid [PFOA] | serum |  | 0 (---) | 0 (---) | 0 (---) | 0 (---) | 1,679 ( 1%) |  | 1,679 ( 1%) |
|  | Perfluoroundecanoic acid [PFUnDA] | serum |  | 0 (---) | 0 (---) | 0 (---) | 0 (---) | 1,679 (38%) |  | 1,679 (38%) |
|  | Perflurododecanoic acid [PFDoH] | serum |  | 0 (---) | 0 (---) | 0 (---) | 0 (---) | 1,679 (90%) |  | 1,679 (90%) |
|  |  |  |  |  |  |  |  |  |  |  |
| Perchlorate and Other Anions ^b^ | |  |  |  |  |  |  |  |  |  |
|  | Nitrate | urine |  | 0 (---) | 0 (---) | 1,155 (0%) | 0 (---) | 2,016 (0%) |  | 3,171 (0%) |
|  | Perchlorate | urine |  | 0 (---) | 0 (---) | 1,155 (0%) | 0 (---) | 2,016 (0%) |  | 3,171 (0%) |
|  | Thiocyanate | urine |  | 0 (---) | 0 (---) | 1,155 (0%) | 0 (---) | 2,016 (0%) |  | 3,171 (0%) |
|  |  |  |  |  |  |  |  |  |  |  |
| Personal Care and Consumer Product Chemicals and Metabolites ^c^ | | |  |  |  |  |  |  |  |  |
|  | 2,4-Dichlorophenol | urine |  | 264 (34%) | 0 (---) | 0 (---) | 1,721 (16%) | 1,704 (14%) |  | 3,689 (17%) |
|  | 2,5-Dichlorophenol | urine |  | 264 ( 2%) | 0 (---) | 0 (---) | 1,721 ( 1%) | 1,704 ( 3%) |  | 3,689 ( 2%) |
|  | Benzophenone-3 | urine |  | 0 (---) | 0 (---) | 0 (---) | 1,721 ( 3%) | 1,704 ( 2%) |  | 3,425 ( 2%) |
|  | Bisphenol A | urine |  | 0 (---) | 0 (---) | 0 (---) | 1,721 ( 7%) | 1,704 (11%) |  | 3,425 ( 9%) |
|  | Butyl paraben | urine |  | 0 (---) | 0 (---) | 0 (---) | 0 (--) | 1,704 (73%) |  | 1,704 (73%) |
|  | Ethyl paraben | urine |  | 0 (---) | 0 (---) | 0 (---) | 0 (--) | 1,704 (54%) |  | 1,704 (54%) |
|  | Methyl paraben | urine |  | 0 (---) | 0 (---) | 0 (---) | 0 (--) | 1,704 ( 1%) |  | 1,704 ( 1%) |
|  | Propyl paraben [n-Propyl paraben] | urine |  | 0 (---) | 0 (---) | 0 (---) | 0 (--) | 1,704 ( 6%) |  | 1,704 ( 6%) |
|  | Triclosan | urine |  | 0 (---) | 0 (---) | 0 (---) | 1,721 (25%) | 1,704 (28%) |  | 3,425 (26%) |
|  |  |  |  |  |  |  |  |  |  |  |
| Tobacco Alkaloids and Metabolites ^d^ | |  |  |  |  |  |  |  |  |  |
|  | Cotinine | serum |  | 4,525 (9%) | 1,560 (38%) | 1,184 (25%) | 1,755 (17%) | 4,264 (29%) |  | 13,288 (21%) |
|  | 4-(Methylnitrosamino)-1-(3-pyridyl)-1-butanol [NNAL] | urine |  | 0 (---) | 0 (---) | 0 (---) | 0 (---) | 4,064 (30%) |  | 4,064 (30%) |
|  |  |  |  |  |  |  |  |  |  |  |
|  |  |  |  |  |  |  |  |  |  |  |
| Abbreviations: ANA = antinuclear antibodies; LOD = limit of detection; NHANES = National Health and Nutrition Examination Survey. | | | | | | | | | | |
| ^a^ All of these perfluoroalkyl and polyfluoroalkyl substances were measured in serum, with concentration units of μg/L (or equivalently ng/mL). | | | | | | | | | | |
| ^b^ Perchlorate and other anions were measured in urine, with concentration units of μg/L (or equivalently ng/mL). | | | | | | | | | | |
| ^c^ All of these personal care and consumer product chemicals and metabolites were measured in urine, with concentration units of μg/L (or equivalently ng/mL). | | | | | | | | | | |
| ^d^ Cotinine was measured in serum and NNAL was measured in urine, both with concentration units of μg/L (or equivalently ng/mL). | | | | | | | | | | |
